# The postoperative analgesic efficacy of different regional anesthesia techniques in breast cancer surgery: A network meta-analysis

**DOI:** 10.3389/fonc.2023.1083000

**Published:** 2023-03-28

**Authors:** Ran An, Dan Wang, Xiao-Long Liang, Qi Chen, Qian-Yun Pang, Hong Liang Liu

**Affiliations:** ^1^ Department of Anesthesiology, Chongqing University Cancer Hospital, Chongqing, China; ^2^ Chongqing Key Laboratory of Translational Research for Cancer Metastasis and Individualized Treatment, Chongqing University Cancer Hospital, Chongqing, China

**Keywords:** regional anesthesia, postoperative, analgesic efficacy, breast cancer surgery, network meta-analysis

## Abstract

**Background:**

Regional anesthesia have been successfully performed for pain management in breast cancer surgery, but it is unclear which is the best regional anesthesia technique. The aim of the present network meta-analysis was to assess the analgesic efficacy and disadvantages of regional anesthesia techniques.

**Methods:**

Multiple databases were searched for randomized controlled trials (RCTs). The association between regional anesthesia and analgesic efficacy was evaluated by Bayesian network meta-analysis.

**Results:**

We included 100 RCTs and 6639 patients in this study. The network meta-analysis showed that paravertebral nerve block, pectoral nerve-2 block, serratus anterior plane block, erector spinae plane block, rhomboid intercostal block, and local anesthetic infusion were associated with significantly decreased postoperative pain scores, morphine consumption and incidence of postoperative nausea and vomiting compared with no block. Regarding the incidence of chronic pain, no significance was detected between the different regional anesthesia techniques. In the cumulative ranking curve analysis, the rank of the rhomboid intercostal block was the for postoperative care unit pain scores, postoperative 24-hour morphine consumption, and incidence of postoperative nausea and vomiting.

**Conclusion:**

Regional anesthesia techniques including, paravertebral nerve block, pectoral nerve-2 block, serratus anterior plane block, erector spinae plane block, rhomboid intercostal block, and local anesthetic infusion, can effectively alleviate postoperative acute analgesia and reduce postoperative morphine consumption, but cannot reduce chronic pain after breast surgery. The rhomboid intercostal block might be the optimal technique for postoperative analgesia in breast cancer surgery, but the strength of the evidence was very low.

**Systematic review registration:**

https://www.crd.york.ac.uk/prospero/(PROSPERO), identifier CRD 42020220763.

## Introduction

There were an estimated 276,480 new cases of invasive breast cancer in 2020, and breast surgery was the primary treatment (1). Nearly half of the surgical patients experience moderate to severe acute pain after surgery, and 8-25% of them develop chronic pain (2–6). Several risk factors have been identified and these include severe preoperative pain, severe acute postoperative pain, surgical factors such as the number of lymph nodes removed and the complexity of the operation, previous or concurrent radiotherapy or chemotherapy, obesity, depression or anxiety, and age (4, 7–9). Recho K et al. study showed that 57.7% of patients had pain scores less than 5 points in the PACU (2). So we urgently need to find ways to reduce postoperative pain in breast cancer surgery. Postoperative pain can delay patients’ rehabilitation, extend hospitalization and induce psychological illness (6, 10, 11).

Currently, systemic opioids are routinely administered for analgesia after breast cancer surgery, and side effects often occur, including respiratory depression, sedation, postoperative nausea and vomiting (PONV), pruritus, urinary retention, constipation, and even addiction (12, 13). Poor postoperative pain control is one of the leading causes of opioid abuse (13–16). Several regional anesthesia techniques have been implemented for breast cancer surgical patients in recent years. In the PROPECT guidelines for breast surgery, paravertebral block (PVB) is recommended as the first-choice method for analgesia during breast surgery (17), but some other regional anesthesia techniques, such as pectoral nerve block (PECS), erector spinae plane (ESPB) and serratus anterior plane block (SPB), have also been studied. In some meta-analyses, ESPB, PECS, and SPB provided better analgesic effects than no block and provided similar analgesia to PVB in breast surgery (18–20), but it is unclear which is the best regional anesthesia technique (18, 20, 21). Recently, two network analyses showed that PVB, PECS-2 block, ESPB, SPB, and local anesthetic (LA) infusion reduced postoperative pain scores and morphine consumption after breast surgery (22, 23); furthermore, PVB and SPB had a high probability of reducing pain at 24 hours after major breast cancer surgery. Breast cancer surgery and benign breast surgery were included in these network meta-analyses, but different surgical procedures are associated with different levels of postoperative pain. Radical mastectomy surgery might lead to more trauma and pain. Furthermore, other regional anesthesia techniques, including interscalene brachial plexus block (IPB), SPB with PECS-1 block, SPB with PECS-2 block, and rhomboid intercostal nerve block (RIB), have been reported in breast cancer surgery in recent years (24–26), To date, it is unclear which technique can provide more effective analgesia in breast cancer surgery. We conducted this network meta-analysis to assess the efficacy of all regional anesthesia techniques for postoperative analgesia in breast cancer surgery, and then determined the optimal analgesic method for postoperative acute pain and chronic pain.

## Materials and methods

We conducted our network meta-analysis following the methods recommended by the Preferred Reporting Items for Systematic Reviews and Meta-analysis (PRISMA) guidelines (27). This network meta-analysis was registered at https://www.crd.york.ac.uk/prospero/(PROSPERO) with the registration number CRD 42020220763.

### Search strategy

The PubMed, Embase, and Cochrane Library databases were searched with English restrictions and available full text through May 2022. **Appendix 1** provides details about the search strategy in PubMed. The Clinical Trials Registry was searched for unpublished trials. Moreover, the references cited in the retrieved literature were searched to identify any additional eligible trials.

### Inclusion and exclusion criteria

Inclusion criteria: randomized clinical trials that evaluated pain management, morphine consumption, and quality of recovery (e.g., nausea and vomiting) after breast cancer surgery, using regional anesthesia, and included the following items (1): All patients undergoing general anesthesia, (2) all patients with ASA I-III, (3) in the no block group, no regional anesthesia was given or only normal saline was used for regional anesthesia.

Exclusion criteria: (1) age under 18 years, (2) surgery for breast augmentation or breast reconstruction, (3) nonopioid analgesics systemically administered after breast cancer surgery, including NSAIDS and tramadol, and (4) the primary data could not be extracted.

### Primary outcomes

(1) Acute postoperative pain (rest), (2) Postoperative morphine consumption, (3) Incidence of chronic pain, and (4) Incidence of postoperative nausea and vomiting (PONV).

### Data extraction

Two researchers (Ran An, Dan Wang) reviewed and extracted data from the included articles. We collected the following data: first author, year of publication, type of intervention in each group, the sample size in each group, type of surgery, nerve block under ultrasound or not and postoperative pain scores at rest, postoperative morphine consumption, and the number of postoperative chronic pain, PONV, and adverse events.

Pain scores were usually presented on a numeric rating scale (NRS), ranging from 0 to 10, and a visual analog scale (VAS), ranging from 0 to 100. We converted the VAS to NRS by dividing the results by 10. Opioids were converted to morphine in an equivalent dose using a standardized conversion calculator(https://clincalc.com/Opioids/). When the pain scores, postoperative morphine consumption, or outcomes were not presented numerically in the included article, the corresponding author was mailed asking for more detailed information. Meanwhile, Engauge Digitizer software (Version 4.1, Mitchell, http://markummitchell.github.io/engauge-digitizer/) was used to extract data from graphs or images if the data were presented as figures. We considered adding 0.05 or 1 in each group when the outcomes were 0 or no events (28). When the interquartile range (IQR) was presented, we regarded IQR as the mean and IQR/1.135 as the SD (29). If the Min-Max median was presented in eligible articles, the data were not considered for statistical analysis. When only postoperative nausea was reported, the incidence of postoperative nausea represented that of PONV.

### Risk of bias assessment

We assessed the quality of the eligible articles with the Cochrane Collaboration’s tool. Confidence in Network Meta-analysis (CINeMA 0.6.1 version) was used to evaluate the certainty of confidence contributing to the network meta-analysis based on the five essential elements of the GRADE, which included instruction, study limitations, imprecision, inconsistency (heterogeneity and incoherence), indirectness and publication bias (30).

### Data synthesis and analysis

We conducted pairwise meta-analyses in STATA 17.0, using DerSimonian-Laird random-effect models for each treatment comparison. The variables were extracted as the mean difference (MD) for continuous variables and odds ratios (ORs) for dichotomous outcomes. Effect sizes were accompanied by 95% confidence intervals (CIs).

We performed a random-effects network meta-analysis with a Bayesian setting. The MD for each outcome with a 95% Credible Interval (95% CrI) was summarized. We estimated the ranking probabilities for each intervention for all treatments (31). The treatment hierarchy was summarized and reported as the surface under the cumulative ranking curve (SUCRA) and mean ranks, which was considered a secondary endpoint. SUCRA was a percentage interpreted as the probability of treatment efficacy, which equaled 1 when the treatment was considered the best with certainty and 0 when it was the worst. All analyses were conducted using R 3.6.2(gemtc package, network meta-analysis, network regression, sensitive analysis, assessment of global heterogeneity) and STATA 17.0 (http://www.mtm.uoi.gr/index.php/stata-routines-for-network-meta-analysis).

### Examination of assumptions in the network meta-analysis (consistency, transitivity, and heterogeneity)

We used a design-by-treatment approach to check the assumption of consistency in the entire analytical network. A loop-specific approach was used to evaluate the presence of local inconsistency (32). The node-splitting method was used to evaluate the inconsistency of the model by separating evidence on a particular comparison into direct and indirect evidence (33). Global heterogeneity was evaluated with an I^2^ statistic that incorporated the extent of heterogeneity and was used to evaluate the extent of uncertainty in the estimated effect size locally (34). The transitivity assumption underlying the network meta-analysis was evaluated by comparing the distribution of the clinical variables. Univariable network meta-regression in the context of the Bayesian framework was further conducted to examine the potential modification effects of age and type of surgery. Besides, the sensitivity analysis of network meta-analysis was narrowed into trials with the use of ultrasound to validate the robustness of the results.

## Results

**Figure 1:**
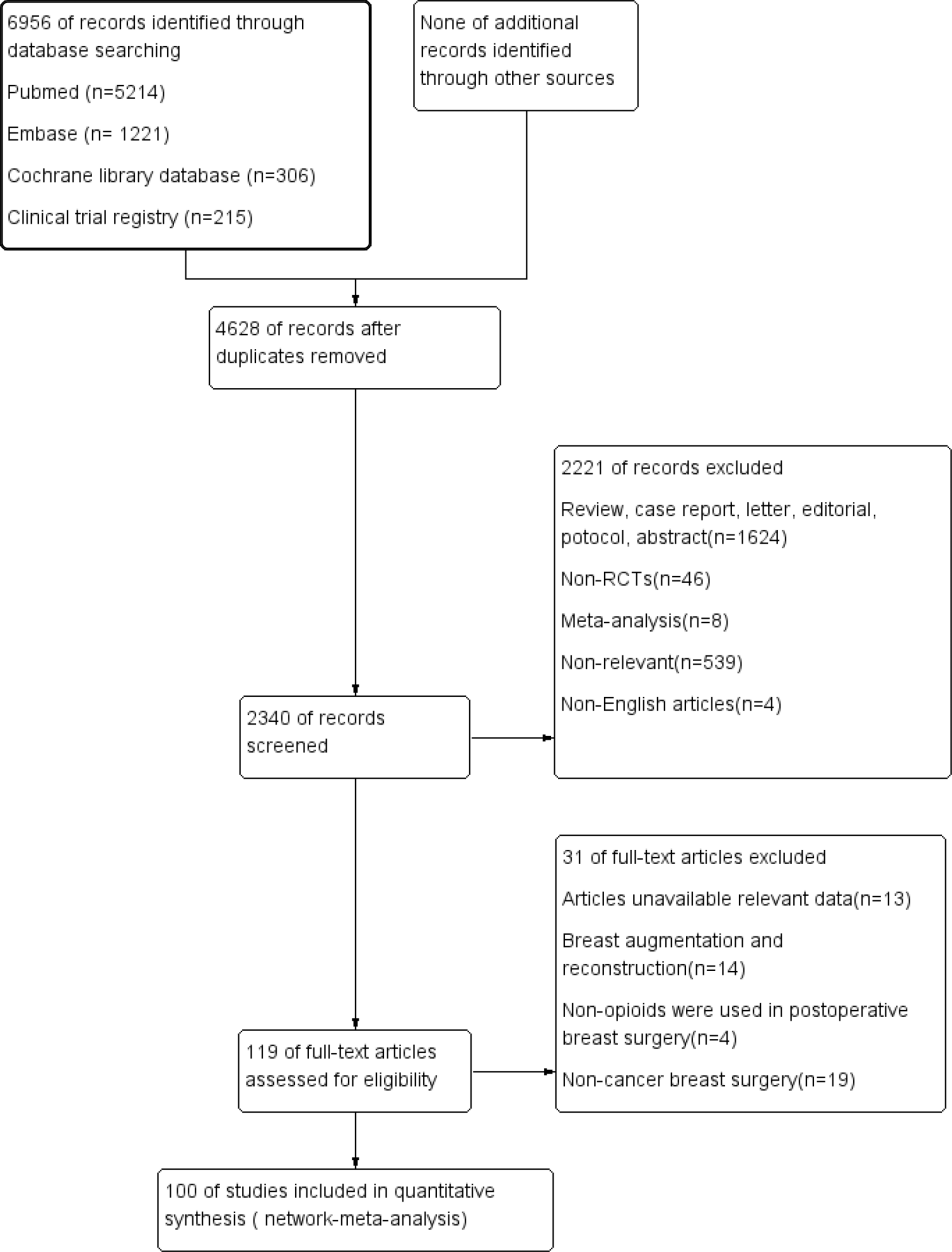
Flow chart of literature screening.

We identified 6956 citations from the databases. Based on the eligibility criteria, 100 studies (6639 patients) were ultimately included in the network meta-analysis. The search strategy and results are shown in **Appendix 1**. The flow chart of the literature screening process is shown in **Figure 1**.

The basic characteristics of the enrolled studies are described in **Appendix 2**. Ten regional anesthesia techniques were reported, including PVB, PCES-2 block, PECS-1 block, ESPB, SPB, RIB, IPB, LA infusion, SPB with PECS-1 block, and SPB with PECS-2 block. Normal saline or placebo was injected as no block.

Sixty-one trials reported radical mastectomy surgery in the type of surgery. The type of breast cancer surgery was unclear in thirty-two trials, six trials reported 6 mastectomy with sentinel lymph node biopsy in the type of surgery and, only one trial reported breast-conserving surgery and sentinel lymph. References for included trials and characteristics was shown in **Appendix 2**.

The risk of bias is presented in **Appendix 3**. Thirty-five trials were ranked as high risk, twelve trials were ranked as moderate risk, and fifty-three trials were ranked as low risk. The most common risk was incomplete blinding of the participants and personnel and allocation concealment.

### PACU pain scores (rest)

**Figure 2:**
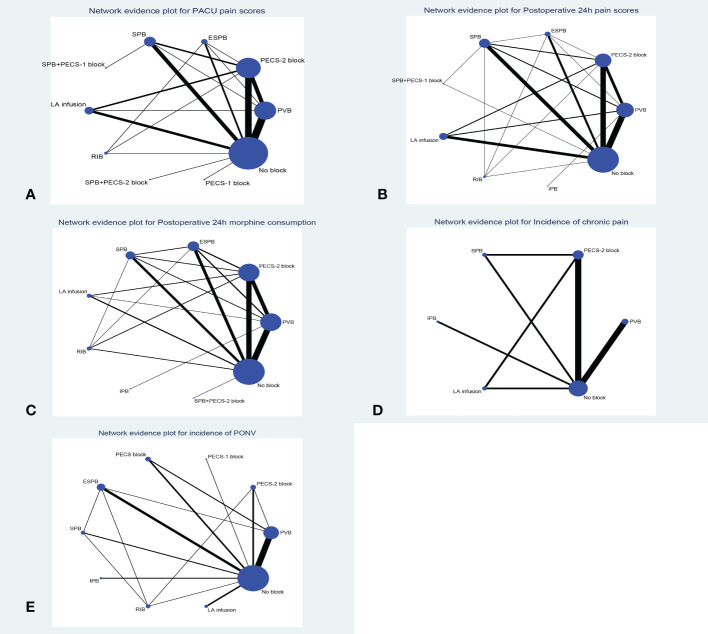
Network geometry plot **(A)**: PACU pain scores, **(B)**: Postoperative 24h pain scores, **(C)**: Postoperative 24h morphine consumption, **(D)**: Incidence of chronic pain, **(E)**: Incidence of PONV). Lines connect the interventions that have been studies in direct comparison in the eligible RCTs. The width of the lines represents the cumulative number of RCTs for each pairwise comparison and the size of every node is proportional to the number of randomized participants. ESPB, erector spinae plane block; PECS-2 block, pectoral nerves-2 block; PECS-1 block, pectoral nerve-1 block; PVB, paravertebral nerve block; SPB, serratus anterior plane block; IPB, interpleural block; LA infusion, local anesthetic infusion).

**Figure 3:**
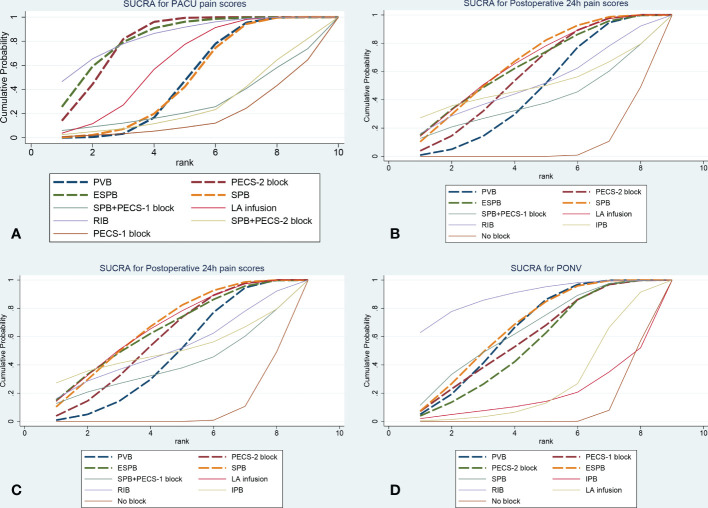
The plot of cumulative ranking curve **(A)**: PACU pain scores, **(B)**: Postoperative 24h pain scores, **(C)**: Postoperative 24h morphine consumption, **(D)**: Incidence of PONV). The area under the curve is proportional to SUCRA. ESPB, erector spinae plane block; PECS-2 block, pectoral nerves-2 block; PECS-1 block, pectoral nerve-1 block; PVB, paravertebral nerve block; SPB, serratus anterior plane block; IPB, interpleural block; LA infusion, local anesthetic infusion).

Forty-nine trials (3282 patients) reported pain scores in the PACU. The results network meta-analysis showed that PVB [-1.49, 95% CrI (-2.05, -0.94)], PECS-2 block [-2.2, 95% CrI (-2.74, -1.67)], ESPB [-2.3, 95% CrI (-3.27, -1.3)], SPB [-1.43 95% CrI (-2.21, -0.64), LA infusion [-1.77, 95% CrI (-2.61, -0.93)] and RIB [-2.47, 95% CrI (-4, -0.91)] were associated with a significant decrease in PACU pain scores compared with the no block group. Furthermore, the PECS-2 block was associated with a significant decrease in pain scores compared with PVB [-0.71 95% CrI (-1.32, -0.1)]. RIB (83.8%) was ranked the highest, which was based on SUCRA scores. **Figure 2** shows the geometry of the network for PACU pain scores. The cumulative ranking is shown in **Figure 3** and **Appendix 5**. The results of the direct meta-analysis and SUCRA scores are shown in **Appendix 4**, **6**.

### Postoperative 24-hour pain scores (rest)

Sixty-eight trials (4672 patients) reported postoperative 24-hour pain scores. The Results of the network meta-analysis showed that PVB [-0.63, 95% CrI (-0.97, -0.29], PECS-2 block [-0.75, 95% CrI (-1.11, -0.39)], ESPB [-0.8, 95% CrI (-1.39, -0.2)], SPB [-0.77, 95% CrI (-1.19, - 0.34) and LA infusion [-0.82, 95% CrI (-1.31, -0.33)] were associated with a significant decrease in pain scores compared with the no block group at postoperative 24 hours. SPB (66.3%) was ranked the highest, which was based on SUCRA scores. **Figure 2** shows the geometry of the network for postoperative 24-hour pain scores. The cumulative ranking is shown in **Figure 3** and **Appendix 5**. The results of the direct meta-analysis and SUCRA scores are shown in **Appendix 4**, **6**.

### Postoperative 24-hour morphine consumption

Sixty-two trials (3724 patients) reported postoperative 24-hour morphine consumption. The results of the network meta-analysis showed that PVB [-7.14, 95% CrI (-9.78, -4.17], PECS-2 block [-8.81, 95% CrI (-11.43, -6.15)], ESPB [-7.93, 95% CrI (-11.29, -4.44)], SPB [-8.5, 95% CrI (-12.37, - 4.61), LA infusion [-8.93, 95% CrI (-14.29, -3.58)] and RIB [-10.46, 95% CrI (-16.43, -4.58)] were associated with a significant decrease in pain scores compared with the no block group at postoperative 24-hour of morphine consumption. RIB (79.5%) was ranked the largest, which was based on SUCRA scores. **Figure 2** showed the geometry of the network for 24-h postoperative morphine consumption. The cumulative ranking is shown in **Figure 3** and **Appendix 5**. The results of the direct meta-analysis and SUCRA scores are shown in **Appendix 4**, **6**.

### Incidence of chronic pain (3 months postoperatively)

Eight trials (871 patients) reported the incidence of chronic pain 3 months postoperatively. No significance was detected among the different regional anesthesia techniques by pairwise meta-analysis and network meta-analysis, and we did not perform statistical analysis of the SUCRA. **Figure 2** shows the geometry of the network for the incidence of chronic pain. The results of the direct meta-analysis are shown in **Appendix 4**.

### PONV (postoperative 24-hour)

Thirty-three trials (1879 patients) reported PONV (postoperative 24 hours). The results of the network meta-analysis showed that PVB [OR=0.35, 95% CrI (0.22,0.53], PECS-2 block [OR=0.37, 95% CrI 0.16,0.81)], ESPB [OR=0.32, 95% CrI (0.18,0.58)], SPB [OR=0.33, 95% CrI (0.14, 0.76) and RIB [OR=0.2, 95% CrI (0.07,0.54)] were associated with a significant decrease in PONV compared with the no block group at postoperative 24 hours. RIB was ranked the highest (88.8%), which was based on SUCRA scores. **Figure 2** shows the geometry of the network for the incidence of PONV. The cumulative ranking is shown in **Figure 3** and **Appendix 5**. The results of the direct meta-analysis and SUCRA scores are shown in **Appendix 4**, **6**.

### Adverse events

Fifteen trials reported adverse events from the regional anesthesia, including pneumothorax, pruritus, dizziness, hypotension, and bradycardia. No studies reported vascular puncture or nerve injury. We did not perform statistical analysis, because the incidence of adverse events was low and there was no between-group difference in any of these trials.

### GRADE evaluation of the quality of evidence

CINeMA version 0.6.1 was used to evaluate the GRADE level. The results of network the meta-analysis was mostly moderate to very low. For the PACU and postoperative 24-hour pain scores, the quality was moderate. In the postoperative 24-hour morphine consumption, the quality was very low. For the incidence of chronic pain and PONV, the quality was low. The evaluation of the quality of evidence using Cochrane Collaboration’s tool and Confidence in Network Meta-analysis is presented in **Table 1** and **Appendix 14**.

**Table 1:** The GRADE quality of evidence assessment for the outcomes in results of network meta-analysis.

Outcome	Number of trials	Number of interventions	Conclusions	Quality of evidence	Comments
PACU pain scores	49 trials (3282 patients)	10	PVB, PECS-2 block, ESPB, SPB, RIB, and LA infusion were associated with a decrease in pain scores compared with no block. RIB was the largest on SUCRAS scores	⊕⊕⊕ Moderate quality	Downgraded for concerns related to imprecision
Postoperative 24h pain scores	68 trials(4672piatents)	9	PVB, PECS-2 block, ESPB, SPB, and LA infusion were associated with a decrease in pain scores compared with no block. SPB was the largest on SUCRA scores	⊕⊕⊕ Moderate quality	Downgraded for concerns related to imprecision and Heterogeneity
Postoperative 24h morphine consumption	62 trials (3724 patients)	10	PVB, PECS-2 block, ESPB, SPB, RIB, and LA infusion were associated with a decrease in pain scores compared with no block. RIB was the largest on SUCRAS scores	⊕ Very low quality	Downgraded for concerns related to within-study bias, Imprecision, and Incoherence
Incidence of pain (postoperative 3 months)	8 trials (871 patients)	5	No significance was detected between different regional anesthesia techniques	⊕⊕ Low quality	Downgraded for concerns related to imprecision and heterogeneity
PONV (postoperative 24h)	33 trials (1879)	9	PVB, PECS-2 block, ESPB, SPB, and RIB were associated with a decrease of PONV with no block. RIB was the largest on SUCRAS scores	⊕⊕ Low quality	Downgraded for concerns related to within-study bias and imprecision

⊕⊕⊕(Moderate quality): We are moderately confident in the effect estimate: the true effect is likely to be close to the estimate of the effect, but there is a possibility that it is substantially different. ⊕⊕(Low quality): Our confidence in the effect estimate is limited: the true effect may be substantially different from the estimate of the effect. ⊕(Very low quality): We have very little confidence in the effect estimate: the true effect is likely to be substantially different from the estimate of effect. The meaning of the symbols from the GRADE Handbook.

### Transitivity, inconsistency, and heterogeneity

We had strict inclusion and exclusion criteria; however, we did not have sufficient methods to properly assess the transitivity assumption. The test of global inconsistency did not detect any significant difference between the consistency and inconsistency modes for the outcomes. The test of inconsistency from the node-splitting model and inconsistency plots showed no significance in most outcomes. The funnel plot showed that the comparison of dots was quite symmetrically distributed on both sides of point 0 and did not suggest any significant risk of publication bias for the included studies. The assessment of the global inconsistency results is presented in **Appendix 7**. The assessment of local inconsistency results by the node-splitting method is presented in **Appendix 8**. The evaluation of inconsistency using loop-specific heterogeneity estimates is presented in **Appendix 9**. The comparison-adjusted funnel plot results are presented in **Appendix 10**.

### Sensitivity analysis and meta-regression

Sensitivity analysis of network meta-analysis by narrowing into trials with the use of ultrasound, the results did not change significantly, and the results are presented in **Appendix 12**. Furthermore, sensitivity analysis utilizing R software found stable results (35), and the results are presented in **Appendix 11**. Meta-regression indicated that the type of breast cancer surgery might affect the PACU pain score in the PECS-1 block, and the results are presented in **Appendix 13**.

## Discussion

Postoperative acute pain and chronic pain are common after breast cancer surgery, and uncontrolled postoperative pain might lead to increased social and economic burdens. In our study, ten regional anesthesia techniques currently used in the clinic were analyzed to compare their analgesic efficacy for breast cancer surgery. PVB, ESPB, SPB, PECS-2 block, RIB, and LA infusion could provide better postoperative analgesia, and reduce postoperative 24-hour morphine consumption and the incidence of PONV. Although regional anesthesia significantly reduced the postoperative 24-hour pain scores, the difference did not reach a not minimal clinically important difference (36). Local anesthetic techniques can play an important role in reducing postoperative pain and morphine consumption in other thoracic surgery, and some of the regional anesthesia techniques from the included trials for breast cancer surgery, For example, PVB, ESPB, and PECS block were used in cardiac surgery or thoracic surgery. Our results provide a clinical basis and a direction for research into the use of regional anesthesia in patients undergoing other types of surgery, especially thoracic surgery.

In contrast, postoperative pain scores and morphine consumption was not reduced by PECS-1 block, IPB, SPB with PECS-1 block, or SPB with PECS-2 block. To date, none of the included regional anesthesia techniques have reduced the incidence of postoperative chronic pain. No difference was detected among the different regional anesthesia methods in terms of anesthesia effects.

The breast is innervated by lateral and anterior cutaneous branches of the second to sixth thoracic intercostal nerve branches and several branches of the supraclavicular nerve (37, 38). No regional anesthesia technique alone can cover the whole innervated area. We had believed the combination of regional anesthesia may have a better anesthetic effect and a lower incidence of PONV in breast cancer surgery. However, our study did not find a better analgesic effect with the combination of regional anesthesia in the Treatment’s efficacy (league) tables (**Appendix 5**). We only included two combinations for comparison, including SPB+PECS-1 and SPB+PECS-2 blocks. Other combinations of regional anesthesia such as RIB+ PECS block, PVB+ RIB, ESPB + PECS block, etc were not reported. The combination of the regional need to be further investigated.

It has been reported that PECS blocks consist of three types (PECS-1 block, PECS- 2 block, and SPB) (39–41), and the long thoracic nerves, thoracodorsal nerves, and intercostal nerves can be blocked by a PECS-2 block (40). Several meta-analyses showed that the PECS-2 block could effectively reduce postoperative pain scores and morphine consumption in breast surgery, and it was recommended as the first-line analgesic technique for breast surgery (19, 32, 42). In our network meta-analysis, PECS-2 block and SPB reduced postoperative pain scores and morphine consumption. Moreover, PECS-2 block and SPB reduced the incidence of PONV. PECS-1 blockade might not be appropriate for pain management in breast cancer surgery. PECS-1 block can only block the lateral pectoral and medial pectoral (39). Furthermore, our study showed that multiple block techniques, including SPB with PECS-1 block and SPB with PECS-2 block, did not exhibit a superiority anesthesia effect by indirect comparison and cumulative ranking probability. However, only 4 studies were included, and more research is needed regarding the simultaneous use of multiple block techniques in breast cancer surgery.

PVB was most widely used in breast surgery, and the injection of local anesthetic into the paravertebral space blocks the anterior and posterior branches of the intercostal nerves, the sympathetic trunk, and the rami communis (43, 44). One meta-analysis showed that PVB could significantly reduce postoperative pain scores and morphine consumption (45). Our network meta-analysis found that PVB was an effective analgesic technique in breast cancer surgery. However, the lateral and inner pectoralis nerve cannot be blocked by PVB, so it is a suboptimal analgesic technique. Furthermore, our network meta-analysis showed that PECS-2 block decreased PACU pain scores, but the decrease did not achieve a minimal clinically important difference (36).

ESPB was described in 2016 as a new regional anesthesia technique (46). The potential mechanisms included the following:1) The LA exerts its effect on the ventral and dorsal ramus of the spinal nerve. 2) The diffusion of LA to the paravertebral space through the costotransverse foramina and the intertransverse complex provides similar efficacy as PVB (46). One meta-analysis showed that ESPB reduced postoperative pain scores and morphine consumption compared with no block. Furthermore, ESPB exerts the same analgesic efficacy as PVB (47). In our study, ESPB had the same effective analgesic techniques compared to PVB in breast cancer surgery.

LA infusion provides analgesia by blocking the transmission of nociception and inhibiting local inflammatory responses (48). LA infusion significantly reduced postoperative pain scores and morphine consumption. Wang HY et al.’s network analysis showed that nerve blocks were preferable from an analgesic perspective to LA infusion, but the results of our study showed that nerve block and LA infusion provide similar efficacy of anesthesia after breast cancer surgery. The differences in the results may come from the different inclusion criteria and the heterogeneity of the studies themselves, and we need to explore further the homogeneity of the local anesthesia techniques for breast cancer surgery.

Rhomboid intercostal block (RIB) was first described by Elsharkawy et al. in 2016 (49). RIB showed the spread of the dye from caudad to cephalad, including the T2 to T8 tissue plane, as far as lateral branches of the intercostal nerves T3 to T8, the posterior primary rami near the midline, and the clavipectoral fascia within the axilla (50). Therefore, RIB may provide better anesthesia for axillary surgery; furthermore, the RIB is on the posterior chest wall, so it is far away from the surgical site. In the SUCRA analysis, RIB had the highest cumulative rank for decreasing PACU pain scores, postoperative 24-hour morphine consumption, and incidence of PONV. The 2020 guideline recommends PVB and PECS block as recommended for breast cancer pain management, but the guideline does not discuss the possibility of the RIB (17). RIB is the most recently discovered method of intrafascial block and there are few randomized clinical trials. Only three RCT trials were included in our study. Our study used SUCRA to find that RIB may be the optimal regional anesthesia method, but SUCRA is only a probabilistic possibility. Some scholars consider rankogram and SUCRA, to provide the opportunity to determine the best available treatment. However, one must be cautious in interpreting the SUCRA, as high values may only provide supportive, but not decisive, evidence of treatment choice (51). Our study provides a clinical basis and the possibility of further research to reduce postoperative pain and morphine consumption in breast cancer surgery. More clinical trials are needed to explore its efficacy in the future.

The mechanism underlying postoperative chronic pain after breast surgery is multifaceted, and included surgery-related nerve injury and nerve entrapment, axillary hematoma, or the development of a traumatic neuroma at the operated site (4, 52). One previous meta-analysis showed that PVB reduced the incidence of chronic pain after breast surgery (53). However, only two studies were included to support this result, and one was in not in English. Our results showed that regional anesthesia techniques did not reduce the incidence of postoperative chronic pain in breast cancer surgery, and 2 studies published in English were included to support this result. Above all, there are currently too few studies on chronic pain, and more clinical trials should be conducted.

Our study showed that PVB, PECS-2 block, ESPB, SPB, or RIB could decrease the incidence of PONV after breast cancer surgery. Many factors contribute to the development of PONV, such as postoperative opioid use, sex, non-smoking, and history of PONV (54). However, using morphine was an important risk factor in PONV. In the SUCRA analysis, RIB had the highest cumulative rank in decreasing PONV.

Regional anesthesia might cause some complications, such as pneumothorax, vascular puncture, nerve injury, hypotension, and hematoma. Their incidences in the included studies were very low, so the statistical analysis was not conducted. The main side effects of the paravertebral block are hypotension and pneumothorax, with 4% and 0.5%, respectively. The intrafascial block and LA infusion-related complications are rare.

This network meta-analysis has several limitations: 1) The choices of local anesthetics varied among the included studies, although the results were compatible within each study. 2) The sample size was small in some studies, and a high risk of bias existed. 3) The results of some outcomes were derived from general indirect comparisons of interventions with possible confounders. 4) Different general anesthetic agents were administered in the included studies for general anesthesia, which might exert potential interference with our network meta-analysis, although the results were compatible in each of the included studies. 5) SUCRA is only a probabilistic possibility. The low quality of the included literature and subjectivity in pain scoring could easily lead to large heterogeneity.

## Conclusions

Our network meta-analysis showed that PVB, PECS-2 block, SPB, ESPB, RIB, and LA infusion could effectively alleviate postoperative analgesia and reduce the incidence of PONV in breast cancer surgery. However, regional anesthesia techniques cannot effectively reduce chronic postoperative pain. Based on the SUCRA analysis, RIB might be the optimal technique for postoperative analgesia in breast surgery, but the strength of the evidence was very low.

## Data availability statement

The original contributions presented in the study are included in the article/**Supplementary Material**. Further inquiries can be directed to the corresponding author.

## Author contributions

RA, DW, and QC participated in selecting the study and extracting data, RA, X-LL, and Q-YP performed the statistical analysis and drafting of the manuscript, and HL participated in conceptualization, formal analysis, and drafting of the manuscript. All authors contributed to the article and approved the submitted version.
