# Supplementary material for: The postoperative analgesic efficacy of different regional anesthesia techniques in breast cancer surgery: A network meta-analysis
Source: Front Oncol. 2023 Mar 28;13:1083000. doi: 10.3389/fonc.2023.1083000 (PMC10088371; doi:10.3389/fonc.2023.1083000)
Supplement: Supplementary file 1 [file DataSheet_1.docx]

**Table of contents**

**Section title**

Appendix 1: Inclusion criteria and search strategy……………….…………………2

Appendix 2: References for included trials and characteristic………….……..7

Appendix 3: Risk of bias assessment……………………………………………………..25

Appendix 4: Direct pairwise meta-analysis…………………………………………….31

Appendix 5: Treatment’s efficacy(league) tables………………………………….…36

Appendix 6: Cumulative ranking probability (SCURA)……………………………..39

Appendix 7: Assessment of global inconsistency results……………………….…41

Appendix 8: node-splitting method………………………………………………………...46

Appendix 9: Loop-specific heterogeneity estimates…………………………………52

Appendix 10: Comparison-adjusted funnel plot……………………………………... 57

Appendix 11: Sensitivity analysis ………………….………………………………………….66

Appendix 12: Results of sensitivity analysis by only including trials with

used of ultrasound…………………………………………………………….. 75

Appendix 13: Meta-regression…………………………..………………………………………79

Appendix 14: evaluation of the quality of evidence…………………………………. 83

## Appendix 1

## search strategy

**Search strategy for PubMed**

1. breast[mh]
2. breast[all]
3. mastectomy, simple[mh]
4. mastectomy[all]
5. simple[all]
6. simple mastectomy[all]
7. mastectomy[mh]
8. Radical mastectomy[all]
9. Breast cancer surgey[all]
10. lymph node excision[mh]
11. Axilla/surgery[mesh]
12. surgery[subheading]
13. surgery[all]
14. surgical procedures, operative[mh]
15. surgical[all]
16. procedures[all]
17. operative[all]
18. operative surgical procedures[all]
19. general sugery[mh]
20. general[all]
21. general surgery[all]
22. (#1 OR #2 OR #3 OR #4 OR #5 OR #6 OR #7 OR #8 OR #9 OR #10 OR #11) AND (#12 OR #13 OR #14 OR # 15 OR #16 OR # 17 OR #18 OR #19 OR #20 OR #21)
23. nerve block[mh]
24. nerve block[all]
25. block[all]
26. anesthetics, local[mh]
27. anesthetics,local[all]
28. conduction block[all]
29. sensory nerve block[all]
30. peripheral nerves block[all]
31. neurogenic blockade[all]
32. local anaesthe*[all]
33. regional analgesia*[all]
34. PVB[all]
35. TPVB[all]
36. thoracic paravertebral block[all]
37. PVBS[all]
38. TPVBS[all]
39. PECS block[all]
40. PECS[all]
41. Pectoral nerve block[all]
42. PECS I [all]
43. PECS Ⅱ[all]
44. Erector spinae plane block[all]
45. Erector spinae[all]
46. ESPB[all]
47. SPB[all]
48. Serratus plane block[all]
49. #23 OR #24 OR #25 OR #26 OR #27 OR #28 OR #29 OR #30 OR #31 OR #32 OR #33 OR #34 OR #35 OR #36 OR #37 OR #38 OR #39 OR #40 OR #41 OR #42 OR #43 OR #44 OR #45 OR #46 OR #47 OR #48
50. pain, postoperative[mh]
51. neuralgia[mh]
52. causalgia[mh]
53. somatosensory[mh]
54. somatosensory disorder[mh]
55. pain[all]
56. postoperative pain[all]
57. postsurgical pain[all]
58. analgesi*[all]
59. anaesthe*[all]
60. vas[all]
61. nrs[all]
62. visual analog*[all]
63. postoperative morphine[all]
64. postoperative nausea and vomiting[all]
65. PONV[all]
66. postoperative*[all]
67. postoperative adverse event[all]
68. adverse event[all]
69. #48 OR #49 OR #50 OR #51 OR #52 OR#53 OR #54 OR #55 OR #56 OR #57 OR #58 OR #59 OR #60 OR #61 OR #62 OR #63 OR #64 OR #65 OR #66 OR #67 OR #68
70. randomized controlled trial[pt]
71. randomized controlled trial[all]
72. random*[all]
73. #70 OR #71 OR #72
74. #22 AND #49 AND #69 AND #73

## Appendix 2

## References for included trials and characteristic

**Table 1. Characteristics of the 100 studies included in the network Meta-analysis.**

| Author | Procedure | Age  Treatment/control | Number of treatment/control | Used of ultrasound | Interventions | Outcomes |
| --- | --- | --- | --- | --- | --- | --- |
| Ja’bari AA 2019(1) | Unilateral radical mastectomy surgery | 55±11/54±16 | 19/21 | Y | PECS-2 block (0.5 ropivacaine 30 ml)/ no block | Postoperative morphine consumption, VAS, PONV |
| Altıparmak B 2019(2) | Radical mastectomy surgery | 53.56±11.36/57.55/8.69 | 18/20 | Y | PECS-2 block (0.25% bupivacaine 10ml)/ESPB (0.25% ropivacaine 20ml) | Postoperative tramadol consumption, NRS |
| Kulhari S 2016(3) | Radical mastectomy surgery | 51±26/54±21 | 20/20 | Y | PVB (0.5% ropivacaine 25ml)/PECS-2 block (0.5 ropivacaine 25ml) | Postoperative morphine consumption, NRS |
| Altiparmak B 2020(4) | Unilateral Radical mastectomy | 53.8±11.2/52±11.5 | 28/28 | Y | RIB (0.25 bupivacaine 30ml)/ no block | Postoperative morphine consumption, NRS |
| Barrington MJ 2019(5) | Radical mastectomy | 60±10.3/59.2±11.3 | 53/51 | Y | PECS-2 block (0.475% ropivacaine/ LA infusion | Postoperative morphine consumption |
| Yao Y 2019(6) | Radical mastectomy | 46.5±10.4/47.7±9.8 | 34/34 | Y | SPB (0.5% ropivacaine)/no block | Postoperative opioids consumption, VAS |
| Hamed IG 2020(7) | Radical mastectomy | 44.19 ± 5.76/45.24 ± 6.86 | 10/8 | Y | PVB (0.25 bupivacaine 20ml)/ PECS-2 block (0.25 bupivacaine 20ml) | Postoperative pethidine consumption, VAS |
| Kairaluoma PM 2004(8) | Breast cancer surgery | 55±11/52±8 | 30/30 | N | PVB (0.3ml/kg bupivacaine)/ no block | Postoperative opioids consumption, VAS, PONV |
| Aksu C 2019(9) | Breast cancer surgery | 51.76±9.03/ 51.52±10.79 | 25/25 | Y | ESPB (0.25% bupivacaine 20ml)/ no block | Postoperative morphine consumption, NRS, PONV |
| Moustfa MA 2020(10) | Modified radical mastectomy | 40-65 | 45/45 | Y | ESPB (0.25% bupivacaine 20ml)/ PVB (0.25% bupivacaine 20ml) | Postoperative morphine consumption |
| EI Ghamry MR 2019(11) | Modified radical mastectomy | 41±11.8/37.7±12.9 | 25/25 | Y | ESPB (0.25% bupivacaine 20ml)/ PVB (0.25% bupivacaine 20ml) | Postoperative morphine consumption, VAS, PONV |
| Seealam S 2020(12) | Modified radical mastectomy | 50.56±11.61/52.68±8.14 | 50/50 | Y | ESPB (0.25 bupivacaine 30ml)/no block | Postoperative morphine consumption |
| Sinha C 2019(13) | Modified radical mastectomy | 53.63±8.8/53.80±9.37 | 30/30 | Y | ESPB (0.2% ropivacaine 25ml)/ PECS-2 block (0.2% ropivacaine 25ml) | Postoperative morphine consumption, NRS |
| Gürkan Y 2020(14) | Radical mastectomy | 49.1±10.6/49.4±7.3 | 25/25/25 | Y | ESPB (0.25% bupivacaine 20ml)/PVB (0.25% bupivacaine 20ml)/no block | Postoperative morphine consumption, NRS, PONV |
| Gürkan Y 2018(15) | Breast cancer surgery | 49.6±11/49.8±10.5 | 25/25 | Y | ESPB (0.25% bupivacaine 20ml)/ no block | Postoperative morphine consumption, NRS, |
| Hetta DF 2016(16) | Radical mastectomy | 50±7/51 ± 8 | 32/32 | Y | PECS-2 block (0.25% bupivacaine 5ml)/ PVB (0.25% bupivacaine 5ml) | Postoperative morphine consumption, VAS |
| Pill ai VS 2018(17) | Radical mastectomy | 49.42±10.09/52±9.46 | 19/20 | Y | PECS-2 block (0.5% ropivacaine 20ml)/PVB (0.5 ropivacaine 20ml) | Postoperative morphine consumption |
| Syal K 2017(18) | Modified radical mastectomy | 46±7.33/44.5±6.43/44.53±6.1 | 22/21/22 | Y | PECS-2 block (0.5% bupivacaine 21ml)/ PVB (0.5% bupivacaine 21ml)/ no block | VAS |
| Siddeshwara A 2019(19) | Modified radical mastectomy | 45.96±14.92/46.54±15 | 20/20 | Y | PECS-2 block (0.25% levobupivacaine 24ml)/PVB (0.25% levobupivacaine 24ml) | Postoperative morphine consumption, NRS, PONV |
| Wahba SS 2019(20) | Modified radical mastectomy | 49.9±6.9/49.9±6.7 | 30/30 | Y | PECS-2 block (0.25% levobupivacaine 20ml)/PVB block (0.25% levobupivacaine15-20ml) | Postoperative morphine consumption, NRS, PONV |
| Bashandy GMN 2015(21) | Modified radical mastectomy | 48.65±10.7/50.47±12.1 | 60/60 | Y | PECS-2 block (0.25 levobupivacaine 20ml) / no block | Postoperative morphine consumption, VAS, PONV |
| CrosJ 2018(22) | Modified radical mastectomy | 60.5±12.6/62±10.37 | 62/66 | Y | PECS-1 block (0.25 bupivacaine 5-20ml/ no block | Postoperative morphine consumption, NRS, PONV |
| MN 2018(23) | Modified radical mastectomy | 50.53 ± 10.44/45.7 ± 11 | 30/30 | Y | PECS-2 block (0.25% ropivacaine/ no block | Postoperative morphine consumption，VAS， PONV |
| Hassn AMA 2016(24) | Modified radical mastectomy | 39±12.09/40.17±12.63 | 30/30 | Y | PECS-2 block (0.5 bupivacaine 30ml)/ no block | Postoperative morphine consumption, VAS, PONV, number of postoperative rescue analgesia |
| Kamiya Y 2017(25) | Modified radical mastectomy | 54.5±12/53.3±11.6 | 24/21 | Y | PECS-2 block (0.25 levobupivacaine 30ml)/ no block | NRS, PONV, number of postoperative rescue analgesia |
| O’Scanaill 2018(26) | Breast-cancer surgery | 57.6/59.5 | 15/15/15 | Y | PECS-2 block (0.25% levobupivacaine 20ml)/PECS-2 block+ LA infusion (0.1% levobupivacaine 10ml/h)/LA infusion (0.1% levobupivacaine 10ml/h)/ | Postoperative morphine consumption, NRS |
| Versyck B 2017(27) | Modified radical mastectomy | 59.62±10.66/58.8±9.66 | 45/40 | Y | PECS-2 block (0.25% levobupivacaine 10ml)/ no block | Postoperative morphine consumption, NRS |
| Tripathy S 2019(28) | Modified radical mastectomy | 52.4±12.3/ 51.8±10.5 | 29/29 | Y | PECS-2 block (2% lignocaine+ 0.5% bupivacaine+1ug/kg dexmedetomidine 30ml/PVB (1% lignocaine+ 0.5% bupivacaine+1ug/kg dexmedetomidine 30ml | PONV, VAS |
| Kim DH 2018(29) | Breast-conserving surgery and sentinel lymph | 45.4 ± 9.9/45.2 ± 11.9 | 40/38 | Y | PECS-2 block (0.25% ropivacaine 30ml)/no block | Postoperative morphine consumption, NRS |
| Bakeer AH 2020 (30) | Modified radical mastectomy | 50.8±8.9/ 50.9±6.8/ 50.4±9.3 | 57/58/58 | Y | PECS-2 block (0.25% bupivacaine 30ml)/SPB block (0.25% bupivacaine 30ml)/no block | Postoperative morphine consumption, VAS, number of postoperative rescue analgesia |
| Kaur U 2020(31) | Modified radical mastectomy | 45.7 ± 13.2/48.2 ± 10.4/ 51.0 ± 11.3 | 18/18/19 | Y | PECS-2 block (0.2% ropivacaine 30ml// SPB block (0.2% ropivacaine 0.4ml/kg)/no block | NRS |
| He W 2020(32) | Breast cancer surgery | 51.3± 8/ 50.50 ± 7.75 | 20/20 | Y | ESPB (0.5% ropivacaine 20ml)/no block | VAS |
| Thomas M 2018(33) | Modified radical mastectomy | 50.18±8.17/50.63±9.31 | 28/30 | N | PECS-2 block (0.2% ropivacaine 20ml)/no block | Postoperative morphine consumption |
| Bansal P 2012(34) | Modified radical mastectomy | 43.35±8.7/45.95±12.24 | 20/20 | N | PVB (0.25% bupivacaine 0.3ml/kg)/LA infusion (0.25% bupivacaine 0.3ml/kg | VAS |
| Singh S 2019(35) | Modified radical mastectomy | 46±25.1/45±29.6 | 20/20 | Y | ESPB (0.5 bupivacaine 20ml)/no block | Postoperative morphine consumption, NRS |
| IIfeld BM 2015(36) | Modified radical mastectomy | 48±10.37/49±12.6 | 30/30 | Y | PVB (0.5 ropivacaine 15ml)/ no block | Postoperative morphine consumption, NRS, PONV |
| Qian B 2019(37) | Modified radical mastectomy | 46.5±6.7/44.8±7.2 | 86/86 | Y | PVB (0.5% ropivacaine 15ml)/no block | NRS |
| Hwang BY 2020(38) | Modified radical mastectomy | 49.8 ± 8.5/ 54.7 ± 10.7 | 24/22 | Y | PVB (0.75 ropivacaine% 20ml+2 % lidocaine 10ml/no block | Postoperative morphine consumption, VAS |
| Bhuvaneswari V 2012(39) | Modified radical mastectomy | 50.7±11/49.1±7.1 | 12/12/12/12 | Y | PVB（0.25% bupivacaine with epinephrine 5mcg/ml）/PVB(0.25 bupivacaine with epinephrine 5mcg/ml with 2mcg/ml fentanyl)/PVB(0.5% bupivacaine with epinephrine 5mcg/ml)/no block | Postoperative morphine consumption, PONV, NRS, |
| Gad M 2019(40) | Modified radical mastectomy | 48.61±10.87/ 49.6±10.51 | 24/23 | Y | ESPB (0.25% levobupivacaine 20ml)/ PECS-2 block (0.25% levobupivacaine 30ml | Postoperative morphine consumption, VAS, number of postoperative rescue analgesia |
| Sotome S 2020(41) | Unilateral mastectomy with sentinel lymph node biopsy | 57±13/60±11 | 22/23 | Y | ESPB (0.375% levobupivacaine 20ml)/PVB ((0.375% levobupivacaine 20ml) | VAS, PONV |
| Sharma S 2020(42) | Modified radical mastectomy | 46.4±25.9/52.6±26.7 | 30/30 | Y | ESPB (ropivacaine 0.5% 0.4ml/kg)/no block | Postoperative morphine consumption, NRS |
| Senapathi TG 2019(43) | Modified radical mastectomy | 47.7±7.4/ 45.9±11.4 | 25/25 | Y | PECS-2 block (0.25% ropivacaine 30ml)/no block | Postoperative morphine consumption, VAS |
| Wang K 2018(44) | Modified mastectomy | 46.8±9.5/47.4±10.1 | 32/32 | Y | PECS-2 block(0.5% ropivacaine 30ml)/no block | Postoperative morphine consumption, VAS |
| Khorasanizadeh S 2020(45) | Breast cancer surgery | 18-50 | 32/32 | Y | PECS-2 block (0.25% ropivacaine 20ml)/ ESPB (0.25% ropivacaine 20ml) | VAS |
| Choi JJ 2019(46) | Breast cancer surgery | 59.4 ± 10/ 63.5 ± 8 | 20/19 | Y | PECS-2 block (0.5% ropivacaine 30ml)/no block | NRS |
| Gupta K 2017(47) | Modified radical mastectomy | 50±10.4/ 48.9±4.0 | 25/25 | Y | PVB (0.5% bupivacaine 20ml)/SPB (0.5% bupivacaine 20ml) | Postoperative morphine consumption, VAS |
| Kundra P 2013(48) | Modified radical mastectomy | 49.68±10.5/50.38±11.9 | 60/60 | N | IPB (0.5% bupivacaine 20ml)/PVB (0.5% bupivacaine 20mlg) | VAS, PONV |
| Mazzinari G 2017(49) | Oncologic breast surgery | 59.5±12.5/60.2±11.9 | 28/30 | Y | SPB (0.5% levobupivacaine 30ml)/no block | Postoperative morphine consumption, NRS |
| Mohta M 2016(50) | Breast cancer surgery | 49.9±10.6/46.6 ±10.5/ 45.3±7.4 | 15/15/15 | N | PVB (0.5% bupivacaine 0.3ml/kg)/PVB (0.5% bupivacaine 0.3ml/kg+ dexmedetomidine 1ug/kg)/ no block | NRS, postoperative morphine consumption |
| Onishi E 2018(51) | Breast cancer surgery | 54.5±12.1/55.6±13.4 | 60/62 | N | PVB (0.5% ropivacaine 30ml)/no block | NRS, postoperative time to first analgesic request, number of postoperative rescue analgesia |
| Sidiropoulou T  2007(52) | Modified radical mastectomy | 64±11/67±9 | 24/24 | N | PVB (0.5% ropivacaine 20ml)/LA infusion (0.5% ropivacaine 2ml/h for 24h postoperative | Postoperative morphine consumption, VAS, PONV |
| Kuş A 2020(53) | Breast cancer surgery | 52±6/ 51±7 | 20/20 | N | PVB (0.25% ropivacaine 20ml)/no block | Postoperative morphine consumption, NRS |
| Eldemrdash AM 2019(54) | Modified radical mastectomy with axillary clearance | 55 ± 2.9/55.1 ± 3.2 | 23/23/21 | Y | ESPB (2% articaine 20ml)/PVB block (2% articaine 20ml)/SPB block (2% articaine 20ml) | Postoperative morphine consumption, VAS, postoperative time to first analgesic request, |
| Fujii T 2019(55) | Modified radical mastectomy | 57.9 ±13.4/ 58.4 ±12.7 | 40/40 | Y | PECS-2 block (0.5% ropivacaine 30ml)/ SPB (0.5% ropivacaine 30ml) | Postoperative morphine consumption, VAS |
| Arunakul P 2010(56) | Modified Radical mastectomy | 49.4± 6.32/ 50.3±7.87 | 10/10 | N | PVB (0.5% bupivacaine 0.3ml/kg)/ no block | Postoperative morphine consumption, NRS, number of patients requiring analgesics |
| Abdallah FW 2014(57) | Breast tumor resection | 53.1±12.3/ 56.5±12.5 | 33/31 | Y | PVB (0.5% ropivacaine 15ml)/no block | Postoperative morphine consumption, PONV, NRS |
| Kamakar KM 2014(58) | Modified Radical mastectomy | 51±9/54±9 | 60/57/60 | N | PVB (2ml/kg ropivacaine)/ PVB (2mg/kg and 0.25% 0.1ml/kg ropivacaine)/no block | VRS |
| EL-sheikh SM 2016(59) | Major breast surgery | 20-80 | 20/20 | Y | PECS-2 block (2% lidocaine 30ml)/PVB (2% lidocaine 20ml) | Postoperative morphine consumption, VAS, PONV |
| Annamalai G 2017(60) | Modified radical mastectomy | 46.9±4.8/46.3±5.9 | 30/30 | Y | PECS-2 block (0.25% bupivacaine 20ml)/PVB (0.25% bupivacaine 15-20ml) | Postoperative morphine consumption, VAS |
| Kamal Abd-halim JM 2011(61) | Major breast surgery | 51±12/49±13 | 20/20 | Y | PVB (2% lidocaine 20ml)/no block | Postoperative morphine consumption, VAS |
| Pei L 2020(62) | Breast cancer surgery | 46±13/ 46±12 | 121/126 | Y | PVB (0.75% ropivacaine 5ml / no block | VAS |
| Chiu M 2014(63) | Breast cancer surgery | 54±10.8/56±10.6 | 58/60 | Y | PVB (0.5% ropivacaine 25ml) / LA infusion | Postoperative chronic pain |
| Vigneau A 2011(64) | Breast cancer surgery with axillary nodes dissection | 58±13/50 ± 11 | 22/22 | N | LA infusion (20ml of ropivacaine 7.5mg/ml)/no block | VAS, PONV |
| Albi-Feldzer A 2013(65) | Breast cancer surgery | 56±12/57±13 | 117/119 | N | LA infusion (3mg/kg 0.375% ropivacaine)/no block | VAS |
| Campbell I 2014(66) | Breast surgery | 60.6/60.4 | 45/34 | N | LA infusion (20ml 0.25% bupivacaine)/no block | VAS |
| Deng W 2020(67) | Modified radical mastectomy | 49.4±12/46.3±9.1/ 52.6±9.7/47.5±10 | 30/30/30/30 | Y | PECS-2 block (0.2% ropivacaine)/PECS-2 block (0.3% ropivacaine)/PECS-2 block (0.4 ropivacaine)/No block | Postoperative morphine consumption, NRS |
| El Karadawy S. 2020(68) | mastectomy for breast cancer | 47 ± 15.48/51 ± 9.84 | 21/22 | Y | SPB (0.25% bupivacaine 0.4ml/kg)/no block | NRS |
| Kumar S 2018(69) | Modified radical mastectomy | 47.96±7.52/49.68±8.73 | 25/25 | Y | PECS-2 block (0.25 bupivacaine 30ml)/no block | Postoperative tramadol consumption, VAS |
| Mirkheshti A 2020(70) | Breast cancer surgery | 48.67±12.63/46.36±11 | 11/11 | Y | PECS-2 block (0.25% ropivacaine 30ml)/no block | Postoperative time to first analgesic request |
| Kaya M 2013(71) | Modified radical mastectomy | 48 ±12/51 ± 10 | 30/30 | Y | IPB block (0.25% bupivacaine 30ml)/no block | PONV, number of postoperative rescue analgesia |
| Nishiyama T 2015(72) | Partial mastectomy  with lymph node dissection | 55±5/54±6 | 20/20/20 | N | PVB (0.5% ropivacaine 15ml)/PVB (0.5% ropivacaine 15ml)/no block | Postoperative time to first analgesic request |
| Abu Elyaze  d MM 2020(73) | Modified mastectomy | 54.53 ± 8.52/56.3 ±7.65 | 30/30 | Y | PECS- 2 blocks (0.25% bupivacaine 30ml) + SPB (0.25% bupivacaine 15ml)/ PECS- 2 block (0.25% bupivacaine 30ml+ 15ml normal saline) | Postoperative morphine consumption, VAS, PONV |
| Wang W 2019(74) | Modified radical mastectomy | 51.34±8.2/ 55.38 ±11.47 | 29/32 | Y | SPB+PECS-1 (0.3% ropivacaine)/no block | VAS |
| Najeeb HM 2019(75) | Radical mastectomy | 47.42±11.43/ 45.28±10.8 | 60/60 | Y | SPB+PECS-2 block (0.25% bupivacaine 40ml)/no block | Postoperative morphine consumption, VAS, PONV |
| Rahimzadeh P 2018(76) | Modified mastectomy | 49.3±7.2/ 50.2±7.8 | 30/30 | Y | SPB (0.2% bupivacaine 0.3ml/kg/no block | VAS |
| Chai B 2022(77) | Breast cancer surgery | 56.5±11.1/ 56.1±12.3 | 32/33 | Y | SPB (0.375% ropivacaine 30ml)/no block | Postoperative sufentanil consumption, VAS scores, PONV |
| Arora S2021(78) | Breast cancer surgery | 48.2±9.8/50.8 ± 9.5 | 20/20 | Y | PVB (0.4ml/kg 0.5% ropivacaine/SPB (0.4ml/kg 0.5% ropivacaine) | VAS scores |
| Albi-Feldzer A 2021(79) | Breast cancer surgery | 55±11/54±16 | 178/174 | Y | PVB (0.75% ropivacaine 0.3ml/kg)/No block (0.3ml/kg normal saline) | Incidence of chronic pain |
| Sulak MM2021(80) | Modified radical mastectomy and axillary lymph node dissection | 52.33±9.14/51.07± 10.65 | 30/30 | Y | SPB (0.25% bupivacaine 30ml)/No block (2ml normal saline | Postoperative morphine consumption, the incidence of chronic pain |
| Adallah FW2021(81) | Simple or partial mastectomy with sentinel node biopsy | 57.3±12.7/58.4±11.8 | 20/20 | Y | SPB (0.5% ropivacaine 20ml)/No block (sterile saline  Subcutaneously) | Postoperative pain scores, postoperative morphine consumption, the incidence of chronic pain |
| Makkar JK2021(82) | Unilateral modified radical mastectomy | 47.8±10.3/51.4±10.2 | 24/25 | Y | PECS-2 block (0.2% ropivacaine 30ml)/No block | Postoperative morphine consumption, PONV |
| Jiang CW2021(83) | Modified radical mastectomy | 52.1±11.5/54.73±13.6/ 56.06 ± 11.15 | 30/30/30 | Y | SPB (0.5% ropivacaine 20ml)/ESPB(0.5% ropivacaine 20ml)/RIB(0.5% ropivacaine 20ml) | Postoperative pian scores, PONV |
| Rao F2021(84) | Unilateral modified radical mastectomy | 53.6±6.2/53.4±6.6 | 34/34 | Y | PVB (0.5% ropivacaine 20ml)/no block (1 ml normal saline) | VAS, postoperative morphine consumption, PONV |
| Ciftci B2021(85) | Breast cancer surgery | 42±11.9/50±11/42±12.6 | 30/30/30 | Y | PECS-2 block (0.25% bupivacaine 30ml)/RIB (0.25% bupivacaine 30ml)/no block | VAS, postoperative morphine consumption, PONV |
| Kurien RK(86) | Breast cancer surgery | 51/47 | 30/30 | Y | PECS block (0.24% levobupivacaine)/no block | Postoperative morphine consumption |
| Xiao YK2021(87) | Breast cancer surgery | 54.8±7.2/ 55.4±7.4 | 28/28 | Y | SPB (0.33% ropivacaine 30ml)/No block | VAS, PONV |
| Ahmed MAM 2022(88) | Modified radical mastectomy | 18–65 | 15/15 | Y | PECS-2 block (0.2ml/kg bupivacaine)/PVB (0.25% bupivacaine 20ml) | postoperative morphine consumption, VAS |
| Genc C 2022(89) | Breast cancer surgery | 46.1±10.9/46.8±11.1/50.1±11.5 | 30/30/30 | Y | ESPB (0.25% bupivacaine30ml)/SPB(0.25% bupivacaine 30ml/No block | VAS, postoperative morphine consumption, postoperative time to first analgesic request |
| Qian B 2021(90) | Modified radical mastectomy | 52±5.4/51±4.8 | 90/89 | Y | SPB (0.5% ropivacaine 30ml)/No block (0.9% normal saline) | Postoperative morphine consumption, PONV, VAS, the incidence of chronic pain |
| Tang W2021(91) | Breast cancer surgery | 52.4±8.9/ 53.0±10.5 | 43/44 | Y | SPB (0.5% ropivacaine 20ml)/No block | VAS |
| Yesiltas S 2021(92) | Breast cancer surgery | 54.7±13.1/ 58.2±11.6 | 30/30 | Y | SPB+PECS-1 block (0.5ml/kg of 0.25 bupivacaine with 1% lidocaine mixture)/SPB (0.5ml/kg of 0.25 bupivacaine with 1% lidocaine mixture) | VAS, PONV, number of postoperative rescue analgesia |
| Moller JF 2007(93) | Breast cancer surgery | 57.6±11.7/57.2±12.8 | 48/41 | N | PVB (0.5% ropivacaine 30ml)/No block | Postoperative fentanyl consumption, PONV |
| Mohamed SAB 2012(94) | Modified radical mastectomy | 40.05±4.36/ 38.92±6 | 35/35 | N | LA infusion (0.5% bupivacaine 5ml)/ No block | PONV |
| Laso LF 2014(95) | Modified radical mastectomy | 54.8 ±14.7/ 57.7±15.7 | 34/39 | N | LA infusion (0.5% levobupivacaine 30ml)/No block (normal saline) | VAS, PONV |
| Fallatah S 2016(96) | Breast cancer surgery | 51.8±10.52/50.75±13.91 | 20/20 | N | PVB (0.5% bupivacaine 20ml)/No block | VAS |
| Abdelaziz Ahmed AA 2008(97) | Modified radical mastectomy | 20-65 | 30/30 | Y | PECS-2 block (28 mL 0.25% bupivacaine +2 mL normal saline) /No block | VAS |
| Lee P 2013(98) | Breast cancer surgery | 54.3±11.5/57.8 ± 14.5 | 25/26 | N | PVB (0.25 bupivacaine 10ml)/No block | VAS, the incidence of chronic pain |
| Gacio MF 2014(99) | Major breast surgery | 55.1±9.8/52.68±8.9 | 25/26 | N | PVB (0.5% ropivacaine + adrenaline 3mg/mL^−1^ with a volume of 0.3 mL/ kg^−1^ | VAS, the incidence of chronic pain |
| Xu J2016(100) | Breast cancer surgery | 46 ±11/45±12 | 30/30 | Y | PVB (0.5% ropivacaine 20ml)/No block | VAS, the incidence of chronic pain |

Notes: PECS-2 block: Pectoral nerve 2 block, PECS-1 block: Pectoral nerve 1 block, PVB: Paravertebral nerve block, ESPB: Erector spinae plane block, SPB: Serratus anterior plane block, RIB: Rhomboid intercostal block, IPB: Interpleural block LA infusion: Local anesthetic infusion. VAS: visual analogue scale, NRS: Numeric rating scale.

**Reference**

1. Al Ja'bari AR, M. El-Boghdadly, K. Albrecht, E. A randomised controlled trial of the pectoral nerves-2 (PECS-2) block for radical mastectomy. Anaesthesia. 2019;74(10):1277-81.

2. Altiparmak BKT, M. Uysal, A. I.Turan, M. Gumus Demirbilek, S. Comparison of the effects of modified pectoral nerve block and erector spinae plane block on postoperative opioid consumption and pain scores of patients after radical mastectomy surgery: A prospective, randomized, controlled trial. J Clin Anesth. 2019;54:61-5.

3. Kulhari SB, N. Bala, I. Arora, S. Singh, G. Efficacy of pectoral nerve block versus thoracic paravertebral block for postoperative analgesia after radical mastectomy: a randomized controlled trial. Br J Anaesth. 2016;117(3):382-6.

4. Altiparmak BKT, M. Uysal, A. I. Dere, O. Ugur, B. Evaluation of ultrasound-guided rhomboid intercostal nerve block for postoperative analgesia in breast cancer surgery: a prospective, randomized controlled trial. Reg Anesth Pain Med. 2020;45(4):277-82.

5. Barrington MJS, G. J. Gotmaker, R. Lim, D. Byrne, K. Quality of Recovery After Breast Surgery: A Multicenter Randomized Clinical Trial Comparing Pectoral Nerves Interfascial Plane (Pectoral Nerves II) Block With Surgical Infiltration. Anesth Analg. 2020;130(6):1559-67.

6. Yao YL, J. Hu, H. Xu, T. Chen, Y. Ultrasound-guided serratus plane block enhances pain relief and quality of recovery after breast cancer surgery: A randomised controlled trial. Eur J Anaesthesiol. 2019;36(6):436-41.

7. Hamed IG, Fawaz AA, Rabie AH, El Aziz AEAAA, Ashoor TM. Ultrasound-guided thoracic paravertebral block vs pectoral nerve block for postoperative analgesia after modified radical mastectomy. Ain-Shams Journal of Anesthesiology. 2020;12(1).

8. Kairaluoma PMB, M. S. Korpinen, A. K. Rosenberg, P. H. Pere, P. J. Single-injection paravertebral block before general anesthesia enhances analgesia after breast cancer surgery with and without associated lymph node biopsy. Anesth Analg. 2004;99(6):1837-43.

9. Aksu CK, A. Yorukoglu, H. U. Tor Kilic, C. Gurkan, Y. Analgesic effect of the bi-level injection erector spinae plane block after breast surgery: A randomized controlled trial. Agri. 2019;31(3):132-7.

10. Moustafa MA, Alabd A, S., Ahmed A M, M., Deghidy E, A. Erector spinae versus paravertebral plane blocks in modified radical mastectomy: Randomised comparative study of the technique success rate among novice anaesthesiologists. Indian J Anaesth. 2020;64(1):49-54.

11. EI Ghamary MR, Amer AF. Role of erector spinae plane block versus paravertebral block in pain control after modified radical mastectomy. A prospective randomised trial. Indian J Anaesth. 2019;63(12):1008-14.

12. Seelam S, Nair A, Christopher A, Upputuri O. Efficacy of single-shot ultrasound-guided erector spinae plane block for postoperative analgesia after mastectomy: A randomized controlled study. Saudi journal of anaesthesia. 2020;14:22.

13. Sinha C, Kumar A, kumar A, Prasad C, singh PK, Priya DP. Pectoral nerve versus erector spinae block for breast surgeries: A randomised controlled trial. Indian J Anaesth. 2019;63(8):617-22.

14. Gurkan YA, C. Kus, A. Yorukoglu, U. H. Erector spinae plane block and thoracic paravertebral block for breast surgery compared to IV-morphine: A randomized controlled trial. J Clin Anesth. 2020;59:84-8.

15. Gurkan YA, C. Kus, A. Yorukoglu, U. H. Kilic, C. T. Ultrasound guided erector spinae plane block reduces postoperative opioid consumption following breast surgery: A randomized controlled study. J Clin Anesth. 2018;50:65-8.

16. Hetta DF, Rezk KM. Pectoralis-serratus interfascial plane block vs thoracic paravertebral block for unilateral radical mastectomy with axillary evacuation. J Clin Anesth. 2016;34:91-7.

17. Pillai VS, Ramesh B, Varughese SA. Safety and efficacy of multiple site thoracic paravertebral nerve block vs. modified pectoral nerve block for postoperative analgesia after modified radical mastectomies-a randomised controlled trial. Indian J Anaesth. 2019;5(3):445-50.

18. Syal K, Chandel A. Comparison of the post-operative analgesic effect of paravertebral block, pectoral nerve block and local infiltration in patients undergoing modified radical mastectomy: A randomised double-blind trial. Indian J Anaesth. 2017;61(18):643-8.

19. Siddeshwara A, Singariya G, Kamal M, Kumari K, Seervi S, Kumar R. Comparison of efficacy of ultrasound-guided pectoral nerve block versus thoracic paravertebral block using levobupivacaine and dexamethasone for postoperative analgesia after modified radical mastectomy: A randomized controlled trial. Saudi journal of anaesthesia. 2019;12(4):325-31.

20. Wahba SS, Kamal SM. Thoracic paravertebral block versus pectoral nerve block for analgesia after breast surgery. Egyptian Journal of Anaesthesia. 2019;30(2):129-35.

21. Bashandy GMA, D. N. Pectoral nerves I and II blocks in multimodal analgesia for breast cancer surgery: a randomized clinical trial. Reg Anesth Pain Med. 2015;40(1):68-74.

22. Cros JS, P. Kaprelian, S. Desroches, J. Gagnon, C. Labrunie, A. Marin, B. Crepin, S. Nathan, N. Beaulieu, P. Pectoral I Block Does Not Improve Postoperative Analgesia After Breast Cancer Surgery: A Randomized, Double-Blind, Dual-Centered Controlled Trial. Reg Anesth Pain Med. 2018;43(6):596-604.

23. Kim DH, Kim S, Kim CS, Lee S, Lee IG, Kim HJ, et al. Efficacy of Pectoral Nerve Block Type II for Breast-Conserving Surgery and Sentinel Lymph Node Biopsy: A Prospective Randomized Controlled Study. Pain Res Manag. 2018;2018:4315931.

24. Hassn AA, Zanfaly H, Biomy T. Pre-emptive analgesia of ultrasound-guided pectoral nerve block II with dexmedetomidine–bupivacaine for controlling chronic pain after modified radical mastectomy. Research and Opinion in Anesthesia and Intensive Care. 2016;2(1):6.

25. Kamiya Y, Hasegawa M, Yoshida T, Takamatsu M, Koyama Y. Impact of pectoral nerve block on postoperative pain and quality of recovery in patients undergoing breast cancer surgery: A randomised controlled trial. Eur J Anaesthesiol. 2018;35(3):215-23.

26. O'Scanaill P, Keane S, Wall V, Flood G, Buggy DJ. Single-shot pectoral plane (PECs I and PECs II) blocks versus continuous local anaesthetic infusion analgesia or both after non-ambulatory breast-cancer surgery: a prospective, randomised, double-blind trial. Br J Anaesth. 2018;120(4):846-53.

27. Versyck B, van Geffen GJ, Chin KJ. Analgesic efficacy of the Pecs II block: a systematic review and meta-analysis. Anaesthesia. 2019;74(5):663-73.

28. Tripathy S, Mandal I, Rao PB, A. P, T. M, M. K. Opioid-free anesthesia for breast cancer surgery: A comparison of ultrasound guided paravertebral and pectoral nerve blocks. A randomized controlled trial. J Anaesthesiol Clin Pharmacol. 2019;35(4):475-80.

29. Kim DH, Kim S, Kim C, Lee S, Lee IG, Kim HJ, et al. Thoracic paravertebral block versus pectoral nerve block for analgesia after breast surgery. Pain Res Manag. 2018;2018:4315931.

30. Bakeer AHK, K. M. Abdelgalil, A. S. Ghoneim, A. A. Abouel Soud, A. H. Hassan, M. E. Modified Pectoral Nerve Block versus Serratus Block for Analgesia Following Modified Radical Mastectomy: A Randomized Controlled Trial. J Pain Res. 2020;13:1769-75.

31. Kaur U, Shamshery C, Agarwal A, Prakash N, Valiveru RC, Mishra P. Evaluation of postoperative pain in patients undergoing modified radical mastectomy with pectoralis or serratus-intercostal fascial plane blocks. Korean J Anesthesiol. 2020;73(5):425-33.

32. He W, Wu Z, Zu L, Sun H, Yang X. Application of erector spinae plane block guided by ultrasound for postoperative analgesia in breast cancer surgery: A randomized controlled trial. Cancer Commun (Lond). 2020;40(2-3):122-5.

33. Thomas M, Philip FA, Mathew A, P., Jagathnath K, K.M. Erector spinae versus paravertebral plane blocks in modified radical mastectomy: Randomised comparative study of the technique success rate among novice anaesthesiologists

J Anaesthesiol Clin Pharmacol. 2018;34(3):318-23.

34. Bansal P, Saxena KN, Taneja B, Sareen B. A comparative randomized study of paravertebral block versus wound infiltration of bupivacaine in modified radical mastectomy. J Anaesthesiol Clin Pharmacol. 2012;28(1):76-80.

35. Singh S, Kumar G, Akhileshwar. Ultrasound-guided erector spinae plane block for postoperative analgesia in modified radical mastectomy: A randomised control study. Indian J Anaesth. 2019;63(3):200-4.

36. Ilfeld BM, Madison SJ, Suresh PJ, Sandhu NS, Kormylo NJ, Malhotra N, et al. Treatment of postmastectomy pain with ambulatory continuous paravertebral nerve blocks: a randomized, triple-masked, placebo-controlled study. Reg Anesth Pain Med. 2014;39(2):89-96.

37. Qian B, Fu S, Yao Y, Lin D, Huang L. Preoperative ultrasound-guided multilevel paravertebral blocks reduce the incidence of postmastectomy chronic pain: a double-blind, placebo-controlled randomized trial. J Pain Res. 2019;12:597-603.

38. Hwang BY, Kim E, Kwon JY, Lee JY, Lee D, Park EJ, et al. The analgesic efficacy of a single injection of ultrasound-guided retrolaminar paravertebral block for breast surgery: a prospective, randomized, double-blinded study. Korean J Pain. 2020;33(4):378-85.

39. Bhuvaneswari V, Wig J, Mathew PJ, Singh G. Post-operative pain and analgesic requirements after paravertebral block for mastectomy: A randomized controlled trial of different concentrations of bupivacaine and fentanyl. Indian J Anaesth. 2012;56(1):34-9.

40. Gad M, Abdelwahab K, Abdallah A, Abdelkhalek M, Abdelaziz M. Ultrasound-Guided Erector Spinae Plane Block Compared to Modified Pectoral Plane Block for Modified Radical Mastectomy Operations. Anesth Essays Res. 2019;13(2):334-9.

41. Sotome S, Sawada A, Wada A, Shima H, Kutomi G, Yamakage M. Erector spinae plane block versus retrolaminar block for postoperative analgesia after breast surgery: a randomized controlled trial. Journal of Anesthesia. 2020.

42. Sharma S, Arora S, Jafra A, Singh G. Efficacy of erector spinae plane block for postoperative analgesia in total mastectomy and axillary clearance: A randomized controlled trial. Saudi J Anaesth. 2020;14(2):186-91.

43. Senapathi TGA, Widnyana IMG, Aribawa I, Jaya A, Junaedi IMD. Combined ultrasound-guided Pecs II block and general anesthesia are effective for reducing pain from modified radical mastectomy. J Pain Res. 2019;12:1353-8.

44. Wang K, Zhang X, Zhang T, Yue H, Sun S, Zhao H, et al. The Efficacy of Ultrasound-guided Type II Pectoral Nerve Blocks in Perioperative Pain Management for Immediate Reconstruction After Modified Radical Mastectomy: A Prospective, Randomized Study. Clin J Pain. 2018;34(3):231-6.

45. Khorasanizadeh S, Arabzadeh B, Teymourian H, Mohseni GR. Pectoral Nerve Block and Erector Spinae Plane Block and Post-Breast Surgery Complications. International Journal of Cancer Management. 2020;13(3).

46. Choi JJJ, Y. Y. Kim, S. H. Jung, W. S. Lee, D. Kim, K. Y. Kwak, H. J. Remifentanil-Sparing Effect of Pectoral Nerve Block Type II in Breast Surgery under Surgical Pleth Index-Guided Analgesia during Total Intravenous Anesthesia. J Clin Med. 2019;8(8).

47. Gupta KS, K. Girdhar, K. K. Chan, V. Analgesic efficacy of ultrasound-guided paravertebral block versus serratus plane block for modified radical mastectomy: A randomised, controlled trial. Indian J Anaesth. 2017;61(5):381-6.

48. Kundra P, Varadharajan R, Yuvaraj K, Vinayagam S. Comparison of paravertebral and interpleural block in patients undergoing modified radical mastectomy. J Anaesthesiol Clin Pharmacol. 2013;29(4):459-64.

49. Mazzinari G, Rovira L, Casasempere A, Ortega J, Cort L, Esparza-Minana JM, et al. Interfascial block at the serratus muscle plane versus conventional analgesia in breast surgery: a randomized controlled trial. Reg Anesth Pain Med. 2019;44(1):52-8.

50. Mohta M, Kalra B, Sethi AK, Kaur N. Efficacy of dexmedetomidine as an adjuvant in paravertebral block in breast cancer surgery. J Anesth. 2016;30(2):252-60.

51. Onishi E, Murakami M, Nishino R, Ohba R, Yamauchi M. Analgesic Effect of Double-Level Retrolaminar Paravertebral Block for Breast Cancer Surgery in the Early Postoperative Period: A Placebo-Controlled, Randomized Clinical Trial. Tohoku J Exp Med. 2018;245(3):179-85.

52. Sidiropoulou T, Buonomo O, Fabbi E, Silvi MB, Kostopanagiotou G, Sabato AF, et al. A prospective comparison of continuous wound infiltration with ropivacaine versus single-injection paravertebral block after modified radical mastectomy. Anesth Analg. 2008;106(3):997-1001, table of contents.

53. Kuş A, Yörükoğlu UH, Aksu C, Çınar S, Cantürk NZ, Gürkan Y. The effect of thoracic paravertebral block on seroma reduction in breast surgery – a randomized controlled trial. Brazilian Journal of Anesthesiology (English Edition). 2020;70(3):215-9.

54. Eldemrdash AMA, El-Sayed Mohamed. By Ultrasonic-Guided Erector Spinae Block, Thoracic Paravertebral Block versus Serratus Anterior Plane Block by Articaine with Adrenaline during Breast Surgery with General Anesthesia: A Comparative Study of Analgesic Effect Post-Operatively: Double Blind Randomized, Controlled Trial. Open Journal of Anesthesiology. 2019;09(04):68-82.

55. Fujii TS, Y. Akane, A. Aoki, W. Sekiguchi, A. Takahashi, K. Matsui, S. Nishiwaki, K. A randomised controlled trial of pectoral nerve-2 (PECS 2) block vs. serratus plane block for chronic pain after mastectomy. Anaesthesia. 2019;74(12):1558-62.

56. Arunakul P, Ruksa A. General anesthesia with thoracic paravertebral block for modified radical mastectomy. J Med Assoc Thai. 2010;93 Suppl 7:S149-53.

57. Abdallah FW, Morgan PJ, Cil T, McNaught A, Escallon JM, Semple JL, et al. Ultrasound-guided multilevel paravertebral blocks and total intravenous anesthesia improve the quality of recovery after ambulatory breast tumor resection. Anesthesiology. 2014;120(3):703-13.

58. Karmakar MK, Samy W, Li JW, Lee A, Chan WC, Chen PP, et al. Thoracic paravertebral block and its effects on chronic pain and health-related quality of life after modified radical mastectomy. Reg Anesth Pain Med. 2014;39(4):289-98.

59. EL-sheikh SM, Fouad A, Bashandy GN, AL-azzb MA, Gamal RM. Ultrasound Guided Modified Pectoral Nerves Block versus Thoracic Paravertebral Block for Perioperative Analgesia in Major Breast Surgery. MED J Cairo Univ. 2016;84(3):189-95.

60. Annamalai G, Kumar Durairaj A, Kailasam R K. Pectoral Nerve Block Versus Thoracic Paravertebral Block– Comparison of Analgesic Efficacy for Postoperative Pain Relief in Modified Radical Mastectomy Surgeries. Journal of Evolution of Medical and Dental Sciences. 2017;6(60):4412-6.

61. Kamal Abdel-halim JM. Continuous thoracic paravertebral block: An adjunct to general anaesthesia in major breast surgery. Egyptian Journal of Anaesthesia. 2019;27(2):83-7.

62. Pei L, Zhou Y, Tan G, Mao F, Yang D, Guan J, et al. Ultrasound-Assisted Thoracic Paravertebral Block Reduces Intraoperative Opioid Requirement and Improves Analgesia after Breast Cancer Surgery: A Randomized, Controlled, Single-Center Trial. PLoS One. 2015;10(11):e0142249.

63. Chiu MB, G. L. Lui, A. Watters, J. M. Taljaard, M. Nathan, H. J. Reducing persistent postoperative pain and disability 1 year after breast cancer surgery: a randomized, controlled trial comparing thoracic paravertebral block to local anesthetic infiltration. Ann Surg Oncol. 2014;21(3):795-801.

64. Vigneau A, Salengro A, Berger J, Rouzier R, Barranger E, Marret E, et al. A double blind randomized trial of wound infiltration with ropivacaine after breast cancer surgery with axillary nodes dissection. Bmc Anesthesiology. 2011;11(23):1471-2253.

65. Albi-Feldzer AM-F, E. E. Hamouda, S. Motamed, C. Dubois, P. Y. Jouanneau, L. Jayr, C. A double-blind randomized trial of wound and intercostal space infiltration with ropivacaine during breast cancer surgery: effects on chronic postoperative pain. Anesthesiology. 2013;118(2):318-26.

66. Campbell IC, S. Creighton, J. French, R. Banerjee, S. Kerr, E. Shirley, R. To infiltrate or not? Acute effects of local anaesthetic in breast surgery. ANZ J Surg. 2015;85(5):353-7.

67. Deng WF, D. He, L. Evaluation of Pectoral Nerve Block in Modified Radical Mastectomy: Comparison of Three Concentrations of Ropivacaine. Clin Interv Aging. 2020;15:937-44.

68. ElKaradawy S. A. EMA, Ahmed Y. , Khaled M. A. T. Ultrasound-guided serratus plane block with continuous postoperative drug delivery system for acute nociceptive and neuropathic pain after mastectomy. Egyptian Journal of Anaesthesia. 2020;36(1):97-104.

69. Kumar S, Goel D, Sharma SK, Ahmad S, Dwivedi P, Deo N, et al. A randomised controlled study of the post-operative analgesic efficacy of ultrasound-guided pectoral nerve block in the first 24 h after modified radical mastectomy. Indian J Anaesth. 2018;62(6):436-42.

70. Mirkheshti A, Memary E, Sayyadi S, Samsami M, Motevalli SH. The Effect of Pectoral Nerves Blocks on Narcotic Consumption and Pain Intensity in the Patients Undergoing Breast Cancer Surgery. International Journal of Cancer Management. 2020;13(5):e98879.

71. Kaya M, Oguz G, Senel G, Kadiogullari N. Postoperative analgesia after modified radical mastectomy: the efficacy of interscalene brachial plexus block. J Anesth. 2013;27(6):862-7.

72. Nishiyama T. A randomized controlled study of the effects of single or multilevel paravertebral block on postoperative analgesia in partial mastectomy with lymph node dissection. Anestheis, Pain & Intensive Care. 2015;19(4):463-7.

73. Abu Elyazed MMA, M. S. Mostafa, S. F. The Analgesic Efficacy of Pecto-Intercostal Fascial Block Combined with Pectoral Nerve Block in Modified Radical Mastectomy: A Prospective Randomized Trial. Pain Physician. 2020;23(5):485-93.

74. Wang W, Song W, Yang C, Sun Q, Chen H, Zhang L, et al. Ultrasound-Guided Pectoral Nerve Block I and Serratus-Intercostal Plane Block Alleviate Postoperative Pain in Patients Undergoing Modified Radical Mastectomy. Pain Physician. 2019;22(4):E315-E23.

75. Najeeb HN, Mehdi SR, Siddiqui AM, Batool SK. Pectoral Nerves I, II and Serratus Plane Blocks in Multimodal Analgesia for Mastectomy: A Randomised Clinical Trial. J Coll Physicians Surg Pak. 2019;29(10):910-4.

76. Rahimzadeh P, Imani F, Faiz SHR, Boroujeni BV. Impact of the Ultrasound-Guided Serratus Anterior Plane Block on Post-Mastectomy Pain: A Randomised Clinical Study. Turk J Anaesthesiol Reanim. 2018;46(5):388-92.

77. Chai B, Yu H, Qian Y, Chen X, Zhu Z, Du J, et al. Comparison of Postoperative Pain in 70 Women with Breast Cancer Following General Anesthesia for Mastectomy with and without Serratus Anterior Plane Nerve Block. Med Sci Monit. 2022;28:e934064.

78. Arora S, Ovung R, Bharti N, Yaddanapudi S, Singh G. Efficacy of serratus anterior plane block versus thoracic paravertebral block for postoperative analgesia after breast cancer surgery: a randomized trial. Braz J Anesthesiol. 2021.

79. Albi-Feldzer A, Dureau S, Ghimouz A, Raft J, Soubirou JL, Gayraud G, et al. Preoperative Paravertebral Block and Chronic Pain after Breast Cancer Surgery: A Double-blind Randomized Trial. Anesthesiology. 2021;135(6):1091-103.

80. Sulak M, Ahiskalioglu A, Yayik A, Karadeniz E, Celik M, Demir U, et al. The effect of ultrasound-guided serratus plane block on the quality of life in patients undergoing modified radical mastectomy and axillary lymph node dissection: a randomized controlled study. Anaesthesiol Intensive Ther. 2022;54(1):48-55.

81. Abdallah FW, Patel V, Madjdpour C, Cil T, Brull R. Quality of recovery scores in deep serratus anterior plane block vs. sham block in ambulatory breast cancer surgery: a randomised controlled trial. Anaesthesia. 2021;76(9):1190-7.

82. Makkar JK, Dahiya D, Jain D, Kuber A, Kajal K, Singh NP. Effect of balanced anaesthesia with and without modified pectoralis nerve block on postoperative analgesia after breast surgeries: A randomised controlled trial. Indian J Anaesth. 2021;65(Suppl 3):S110-S4.

83. Jiang CW, Liu F, Zhou Q, Deng W. Comparison of rhomboid intercostal nerve block, erector spinae plane block and serratus plane block on analgesia for modified radical mastectomy: A prospective randomised controlled trial. Int J Clin Pract. 2021;75(10):e14539.

84. Rao F, Wang Z, Chen X, Liu L, Qian B, Guo Y. Ultrasound-Guided Thoracic Paravertebral Block Enhances the Quality of Recovery After Modified Radical Mastectomy: A Randomized Controlled Trial. J Pain Res. 2021;14:2563-70.

85. Ciftci B, Ekinci M, Basim P, Celik EC, Tukac IC, Zenciroglu M, et al. Comparison of Ultrasound-Guided Type-II Pectoral Nerve Block and Rhomboid Intercostal Block for Pain Management Following Breast Cancer Surgery: A Randomized, Controlled Trial. Pain Pract. 2021;21(6):638-45.

86. Kurien RK, Salins SR, Jacob PM, Thomas K. Utility of Pecs Block for Perioperative Opioid-Sparing Analgesia in Cancer-Related Breast Surgery: A Randomized Controlled Trial. Indian J Surg Oncol. 2021;12(4):713-21.

87. Xiao YK, She SZ, Xu LX, Zheng B. Serratus Anterior Plane Block Combined with General Analgesia and Patient-Controlled Serratus Anterior Plane Block in Patients with Breast Cancer: A Randomized Control Trial. Adv Ther. 2021;38(6):3444-54.

88. Ahmed MAM, Elhenawy AME, Awad HGS, Ali AHH, Elfawy DMA. Comparative study between intraoperative and postoperative analgesic effect of ultrasound-guided thoracic paravertebral block versus pectoral nerve block in patients undergoing modified radical mastectomy: a randomized controlled trial. Ain Shams Journal of Anesthesiology. 2022;14(1).

89. Genc C, Kaya C, Bilgin S, Dost B, Ustun YB, Koksal E. Pectoserratus plane block versus erector spinae plane block for postoperative opioid consumption and acute and chronic pain after breast cancer surgery: A randomized controlled trial. J Clin Anesth. 2022;79:110691.

90. Qian B, Huang S, Liao X, Wu J, Lin Q, Lin Y. Serratus anterior plane block reduces the prevalence of chronic postsurgical pain after modified radical mastectomy: A randomized controlled trial. J Clin Anesth. 2021;74:110410.

91. Tang W, Luo G, Lu Y, Chen C, Liu H, Li Y. Application of a new serratus anterior plane block in modified radical mastectomy under ultrasound guidance: A prospective, randomized controlled trial. J Clin Anesth. 2021;74:110377.

92. Yesiltas S, Turkoz A, Calim M, Yilmaz S, Esen A, Daskaya H, et al. Comparison of serratus plane block alone and in combination with pectoral type 1 block for breast cancer surgery: a randomized controlled study. Hippokratia. 2021;25(1):8-14.

93. Moller JF, Nikolajsen L, Rodt SA, Ronning H, Carlsson PS. Thoracic paravertebral block for breast cancer surgery: a randomized double-blind study. Anesth Analg. 2007;105(6):1848-51, table of contents.

94. Mohamed SA, Abdel-Ghaffar HS. Effect of the addition of clonidine to locally administered bupivacaine on acute and chronic postmastectomy pain. J Clin Anesth. 2013;25(1):20-7.

95. Ferreira Laso L, Lopez-Picado A, Lamata L, Ceballos Garcia M, Ibanez Lopez C, Pipaon Ruilope L, et al. Postoperative analgesia by infusion of local anesthetic into the surgical wound after modified radical mastectomy: a randomized clinical trial. Plast Reconstr Surg. 2014;134(6):862e-70e.

96. Fallatah S, Mousa WF. Multiple levels paravertebral block versus morphine patient-controlled analgesia for postoperative analgesia following breast cancer surgery with unilateral lumpectomy, and axillary lymph nodes dissection. Saudi J Anaesth. 2016;10(1):13-7.

97. Abdelaziz Ahmed AA. Efficacy of Pectoral Nerve Block using Bupivacaine with or without Magnesium Sulfate. Anesth Essays Res. 2018;12(2):440-5.

98. P. Lee NM, C. Dunlop, M. Palanisamy, G. Shorten. A comparison of the effects of two analgesic regimens on the development of persistent post-surgical pain (PPSP) after breast surgery. J Rom Anest Terap Int. 2013;20(2):83-93.

99. Gacio MF, Lousame AM, Pereira S, Castro C, Santos J. Paravertebral block for management of acute postoperative pain and intercostobrachial neuralgia in major breast surgery. Braz J Anesthesiol. 2016;66(5):475-84.

100. Xu J, Zhou Y, Wang Y, Zhang H. Clinical Evaluation of Ultrasound-Guided Thoracic Paravertebral Block (TPVB) Effect on Postoperative Analgesia in Patients with Breast Cancer after Radical Mastectomy. Primary Health Care Open Access. 2016;06(04).

## Appendix 3

## Risk of bias assessment


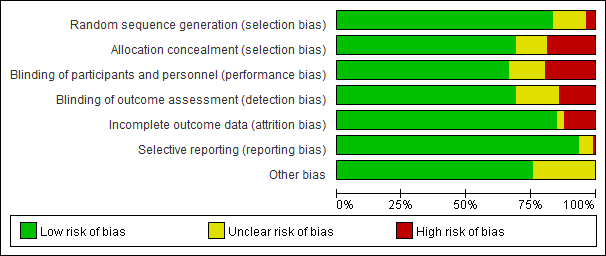


**Figure 1. Risk of bias summary: it is a summary table of review authors’ judgments for each risk of bias entry for each study**

**Table 1. Risk of bias assessment using Cochrane tool**

| **Author** | **Random Sequence generation** | **Allocation concealment** | | **Blinding of participants &personnel** | **Blinding of outcome assessment** | **Incomplete outcome data** | | **Selective reporting** | **Other bias** | **Total** |
| --- | --- | --- | --- | --- | --- | --- | --- | --- | --- | --- |
| **Ja’bari AA 2019** | **Low** | **Low** | | **Low** | **Low** | **Low** | | **Low** | **Low** | **Low** |
| **Altıparmak B 2019** | **Low** | **Low** | | **Low** | **Low** | **Low** | | **Low** | **low** | **Low** |
| **Kulhari S 2016** | **Low** | **Low** | | **Low** | **Low** | **Low** | | **Low** | **Low** | **Low** |
| **Altiparmak B 2020** | **Low** | **Low** | | **Low** | **Low** | **Low** | | **Low** | **Low** | **Low** |
| **Barrington MJ 2019** | **Low** | **Low** | | **Low** | **Low** | **Low** | | **Low** | **low** | **Low** |
| **Yao Y 2019** | **Low** | **Low** | | **Low** | **Low** | **Low** | | **Unclear** | **unclear** | **Moderate** |
| **Hamed IG 2020** | **Low** | **Unclear** | | **Unclear** | **Unclear** | **Low** | | **Low** | **Low** | **Moderate** |
| **Boughey JC 2009** | **Unclear** | **Unclear** | | **Low** | **Low** | **Low** | | **Low** | **low** | **Moderate** |
| **Kairaluoma PM 2004** | **Low** | **Unclear** | | **Low** | **Low** | **Low** | | **Low** | **low** | **Moderate** |
| **Aksu C 2019** | **Low** | **Low** | | **Low** | **Low** | **Low** | | **Low** | **low** | **Low** |
| **Moustafa MA 2020** | **Low** | **Low** | | **Low** | **Low** | **Low** | | **Low** | **low** | **Moderate** |
| **EI Ghamry MR2019** | **Low** | **Low** | | **Low** | **Low** | **Low** | | **Low** | **low** | **Low** |
| **Seealam S 2020** | **Unclear** | **Low** | | **Low** | **Low** | **Low** | | **Low** | **low** | **Moderate** |
| **Sinha C 2019** | **Low** | **Low** | | **Low** | **Low** | **Low** | | **Low** | **Low** | **Low** |
| **Gürkan Y 2020** | **Low** | **Low** | | **Low** | **Low** | **Low** | | **Low** | **Low** | **Low** |
| **Gürkan Y 2018** | **Low** | **Low** | | **Low** | **Low** | **Low** | | **Low** | **Low** | **Low** |
| **Hetta DF 2016** | **Low** | **Low** | | **Low** | **Low** | **Low** | | **Low** | **Low** | **Low** |
| **Pill ai VS 2018** | **Low** | **Low** | | **High** | **Low** | **Low** | | **High** | **Low** | **High** |
| **Syal K 2017** | **Low** | **Low** | | **Low** | **Low** | **Low** | | **Low** | **Low** | **Low** |
| **Siddeshwara A 2019** | **Low** | **Low** | | **Low** | **Low** | **Low** | | **Low** | **low** | **Low** |
| **Wahba SS 2019** | **unclear** | **High** | | **High** | **High** | **High** | | **Low** | **unclear** | **High** |
| **Bashandy GMN 2015** | **Low** | **Unclear** | | **Unclear** | **Unclear** | **Low** | | **Low** | **unclear** | **High** |
| **CrosJ 2018** | **Low** | **Low** | | **Low** | **Low** | **Low** | | **Low** | **Low** | **Low** |
| **MN 2018** | **Low** | **Low** | | **Unclear** | **Low** | **Low** | | **Low** | **Low** | **Moderate** |
| **Hassn AMA 2016** | **Unclear** | **Unclear** | | **Low** | **Low** | **Low** | | **Low** | **Low** | **High** |
| **Kamiya Y 2017** | **Low** | **Low** | | **Low** | **Low** | **Low** | | **Low** | **Low** | **Low** |
| **O’Scanaill 2018** | **Low** | **Low** | | **Low** | **Low** | **Low** | | **Low** | **Low** | **Low** |
| **Versyck B 2017** | **Low** | **Low** | | **Low** | **Low** | **Low** | | **Low** | **Low** | **Low** |
| **Tripathy S 2019** | **Low** | **Low** | | **Low** | **Low** | **Low** | | **Low** | **Low** | **Low** |
| **Kim DH 2018** | **Low** | **Low** | | **Unclear** | **Unclear** | **Low** | | **Low** | **low** | **High** |
| **Bakeer AH 2020** | **Low** | **Low** | | **Low** | **Low** | **Low** | | **Low** | **Low** | **Low** |
| **Kaur U 2020** | **Low** | **Unclear** | | **Unclear** | **Unclear** | **Low** | | **Low** | **unclear** | **High** |
| **He W 2020** | **Low** | **Low** | | **Low** | **Low** | **Unclear** | | **Unclear** | **low** | **Low** |
| **Thomas M 2018** | **Low** | **Low** | | **Low** | **Low** | **Low** | | **Low** | **Low** | **Low** |
| **Bansal P 2012** | **Low** | **Unclear** | | **Unclear** | **Unclear** | **Low** | | **Low** | **Low** | **High** |
| **Singh S 2019** | **Low** | **Low** | | **Low** | **Low** | **Low** | | **Low** | **Low** | **Low** |
| **IIfeld BM 2015** | **Low** | **Low** | | **Low** | **Low** | **Low** | | **Low** | **Low** | **Low** |
| **Qian B 2019** | **Low** | **Low** | | **Low** | **Low** | **Low** | | **Low** | **Low** | **Low** |
| **Hwang BY 2020** | **Low** | **Low** | | **Low** | **Low** | **Low** | | **Low** | **Low** | **Low** |
| **Bhuvaneswari V 2012** | **Low** | **Low** | | **Low** | **Low** | **Low** | | **Low** | **Low** | **Low** |
| **Gad M 2019gaiguo** | **Low** | **High** | | **Unclear** | **Unclear** | **Low** | | **Low** | **unclear** | **High** |
| **Sotome S 2020** | **Low** | **Low** | | **Low** | **Low** | **Low** | | **Low** | **Low** | **Low** |
| **Sharma S 2020** | **Low** | **Low** | | **Unclear** | **Unclear** | **Low** | | **Low** | **Low** | **Moderate** |
| **Senapathi TG 2019** | **Low** | **Low** | | **Low** | **Unclear** | **Low** | | **Low** | **Low** | **Low** |
| **Wang K 2018** | **Low** | **High** | | **Low** | **Low** | **Low** | | **Low** | **Low** | **High** |
| **Khorasanizadeh S 2020** | **Unclear** | **Unclear** | | **High** | **Low** | **Low** | | **Low** | **unclear** | **High** |
| **Choi JJ 2019gaiguo** | **Low** | **High** | | **Low** | **Low** | **Low** | | **Low** | **unclear** | **High** |
| **Gupta K 2017** | **Low** | **Low** | | **Low** | **Low** | **Low** | | **Low** | **Low** | **Low** |
| **Kundra P 2013** | **Low** | **High** | | **High** | **High** | **Low** | | **Low** | **Unclear** | **High** |
| **Mazzinari G 2017** | **Low** | **Low** | | **Unclear** | **Unclear** | **Unclear** | | **Low** | **Low** | **High** |
| **Mohta M 2016** | **Low** | **Low** | | **Low** | **Low** | **Low** | | **Low** | **Low** | **Low** |
| **Onishi E 2018** | **Low** | **Unclear** | | **Low** | **Low** | **Low** | | **Low** | **Low** | **Moderate** |
| **Sidiropoulou T 2007** | **Low** | **Low** | | **High** | **Unclear** | **Low** | | **Low** | **Low** | **High** |
| **Kuş A 2020** | **Low** | **High** | | **High** | **Low** | **Low** | | **Low** | **Low** | **High** |
| **Eldemrdash AM 2019** | **Low** | **Low** | | **Low** | **Low** | **Low** | | **Low** | **Low** | **Low** |
| **Fujii T 2019** | **Low** | **Low** | | **High** | **High** | **High** | | **Low** | **Low** | **High** |
| **Arunakul P 2010** | **Low** | **Low** | | **Low** | **Low** | **Low** | | **Low** | **Low** | **Low** |
| **Abdallah FW 2013** | **Low** | **Low** | | **Low** | **Low** | **Low** | | **Low** | **Low** | **Low** |
| **Kamakar KM 2014** | **Low** | **Low** | | **Low** | **Low** | **Low** | | **Low** | **Low** | **Low** |
| **EL-sheikh SM 2016** | **High** | **High** | | **High** | **High** | **High** | | **Low** | **unclear** | **High** |
| **Annamalai G 2017** | **Low** | **Low** | | **Low** | **Low** | **Low** | | **Low** | **Low** | **Low** |
| **Kamal Abd-halim JM 2011** | **High** | **High** | | **High** | **High** | **Low** | | **Loghw** | **Unclear** | **High** |
| **Pei L 2015** | **Low** | **High** | | **Low** | **Low** | **Low** | | **Low** | **Low** | **High** |
| **Chiu M 2014** | **Low** | **Low** | | **Low** | **Low** | **Low** | | **Low** | **Low** | **Low** |
| **Vigneau A 2011** | **Low** | **Low** | | **Low** | **Low** | **Low** | | **Low** | **Low** | **Low** |
| **Albi-Feldzer A 2013** | **Low** | **Low** | | **Low** | **Low** | **Low** | | **Low** | **Low** | **low** |
| **Campbell I 2014** | **Low** | **Low** | | **High** | **Low** | **Low** | | **Low** | **Low** | **Low** |
| **Deng W 2020** | **Low** | **Low** | | **High** | **Unclear** | **Low** | | **Low** | **Unclear** | **High** |
| **ElKaradawy S 2020** | **Unclear** | **High** | | **High** | **High** | **Low** | | **Low** | **Unclear** | **High** |
| **Kumar S 2018** | **Unclear** | **High** | | **Unclear** | **High** | **Low** | | **Low** | **Unclear** | **High** |
| **Mirkheshti A 2020** | **Unclear** | **High** | | **High** | **High** | **High** | | **Low** | **Unclear** | **High** |
| **kaya M 2013** | **Low** | **Low** | | **High** | **High** | **Low** | | **Low** | **Unclear** | **High** |
| **Nishiyama T 2015** | **Unclear** | **Unclear** | | **High** | **High** | **Low** | | **Low** | **Unclear** | **High** |
| **Wang W 2019** | **Low** | **Low** | | **Low** | **Low** | **Low** | | **Low** | **Low** | **Low** |
| **Najeeb HM 2019** | **Unclear** | **High** | | **High** | **Low** | **Low** | | **Low** | **Low** | **Moderate** |
| **Rahimzadeh P 2018** | **Low** | **Low** | | **Low** | **Low** | **Low** | | **Low** | **Low** | **Low** |
| **Chai B 2022** | **Low** | **Low** | | **Low** | **Low** | **Low** | | **Low** | **Low** | **Low** |
| **Arora S2021** | **Low** | **Low** | | **High** | **Low** | **Low** | | **Low** | **Low** | **Low** |
| **Albi-Feldzer A 2021** | **Low** | **Low** | | **Low** | **Low** | **Low** | | **Low** | **Low** | **Low** |
| **Sulak MM2021** | **Low** | **Low** | | **Low** | **Low** | **Low** | | **Low** | **Low** | **low** |
| **Adallah FW2021** | **Low** | **Low** | | **Low** | **Low** | **Low** | | **Low** | **Low** | **low** |
| **Makkar JK2021** | **Low** | **Low** | | **Low** | **Low** | **Low** | | **Low** | **Low** | **low** |
| **Jiang CW2021** | **Low** | **High** | | **Low** | **Low** | **Low** | | **Low** | **Unclear** | **High** |
| **Rao F2021** | **Low** | **Low** | | **Low** | **Low** | **Low** | | **Low** | **Low** | **low** |
| **Ciftci B2021** | **Low** | **High** | | **High** | **High** | **Low** | | **Low** | **Unclear** | **High** |
| **Kurien RK** | **Unclear** | **High** | | **High** | **High** | **Low** | | **Low** | **Unclear** | **High** |
| **Xiao YK2021** | **Unclear** | **High** | | **High** | **High** | **Low** | | **low** | **Unclear** | **High** |
| **Ahmed MAM 2022** | **Low** | **High** | | **High** | **High** | **Low** | | **Low** | **Unclear** | **high** |
| **Genc C 2022** | **Unclear** | **Low** | | **Low** | **Low** | **Low** | | **Low** | **Unclear** | **Moderate** |
| **Qian B 2021** | **Low** | **Low** | | **Low** | **Low** | **Low** | | **Low** | **Low** | **low** |
| **Tang W2021** | **Low** | **Low** | | **Low** | **Low** | **Low** | | **Low** | **Low** | **low** |
| **Yesiltas S 2021** | **High** | **High** | | **Unclear** | **Unclear** | **Unclear** | | **Low** | **Low** | **Moderate** |
| **Moller JF 2007** | **Low** | **Low** | | **Low** | **Low** | **Low** | | **Low** | **Low** | **low** |
| **Mohamed SAB 2012** | **Low** | **Low** | | **Low** | **Low** | **Low** | | **Low** | **Low** | **low** |
| **Laso LF 2014** | **Low** | **Low** | | **Low** | **Low** | **Low** | | **Low** | **Low** | **low** |
| **Fallatah S 2016** | **Low** | **Low** | | **Low** | **Unclear** | **High** | | **Low** | **Low** | **High** |
| **Abdelaziz Ahmed AA 2008** | **Low** | **High** | | **High** | **High** | **Low** | | **Low** | **Low** | **High** |
| **Lee P 2013** | **Low** | **High** | | **High** | **High** | **High** | | **Low** | **Unclear** | **High** |
| **Gacio MF 2014** | **Unclear** | **Unclear** | | **Unclear** | **Unclear** | **Unclear** | | **Low** | **Unclear** | **High** |
| **Xu J2016** | **High** | **High** | | **high** | **High** | **High** | | **Low** | **Unclear** | **High** |
|  | **Low** | | **Unclear/Moderate** | | | | **High** | | | |

## Appendix 4

## Direct pair-wise meta-analysis of different nerve blocks on breast surgery

Notes: UL: upper confidence limit of 95% confidence interval; LL: lower confidence limit of 95% confidence interval; MD: mean difference; PECS-2 block: Pectoral nerve 2 block, PECS-1 block: Pectoral nerve 1 block, PVB: Paravertebral nerve block, ESPB: Erector spinae plane block, SPB: Serratus anterior plane block, RIB: Rhomboid intercostal block, IPB: Interpleural block LA infusion: Local anesthetic infusion, N: None.

| **Interventions** | | **No. of trials** | **No. of patients** | **MD** | **LL** | **UL** | ***P*** | ***I*^2^(%)** |
| --- | --- | --- | --- | --- | --- | --- | --- | --- |
| ESPB VS | PVB | 1 | N | N | N | N | N | N |
|  | No block | 3 | 150 | -3.96 | -6.87 | -0.04 | <0.05 | 0 |
|  | RIB | 1 | N | N | N | N | N | N |
|  | PECS-2 block | 2 | 124 | 0.5 | -0.15 | 1.14 | 0.13 | 69.6 |
| LA infusion VS | No block | 5 | 485 | -1.14 | -1.93 | -0.35 | <0.05 | 92.9 |
| PECS-1 block VS | No block | 1 | N | N | N | N | N | N |
| PECS-2 block VS | LA infusion | 3 | 175 | -0.71 | -1.62 | 0.2 | 0.13 | 89.3 |
|  | No block | 12 | 764 | -2.84 | -3.9 | -1.78 | <0.05 | 96.5 |
|  | RIB | 1 | N | N | N | N | N | N |
|  | SPB | 1 | N | N | N | N | N | N |
| PVB VS | LA infusion | 2 | 84 | -0.06 | -2.78 | 2.66 | 0.97 | 78.2 |
|  | No block | 10 | 823 | -1.21 | -1.94 | -0.48 | <0.05 | 95.6 |
|  | PECS-2 block | 6 | 277 | 0.8 | 0.06 | 1.54 | 0.03 | 88.5 |
|  | PVB | 1 | N | N | N | N | N | N |
| SPB VS | No block | 6 | 527 | -0.93 | -1.72 | -0.14 | 0.02 | 92.4 |
| RIB VS | No block | 1 | N | N | N | N | N | N |
|  | ESPB |  | N | N | N | N | N | N |
| SPB+PECS-1 block VS | SPB | 1 | N | N | N | N | N | N |
| SPB+PECS-2 block VS | No block | 1 | N | N | N | N | N | N |

**Table1. Postoperative PACU pain scores**

**Table 2. Postoperative 24-hour pain scores**

| **Interventions** |  | **No. of trials** | **No. of patients** | **MD** | **LL** | **UL** | ***P*** | ***I*^2^(%)** |
| --- | --- | --- | --- | --- | --- | --- | --- | --- |
| ESPB VS | No block | 6 | 300 | -1.34 | -2.26 | -0.41 | **<0.05** | 92 |
|  | PVB | 1 | N | N | N | N | N | N |
|  | PECS-2 block | 1 | N | N | N | N | N | N |
| LA infusion VS | No block | 7 | 608 | -0.75 | -1.56 | 0.06 | 0.07 | 95 |
| PECS-2 block VS | LA infusion | 3 | 175 | 0.13 | -0.79 | 1.06 | 0.78 | 87.3 |
|  | No block | 15 | 879 | -1.99 | -2.77 | -1.21 | **<0.05** | 95.7 |
|  | PVB | 7 | 324 | 0.25 | -0.36 | 0.85 | 0.42 | 86.2 |
|  | ESPB | 1 | N | N | N | N | N | N |
|  | SPB | 2 | 140 | 0.22 | -0.11 | 0.54 | 0.2 | 0 |
|  | RIB | N | N | N | N | N | N | N |
| PVB VS | IPB | 1 | N | N | N | N | N | N |
|  | LA infusion | 3 | 132 | -0.99 | -2.32 | 0.35 | 0.15 | 34.2 |
|  | No block | 16 | 1077 | -0.42 | -0.64 | -0.21 | **<0.05** | 67.5 |
|  | SPB | 3 | 18 | -0.07 | -0.58 | 0.43 | 0.78 | 56.7 |
| SPB VS | No block | 10 | 693 | -0.78 | -1.43 | -0.13 | **<0.02** | 93.4 |
| IPB VS | No block | 1 | N | N | N | N | N |  |
| SPB+PECS-1 block VS | No block | 1 | N | N | N | N | N | N |
|  | SPB | 1 | N | N | N | N | N | N |
| SPB+PECS-2 block VS | No block | 1 | N | N | N | N | N | N |

**Table 3. Postoperative 24-hour morphine consumption**

| **Interventions** |  | **No. of trials** | **No. of patients** | **MD** | **LL** | **UL** | ***P*** | ***I*^2^(%)** |
| --- | --- | --- | --- | --- | --- | --- | --- | --- |
| ESPB VS | No block | 6 | 400 | -1.84 | -2.56 | -1.13 | **<0.05** | 79.5 |
|  | PVB | 3 | 187 | -0.24 | -0.89 | 0.41 | 0.47 | 79.4 |
|  | PECS-2 block | 3 | 145 | 1.76 | 0.96 | 2.57 | <0.05 | 76.9 |
| S | SPB | 1 | N | N | N | N | N | N |
|  | RIB | N | N | N | N | N | N | N |
| IPB VS | No lock | 1 | N | N | N | N | N | N |
|  | PVB | 1 | N | N | N | N | N | N |
| PVB | LA infusion | 1 | N | N | N | N | N |  |
|  | No block | 14 | 577 | -1.73 | -2.4 | -1.06 | **<0.05** | 91.6 |
|  | SPB | 2 | 98 | -1.1 | -2.31 | 0.11 | 0.07 |  |
| PECS-2 block VS | LA infusion | 2 | 132 | 0.28 | -0.4 | 0.95 | 0.42 |  |
|  | No block | 16 | 1010 | -2.27 | -2.92 | -1.62 | **<0.05** | 94.3 |
|  | PVB | 9 | 503 | -0.48 | -1.2 | 0.24 | 0.19 | 91.6 |
|  | SPB | 1 | N | N | N | N | N | N |
|  | RIB | 1 | N | N | N | N | N | N |
| SPB VS | No block | 4 | 226 | -3.19 | -5.47 | -0.9 | **0.01** | 97 |
| RIB VS | No block | 1 | N | N | N | N | N | N |
| SPB+ PECS-1 block VS | No block | 1 | N | N | N |  | N | N |

**Table 4.** **Chronic pain incidence**

| **Interventions** | | **No. of trials** | **No. of patients** | **OR** | **LL** | **UL** | ***P*** | ***I^2^*(%)** |
| --- | --- | --- | --- | --- | --- | --- | --- | --- |
| LA VS | No block | 1 | N | N | N | N | N | N |
| PECS-2 block VS | No block | 3 | 160 | 0.38 | 0.11 | 1 | 0.051 | 49.2 |
|  | LA infusion | 1 | N | N | N | N | N | N |
|  | SPB | 1 | N | N | N | N | N | N |
| PVB VS | No block | 3 | 341 | 0.64 | 0.32 | 1.27 | 0.2 | 12.4 |

**Table 5. PONV (postoperative 24-hour)**

| **Interventions** |  | **No. of trials** | **No. of patients** | **OR** | **LL** | **UL** | **P** | ***I^2^*(%)** |
| --- | --- | --- | --- | --- | --- | --- | --- | --- |
| ESPB VS | No block | 3 | 190 | 0.38 | 0.14 | 0.91 | **0.031** | **41.6** |
|  | PVB | 1 | N | N | N | N | N | N |
|  | RIB | 1 | N | N | N | N | N | N |
|  | SPB | 1 | N | N | N | N | N | N |
| LA infusion VS | No block | 3 | 183 | 0.69 | 0.31 | 1.52 |  | 0 |
| IPB VS | No block | 1 | N | N | N | N | N | N |
|  | PVB | 2 | 100 | 0.46 | 0.11 | 1.98 | 0.3 | N |
| PECS-1 block VS | No block | 1 | N | N | N | N | N | N |
| PECS-2 block VS | No block | 6 | 314 | 0.31 | 0.17 | 0.56 | **<0.05** | 0 |
|  | PVB | 3 | 140 | 0.68 | 0.19 | 2.4 | 0.548 | 0 |
|  | RIB | 1 | N | N | N | N | N | N |
| PVB VS | No block | 10 | 512 | 0.32 | 0.2 | 0.52 | **<0.05** | **0** |
|  | LA infusion | 1 | N | N | N | N | N | N |
| SPB VS | No block | 1 | N | N | N | N | N | N |
| RIB VS | No block | 1 | N | N | N | N | N | N |

## Appendix 5

## Treatment’s efficacy (league) tables

Note: PECS-2 block: Pectoral nerve 2 block, PECS-1 block: Pectoral nerve 1 block, PVB: Paravertebral nerve block, ESPB: Erector spinae plane block, SPB: Serratus anterior plane block, RIB: Rhomboid intercostal block, IPB: Interpleural block, LA infusion: Local anesthetic infusion.

**Table1. Mean difference (MD) with 95%** **credible interval (CrI) of network meta-analysis for PACU pain scores**

| **PVB** |  |  |  |  |  |  |  |  |  |
| --- | --- | --- | --- | --- | --- | --- | --- | --- | --- |
| **0.71 (0.1, 1.32)** | **PECS-2 block** |  |  |  |  |  |  |  |  |
| 0.81 (-0.26, 1.85) | 0.11 (-0.95, 1.13) | **ESPB** |  |  |  |  |  |  |  |
| -0.06 (-0.96, 0.84) | -0.78 (-1.62, 0.09) | -0.88 (-2.11, 0.38) | **SPB** |  |  |  |  |  |  |
| -1.06 (-3.97, 1.91) | -1.78 (-4.67, 1.19) | -1.86 (-4.93, 1.24) | -0.99 (-3.75, 1.77) | **SPB+PECS-1 block** |  |  |  |  |  |
| 0.28 (-0.66, 1.22) | -0.43 (-1.32, 0.45) | -0.53 (-1.8, 0.74) | 0.35 (-0.79, 1.45) | 1.34 (-1.73, 4.35) | **LA infusion** |  |  |  |  |
| 0.98 (-0.61, 2.58) | 0.27 (-1.31, 1.83) | 0.17 (-1.39, 1.72) | 1.04 (-0.71, 2.76) | 2.03 (-1.27, 5.29) | 0.7 (-1.05, 2.44) | **RIB** |  |  |  |
| -1 (-3.28, 1.31) | -1.71 (-3.98, 0.59) | -1.82 (-4.26, 0.66) | -0.94 (-3.27, 1.41) | 0.07 (-3.61, 3.67) | -1.29 (-3.65, 1.12) | -1.99 (-4.68, 0.69) | **SPB+PECS-2 block** |  |  |
| -1.49 (-3.87, 0.83) | -2.19 (-4.58, 0.14) | -2.28 (-4.8, 0.16) | -1.42 (-3.87, 0.97) | -0.44 (-4.17, 3.26) | -1.76 (-4.23, 0.66) | -2.46 (-5.22, 0.28) | -0.48 (-3.61, 2.7) | **PECS-1 block** |  |
| **-1.49 (-2.05, -0.94)** | **-2.2 (-2.74, -1.67)** | **-2.3 (-3.27, -1.3)** | **-1.43 (-2.21, -0.64)** | -0.43 (-3.36, 2.45) | **-1.77 (-2.61, -0.93)** | **-2.47 (-4, -0.91)** | -0.48 (-2.71, 1.72) | -0.01 (-2.25, 2.33) | **No block** |

**Table 2.** **MD with 95% CrI of network meta-analysis for postoperative 24-hour pain scores**

| **PVB** |  |  |  |  |  |  |  |  |
| --- | --- | --- | --- | --- | --- | --- | --- | --- |
| 0.12 (-0.3, 0.54) | **PECS-2 block** |  |  |  |  |  |  |  |
| 0.17 (-0.51, 0.84) | 0.05 (-0.61, 0.71) | **ESPB** |  |  |  |  |  |  |
| 0.14 (-0.38, 0.64) | 0.02 (-0.49, 0.53) | -0.03 (-0.73, 0.67) | **SPB** |  |  |  |  |  |
| -0.22 (-1.56, 1.11) | -0.34 (-1.68, 1) | -0.39 (-1.82, 1.04) | -0.36 (-1.67, 0.93) | **SPB+PECS-1 block** |  |  |  |  |
| 0.19 (-0.37, 0.75) | 0.07 (-0.5, 0.63) | 0.02 (-0.74, 0.78) | 0.05 (-0.59, 0.68) | 0.41 (-0.97, 1.8) | **LA infusion** |  |  |  |
| 0.01 (-1.08, 1.11) | -0.11 (-1.18, 0.96) | -0.17 (-1.29, 0.96) | -0.13 (-1.23, 0.96) | 0.23 (-1.42, 1.9) | -0.19 (-1.33, 0.98) | **RIB** |  |  |
| 0 (-1.74, 1.76) | -0.12 (-1.89, 1.71) | -0.17 (-2.03, 1.71) | -0.14 (-1.93, 1.68) | 0.22 (-1.96, 2.42) | -0.19 (-2.01, 1.66) | -0.01 (-2.04, 2.05) | **IPB** |  |
| **-0.63 (-0.98, -0.28)** | **-0.75 (-1.11, -0.39)** | **-0.8 (-1.39, -0.2)** | **-0.77 (-1.19, -0.34)** | -0.42 (-1.7, 0.9) | **-0.82 (-1.31, -0.33)** | -0.64 (-1.71, 0.41) | -0.63 (-2.43, 1.13) | **No block** |

**Table 3.** **MD with 95%CrI of network meta-analysis for postoperative 24-hour morphine consumption**

| **PVB** |  |  |  |  |  |  |  |  |
| --- | --- | --- | --- | --- | --- | --- | --- | --- |
| 1.68 (-1.32, 4.65) | **PECS-2 block** |  |  |  |  |  |  |  |
| 0.79 (-3, 4.6) | -0.9 (-4.69, 2.84) | **ESPB** |  |  |  |  |  |  |
| 1.37 (-2.91, 5.62) | -0.31 (-4.56, 3.89) | 0.59 (-4.04, 5.27) | **SPB** |  |  |  |  |  |
| 1.79 (-3.93, 7.44) | 0.13 (-5.43, 5.67) | 1.03 (-5.14, 7.19) | 0.42 (-6.01, 6.94) | **LA infusion** |  |  |  |  |
| 3.3 (-2.88, 9.66) | 1.65 (-4.32, 7.62) | 2.5 (-3.96, 9.01) | 1.93 (-4.69, 8.58) | 1.53 (-6.36, 9.29) | **RIB** |  |  |  |
| -0.39 (-12.67, 12.04) | -2.09 (-14.66, 10.77) | -1.22 (-14.03, 11.81) | -1.74 (-14.84, 11.34) | -2.23 (-15.79, 11.33) | -3.74 (-17.5, 10.2) | **IPB** |  |  |
| -7.07 (-19.82, 5.74) | -8.72 (-21.43, 3.98) | -7.84 (-20.75, 5.07) | -8.45 (-21.41, 4.52) | -8.83 (-22.39, 4.8) | -10.41 (-24.3, 3.34) | -6.65 (-24.3, 10.81) | **SPB+PECS-2 block** |  |
| **-7.14 (-9.78, -4.47)** | **-8.81 (-11.43, -6.15)** | **-7.93 (-11.29, -4.44)** | **-8.5 (-12.37, -4.61)** | **-8.93 (-14.29, -3.58)** | **-10.46 (-16.43, -4.58)** | -6.72 (-19.48, 5.81) | -0.07 (-12.56, 12.34) | No block |

**Table 4.** **Odds ratio (OR) with 95%CrI of network meta-analysis for incidence of chronic pain**

| **PVB** |  |  |  |  |
| --- | --- | --- | --- | --- |
| 1.83 (0.33, 10.25) | **PECS-2 block** |  |  |  |
| 2.81 (0.28, 29.72) | 1.55 (0.22, 11.59) | **SPB** |  |  |
| 0.63 (0.09, 5.1) | 0.34 (0.08, 1.88) | 0.22 (0.02, 2.44) | **LA infusion** |  |
| 0.68 (0.21, 2.46) | 0.37 (0.11, 1.28) | 0.24 (0.03, 1.73) | 1.09 (0.22, 4.68) | **No block** |

**Table 5. OR with 95%CI of network meta-analysis for PONV (postoperative 24-hour)**

| **PVB** |  |  |  |  |  |  |  |  |
| --- | --- | --- | --- | --- | --- | --- | --- | --- |
| 0.93 (0.39, 2.31) | **PECS-2 block** |  |  |  |  |  |  |  |
| 0.92 (0.45, 1.99) | 0.99 (0.35, 2.81) | **PECS-1 block** |  |  |  |  |  |  |
| 1.08 (0.54, 2.11) | 1.15 (0.42, 2.99) | 1.17 (0.47, 2.69) | **ESPB** |  |  |  |  |  |
| 1.05 (0.41, 2.78) | 1.13 (0.35, 3.69) | 1.13 (0.39, 3.34) | 0.97 (0.37, 2.62) | **SPB** |  |  |  |  |
| 0.35 (0.06, 1.78) | 0.36 (0.06, 2.18) | 0.37 (0.06, 2.04) | 0.32 (0.06, 1.78) | 0.32 (0.05, 1.97) | **IPB** |  |  |  |
| 1.71 (0.59, 5.57) | 1.85 (0.59, 6.4) | 1.85 (0.56, 6.68) | 1.59 (0.55, 5.22) | 1.63 (0.49, 5.7) | 5.05 (0.77, 38.02) | **RIB** |  |  |
| 0.51 (0.21, 1.23) | 0.54 (0.18, 1.68) | 0.55 (0.2, 1.52) | 0.47 (0.18, 1.26) | 0.49 (0.15, 1.48) | 1.5 (0.25, 9.24) | 0.3 (0.08, 1.01) | **LA infusion** |  |
| **0.35 (0.22, 0.53)** | **0.37 (0.16, 0.81)** | 0.8 (0.3, 2.14) | **0.32 (0.18, 0.58)** | **0.33 (0.14, 0.76)** | 1 (0.21, 5.33) | **0.2 (0.07, 0.54)** | 0.68 (0.31, 1.48) | No block |

## Appendix 6

## Cumulative ranking probability (SCURA)

**Table 1. cumulative ranking probability**

| **PACU pain scores** | | **Postoperative 24-hour pain scores** | | **Postoperative 24-hour morphine consumption** | | **PONV** | |
| --- | --- | --- | --- | --- | --- | --- | --- |
| **Treatment** | **SUCRA (%)** | **Treatment** | **SUCRA (%)** | **Treatment** | **SUCRA (%)** | **Treatment** | **SUCRA (%)** |
| RIB | 84.8 | SPB | 66.3 | RIB | 79.5 | RIB | 88.8 |
| ESPB | 83.3 | LA infusion | 65.9 | PECS-2 block | 67.7 | ESPB | 66.4 |
| PECS-2 block | 81.8 | ESPB | 64.4 | LA infusion | 65.9 | SPB | 64.7 |
| LA infusion | 62.8 | PECS-2 block | 58 | SPB | 63.3 | PVB | 64.4 |
| PVB | 49 | RIB | 51.3 | ESPB | 55.2 | PECS-2 block | 59 |
| SPB | 48.9 | IPB | 50.3 | IPB | 50.1 | PECS-1 block | 54 |
| SPB+PECS-1 block | 29.2 | PVB | 46.6 | PVB | 43.9 | LA infusion | 26.1 |
| SPB+PECS-2 block | 28.4 | SPB+PECS-1 block | 39.5 | SPB+PECS-2 block | 16.4 | IPB | 18.5 |
| PECS-1 block | 18.4 | No block | 7.6 | No block | 8 | No block | 8.1 |
| No block | 13.4 |  |  |  |  |  |  |

Note: PECS-2 block: Pectoral nerve 2 block, PECS-1 block: Pectoral nerve 1 block, PVB: Paravertebral nerve block, ESPB: Erector spinae plane block, SPB: Serratus anterior plane block, RIB: Rhomboid intercostal block, IPB: Interpleural block, LA infusion: Local anesthetic infusion.

## Appendix 7

## Assessment of global inconsistency results


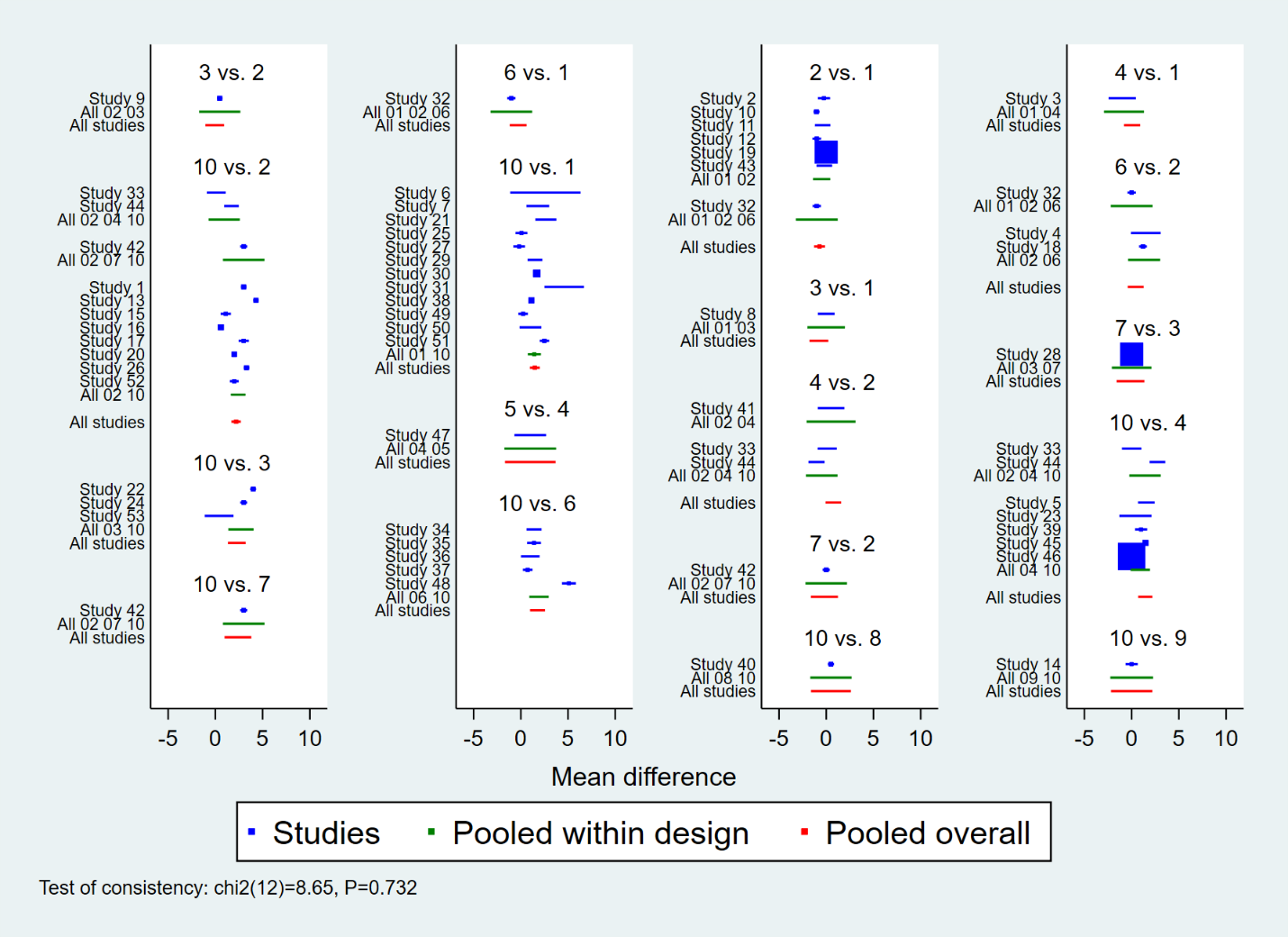


**Figure 1. Assessment of inconsistency results for PACU pain scores(*P*=0.732)**

Notes: 1: PVB, 2: PECS-2 block, 3: ESPB, 4: SPB, 5: SPB+PECS-1 block 6: LA infusion, 7: RIB, 8: SPB+PECS-2 block, 9: PECS-1 block, 10: No block


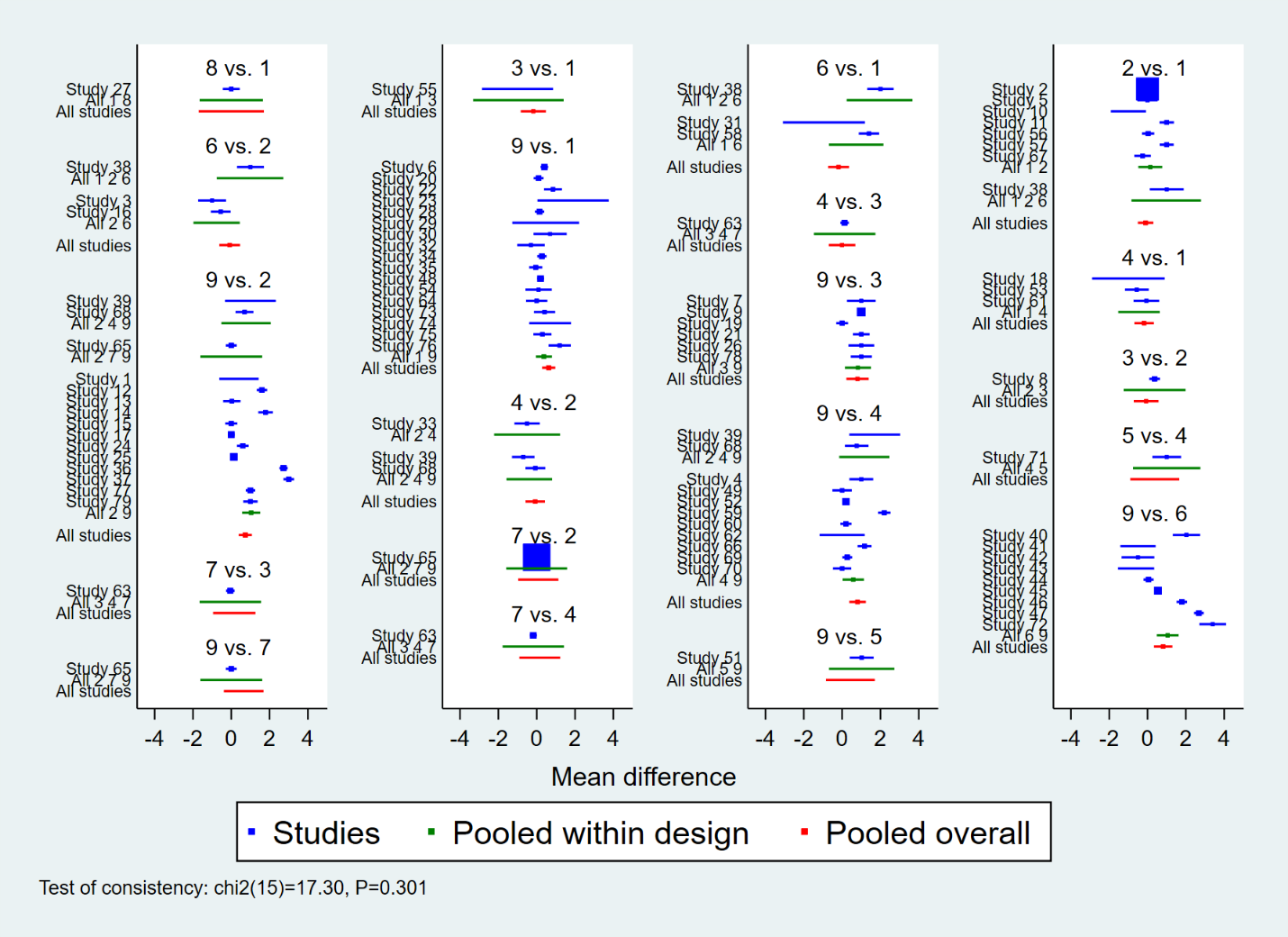


**Figure 2. Assessment of inconsistency results for 24-hour pain scores(*P*=0.301)**

Notes: 1: PVB, 2: PECS-2 block, 3: ESPB, 4: SPB, 5: SPB+PECS-1 block, 6: LA infusion, 7: RIB, 8: IPB, 9: No block.


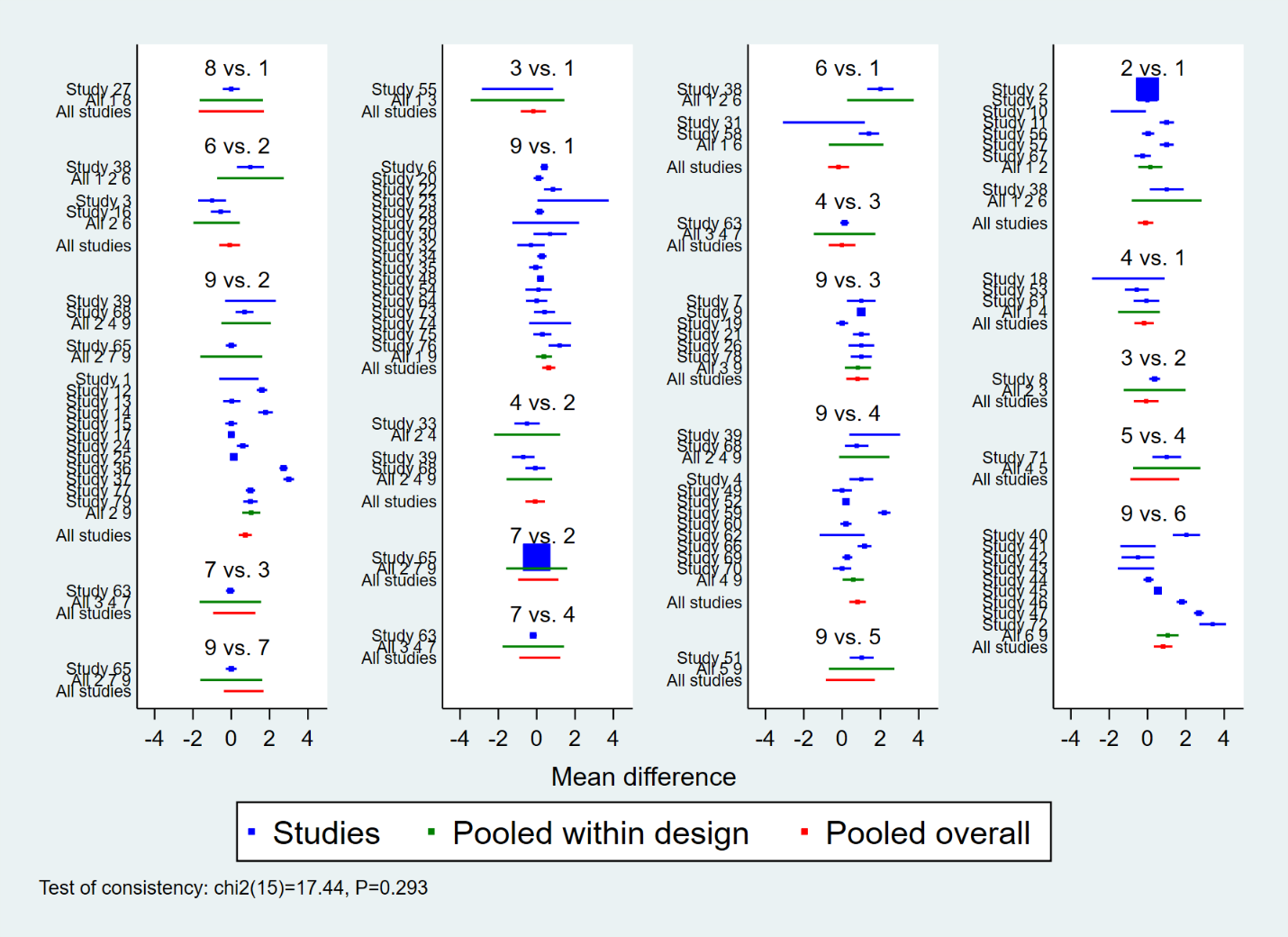


**Figure 3. Assessment of inconsistency results for postoperative 24-hour morphine consumption(*P*=0.293)**

Notes: 1: PVB, 2: PECS-2 block, 3: ESPB, 4: SPB, 5: LA infusion, 6: RIB, 7: IPB, 8: SPB+PECS-2 block, 9: No block.


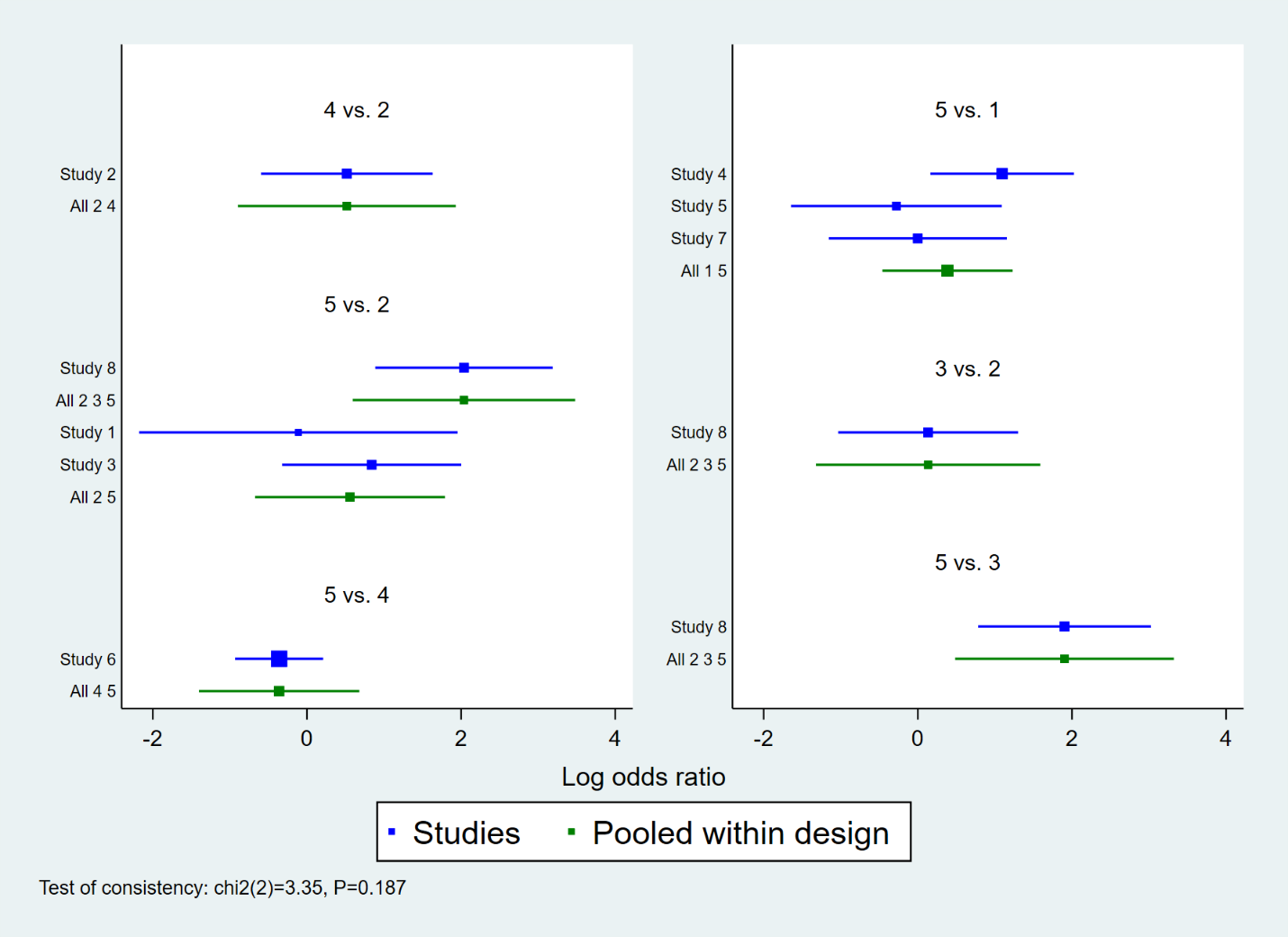


**Figure 4. Assessment of inconsistency results for incidence of chronic pain (*P*=0.187)**

Notes: 1: PVB, 2: PECS-2 block, 3: SPB, 4 LA infusion, 5: No block.

## Appendix 8

## Assessment of local inconsistency results by node-splitting method


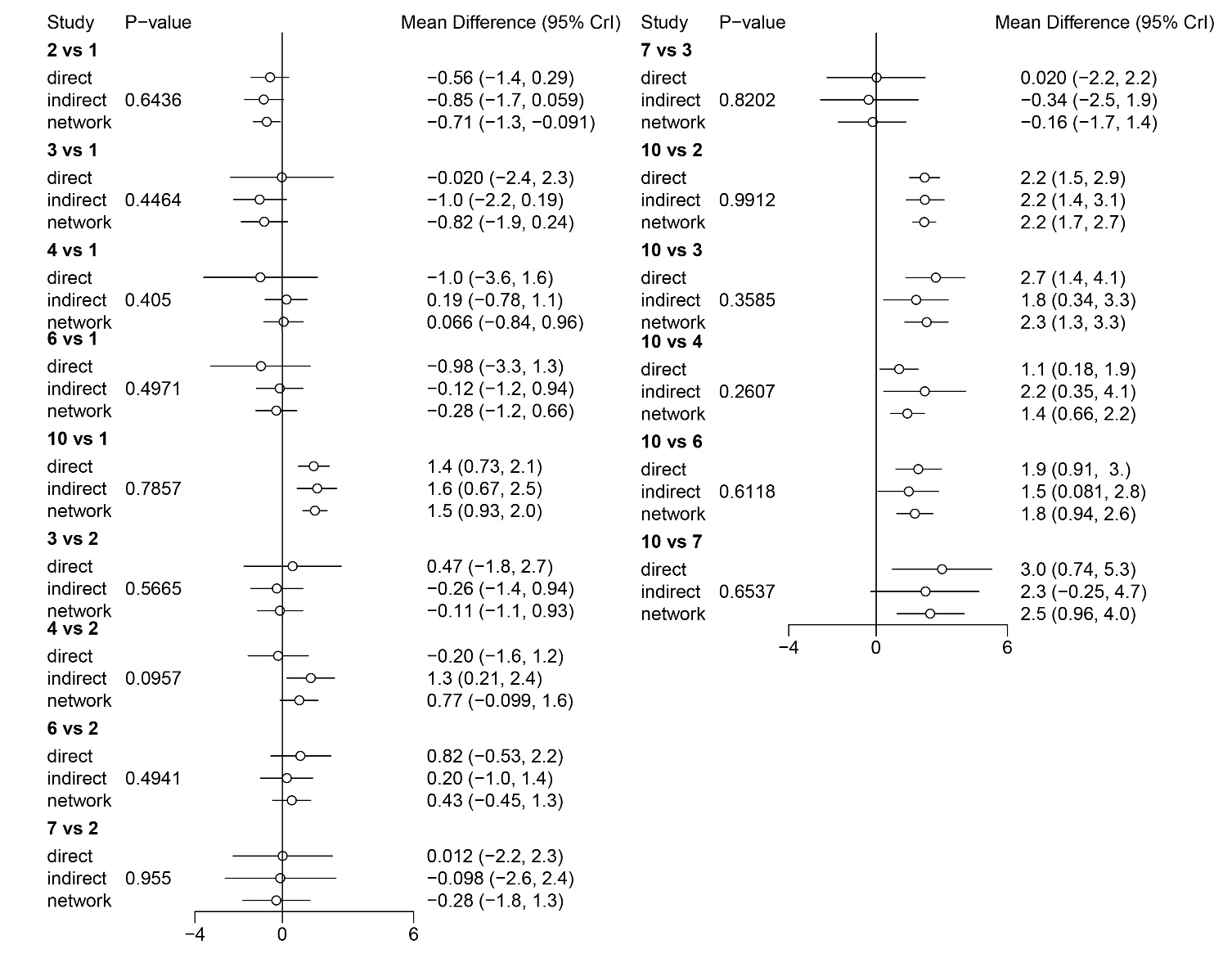


**Figure 1. PACU pain scores**

Notes: 1: PVB, 2: PECS-2 block, 3: ESPB, 4: SPB, 5: SPB+PECS-1 block 6: LA infusion, 7: RIB, 8: SPB+PECS-2 block, 9: PECS-1 block, 10: No block


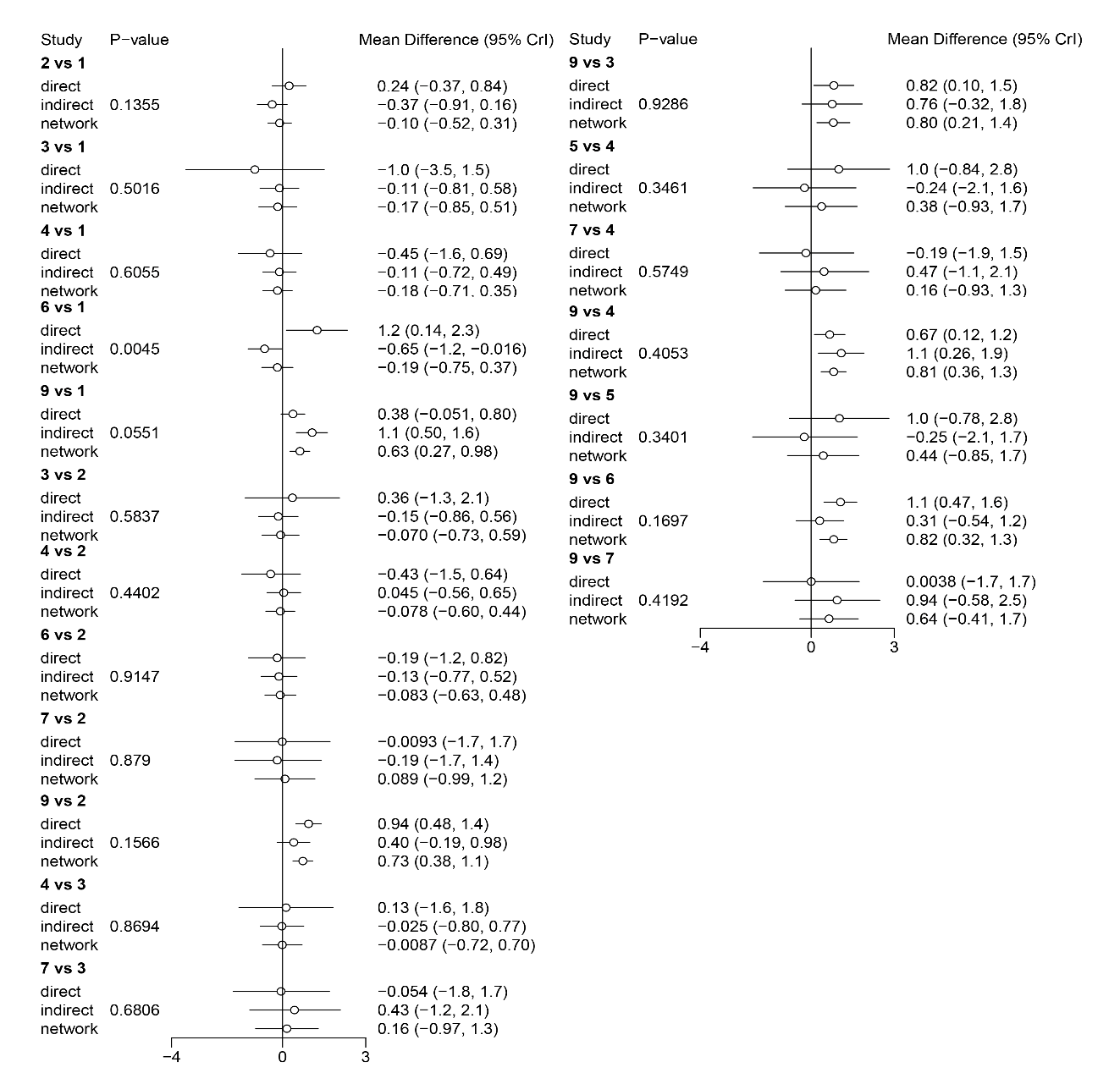


**Figure 2. Postoperative 24-hour pain scores**

Notes: 1: PVB, 2: PECS-2 block, 3: ESPB, 4: SPB, 5: SPB+PECS-1 block, 6: LA infusion, 7: RIB, 8: IPB, 9: SPB+PECS-2 block, 10: No block.

**
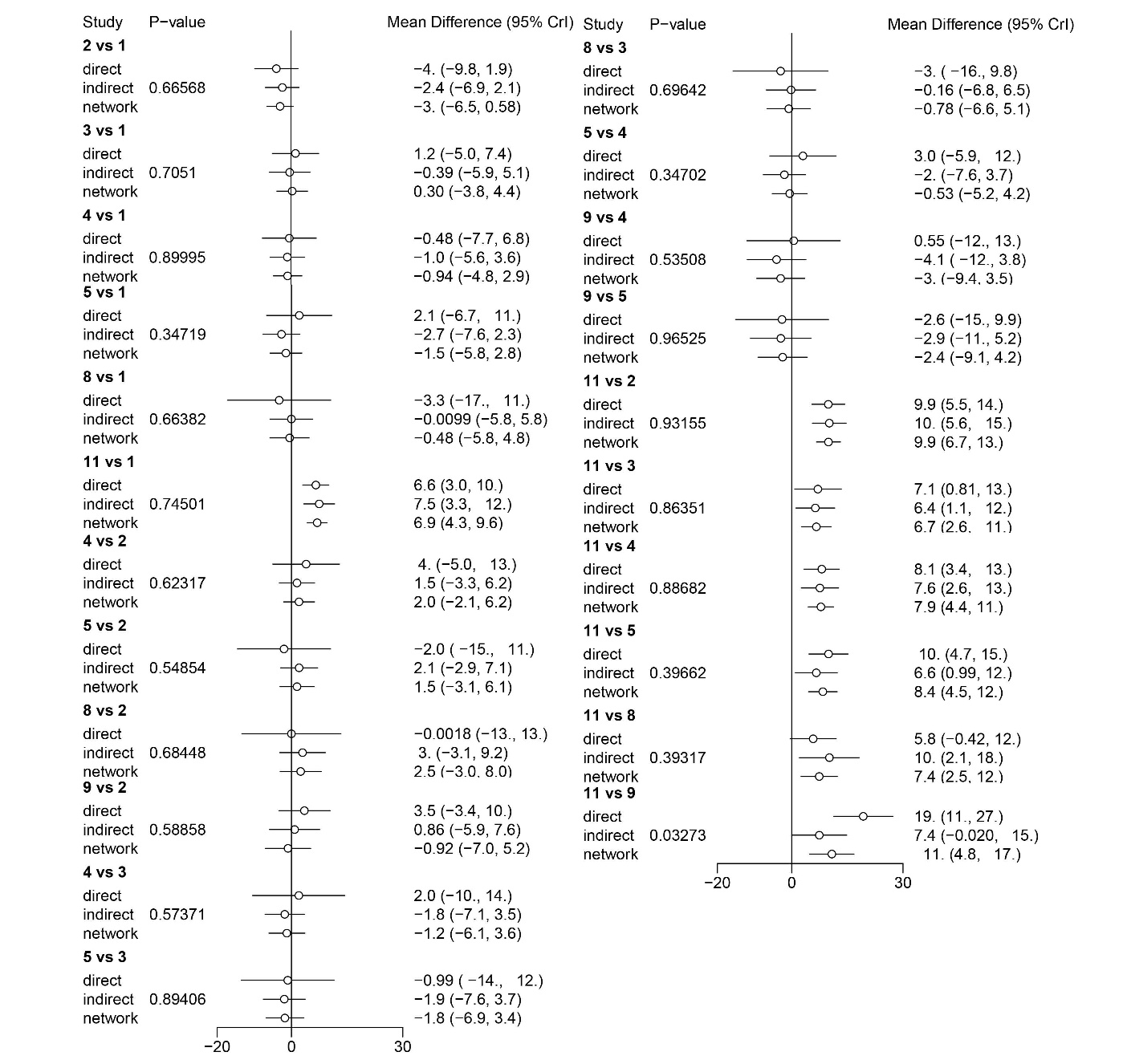
**

**Figure 3. Postoperative 24-hour morphine consumption**

Notes: 1: PVB, 2: PECS-2 block, 3: ESPB, 4: SPB, 5: SPB+PECS-1 block, 6: SPB+PECS-2 block, 7: LA infusion, 8: RIB, 9: IPB; 10: No block.


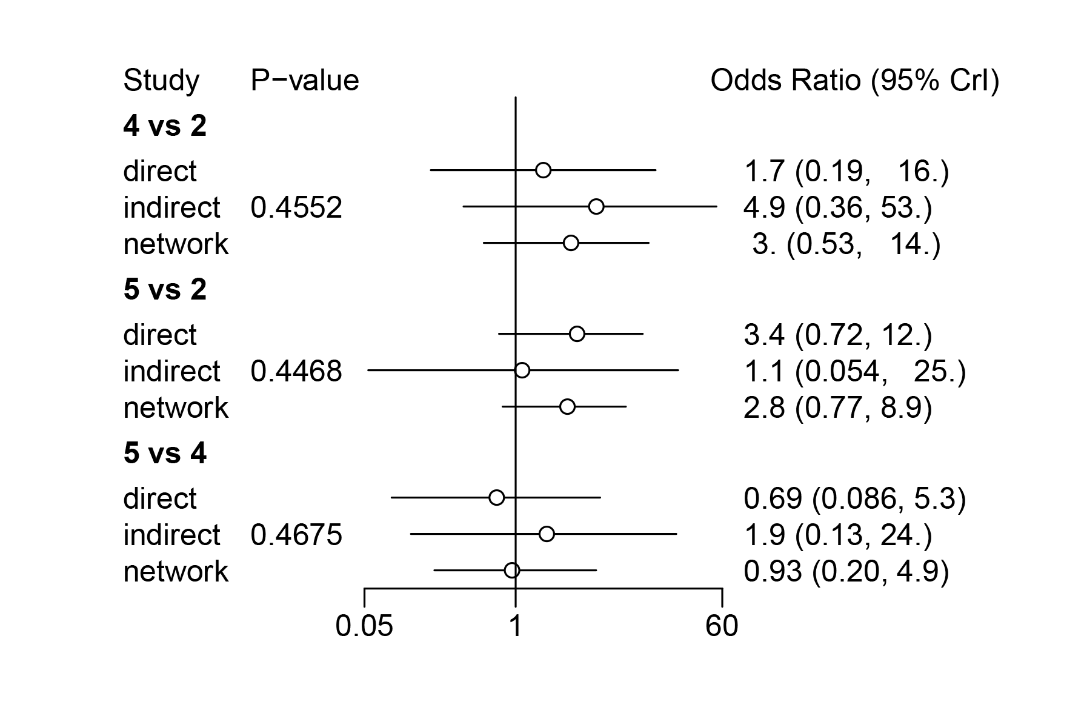


**Figure 4. Incidence of chronic pian**

Notes: 1: PVB, 2: PECS-2 block, 3: SPB, 4: IPB, 5: LA infusion, 6: No block.


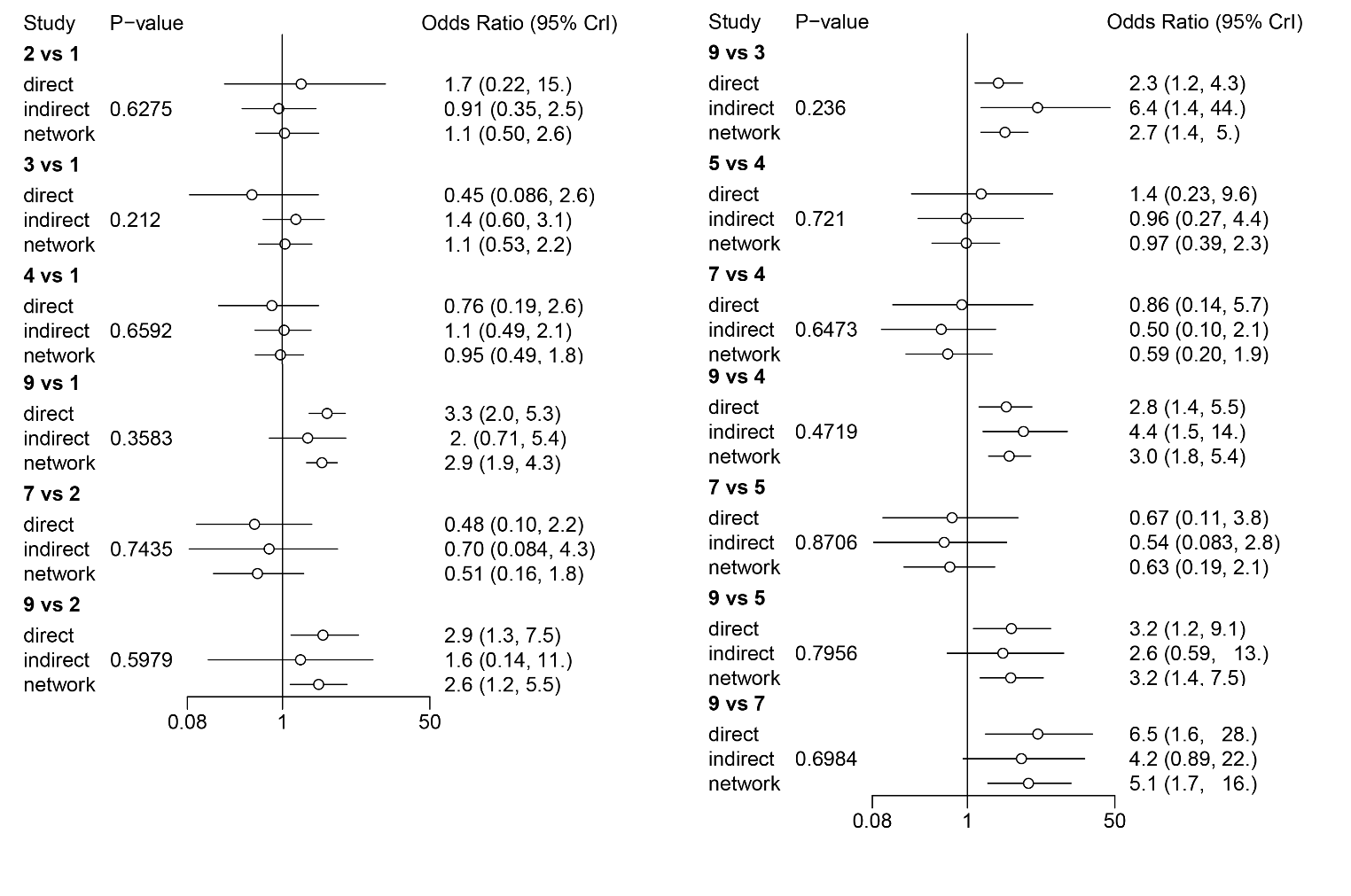


**Figure 5. Incidence of PONV**

Notes: 1: PVB, 2: PECS-2 block, 3: PECS-1 block, 4: ESPB, 5: SPB, 6: IPB, 7: RIB, 8: LA infusion, 9: No block.

## Appendix 9

## Evaluation of inconsistency using loop-specific heterogeneity estimates

**Table 1. PACU pain scores**

| **Loop** | **IF** | **seIF** | **z_value** | **p_value** | **CI_95** | **Loop_Heterog_tau2** |
| --- | --- | --- | --- | --- | --- | --- |
| ESPB-No block-PVB | 1.692 | 1.389 | 1.218 | 0.223 | (0.00,4.41) | 0.361 |
| No block-PECS-2 block-SPB | 1.617 | 1.019 | 1.586 | 0.113 | (0.00,3.62) | 1.537 |
| No block-PVB-SPB | 1.292 | 1.38 | 0.937 | 0.349 | (0.00,4.00) | 0.622 |
| LA infusion-PECS-2 block-PVB | 1.272 | 0.839 | 1.517 | 0.129 | (0.00,2.92) | 0.393 |
| ESPB-No block-PECS-2 block | 0.893 | 1.624 | 0.55 | 0.582 | (0.00,4.08) | 1.789 |
| No block-PECS-2 block-RIB | 0.868 | 2.069 | 0.42 | 0.675 | (0.00,4.92) | 2 |
| LA infusion-No block-PECS-2 block | 0.565 | 1.139 | 0.496 | 0.62 | (0.00,2.80) | 1.908 |
| ESPB-PECS-2 block-RIB | 0.46 | 0.238 | 1.936 | 0.053 | (0.00,0.93) | 0 |
| LA infusion-No block-PVB | 0.396 | 1.373 | 0.288 | 0.773 | (0.00,3.09) | 0.511 |
| No block-PECS-2 block-PVB | 0.299 | 0.597 | 0.501 | 0.617 | (0.00,1.47) | 0.911 |
| ESPB-No block-RIB | 0.22 | 2.424 | 0.091 | 0.928 | (0.00,4.97) | 0.86 |
| PECS-2 block-PVB-SPB | 0.189 | 1.106 | 0.171 | 0.864 | (0.00,2.36) | 0.382 |
| ESPB-PECS-2 block-PVB | 0.106 | 1.028 | 0.103 | 0.918 | (0.00,2.12) | 0.382 |

**Table 2. Postoperative 24-hour pain scores**

| **Loop** | **IF** | **seIF** | **z_value** | **p_value** | **CI_95** | **Loop_Heterog_tau2** |
| --- | --- | --- | --- | --- | --- | --- |
| LA infusion-No block-PVB | 1.979 | 0.727 | 2.721 | **0.007** | (0.55,3.40) | 0.526 |
| ESPB-PECS-2 block-PVB | 1.621 | 1.475 | 1.099 | 0.272 | (0.00,4.51) | 0.221 |
| LA infusion-PECS-2 block-PVB | 1.412 | 0.785 | 1.799 | 0.072 | (0.00,2.95) | 0.264 |
| No block-SPB-SPB+PECS-1 block | 1.358 | 1.129 | 1.203 | 0.229 | (0.00,3.57) | 0.487 |
| No block-PECS-2 block-RIB | 1.007 | 1.612 | 0.624 | 0.532 | (0.00,4.17) | 1.242 |
| ESPB-PECS-2 block-SPB | 0.895 | 0.36 | 2.49 | **0.013** | (0.19,1.60) | 0.028 |
| ESPB-No block-RIB | 0.857 | 0.699 | 1.227 | 0.22 | (0.00,2.23) | 0.205 |
| No block-RIB-SPB | 0.842 | 1.028 | 0.819 | 0.413 | (0.00,2.86) | 0.487 |
| No block-PECS-2 block-PVB | 0.803 | 0.424 | 1.894 | 0.058 | (0.00,1.63) | 0.638 |
| No block-PECS-2 block-SPB | 0.706 | 0.733 | 0.963 | 0.335 | (0.00,2.14) | 0.988 |
| PECS-2 block-RIB-SPB | 0.575 | 0.324 | 1.775 | 0.076 | (0.00,1.21) | 0.028 |
| ESPB-No block-PVB | 0.525 | 1.052 | 0.499 | 0.618 | (0.00,2.59) | 0.077 |
| ESPB-PVB-SPB | 0.505 | 0.981 | 0.514 | 0.607 | (0.00,2.43) | 0 |
| ESPB-PECS-2 block-RIB | 0.32 | 0.184 | 1.744 | 0.081 | (0.00,0.68) | 0 |
| ESPB-No block-PECS-2 block | 0.258 | 1.139 | 0.227 | 0.821 | (0.00,2.49) | 1.023 |
| PECS-2 block-PVB-SPB | 0.238 | 0.565 | 0.421 | 0.674 | (0.00,1.34) | 0.198 |
| LA infusion-No block-PECS-2 block | 0.097 | 0.854 | 0.114 | 0.909 | (0.00,1.77) | 1.235 |
| No block-PVB-SPB | 0.087 | 0.44 | 0.198 | 0.843 | (0.00,0.95) | 0.209 |
| ESPB-No block-SPB | 0.028 | 0.724 | 0.038 | 0.969 | (0.00,1.45) | 0.392 |

**Table 3. Postoperative 24-hour morphine consumption**

| **Loop** | **IF** | **seIF** | **z_value** | **p_value** | **CI_95** | **Loop_Heterog_tau2** |
| --- | --- | --- | --- | --- | --- | --- |
| No block-PECS-2 block-RIB | 15.883 | 7.372 | 2.155 | **0.031** | (1.43,30.33) | 14.94 |
| ESPB-No block-RIB | 11.97 | 12.283 | 0.975 | 0.33 | (0.00,36.04) | 48.115 |
| No block-RIB-SPB | 7.369 | 14.985 | 0.492 | 0.623 | (0.00,36.74) | 82.925 |
| ESPB-PECS-2 block-SPB | 6.548 | 0.921 | 7.108 | **0** | (4.74,8.35) | 0.147 |
| PECS-2 block-RIB-SPB | 6.406 | 1.264 | 5.07 | **0** | (3.93,8.88) | 0.437 |
| No block-PVB-SPB | 5.592 | 5.134 | 1.089 | 0.276 | (0.00,15.65) | 21.756 |
| ESPB-No block-SPB | 4.918 | 6.131 | 0.802 | 0.422 | (0.00,16.93) | 34.586 |
| PECS-2 block-PVB-SPB | 4.849 | 3.674 | 1.32 | 0.187 | (0.00,12.05) | 4.695 |
| LA infusion-No block-PECS-2 block | 2.796 | 8.748 | 0.32 | 0.749 | (0.00,19.94) | 17.201 |
| ESPB-No block-PECS-2 block | 2.495 | 6.151 | 0.406 | 0.685 | (0.00,14.55) | 17.484 |
| ESPB-PECS-2 block-PVB | 2.223 | 2.862 | 0.777 | 0.437 | (0.00,7.83) | 3.117 |
| LA infusion-No block-PVB | 2.195 | 6.677 | 0.329 | 0.742 | (0.00,15.28) | 11.203 |
| ESPB-PVB-SPB | 1.13 | 1.127 | 1.003 | 0.316 | (0.00,3.34) | 0.528 |
| ESPB-No block-PVB | 0.993 | 3.714 | 0.267 | 0.789 | (0.00,8.27) | 17.125 |
| No block-PECS-2 block-PVB | 0.73 | 3.661 | 0.199 | 0.842 | (0.00,7.91) | 13.436 |
| ESPB-PECS-2 block-RIB | 0.399 | 1.569 | 0.254 | 0.799 | (0.00,3.48) | 0.356 |
| No block-PECS-2 block-SPB | 0.324 | 8.023 | 0.04 | 0.968 | (0.00,16.05) | 24.739 |
| LA infusion-PECS-2 block-PVB | 0.214 | 6.939 | 0.031 | 0.975 | (0.00,13.81) | 5.463 |
| ESPB-RIB-SPB | 0.1 | 0.828 | 0.121 | 0.904 | (0.00,1.72) | 0 |

**Table 4. Incidence of chronic pain**

| **Loop** | **IF** | **seIF** | **z_value** | **p_value** | **CI_95** | **Loop_Heterog_tau2** |
| --- | --- | --- | --- | --- | --- | --- |
| No block-PECS-2 block-SPB | 1.426 | 0.974 | 1.463 | 0.143 | (0.00,3.34) | 0 |
| LA infusion-No block-PECS-2 block | 0.964 | 1.324 | 0.728 | 0.467 | (0.00,3.56) | 0.48 |

**Figure 5. Incidence of PONV (postoperative 24-hour)**

| **Loop** | **IF** | **seIF** | **z_value** | **p_value** | **CI_95** | **Loop_Heterog_tau2** |
| --- | --- | --- | --- | --- | --- | --- |
| ESPB-No block-RIB | 0.807 | 1.343 | 0.601 | 0.548 | (0.00,3.44) | 0.223 |
| PECS-1 block-ESPB-No block | 0.565 | 0.697 | 0.811 | 0.418 | (0.00,1.93) | 0 |
| ESPB-No block-SPB | 0.55 | 1.158 | 0.475 | 0.635 | (0.00,2.82) | 0.18 |
| No block-RIB-SPB | 0.301 | 1.148 | 0.262 | 0.793 | (0.00,2.55) | 0 |
| PECS-2 block-No block-RIB | 0.004 | 1.151 | 0.003 | 0.997 | (0.00,2.26) | 0 |

## Appendix 10

## Comparison-adjusted funnel plot


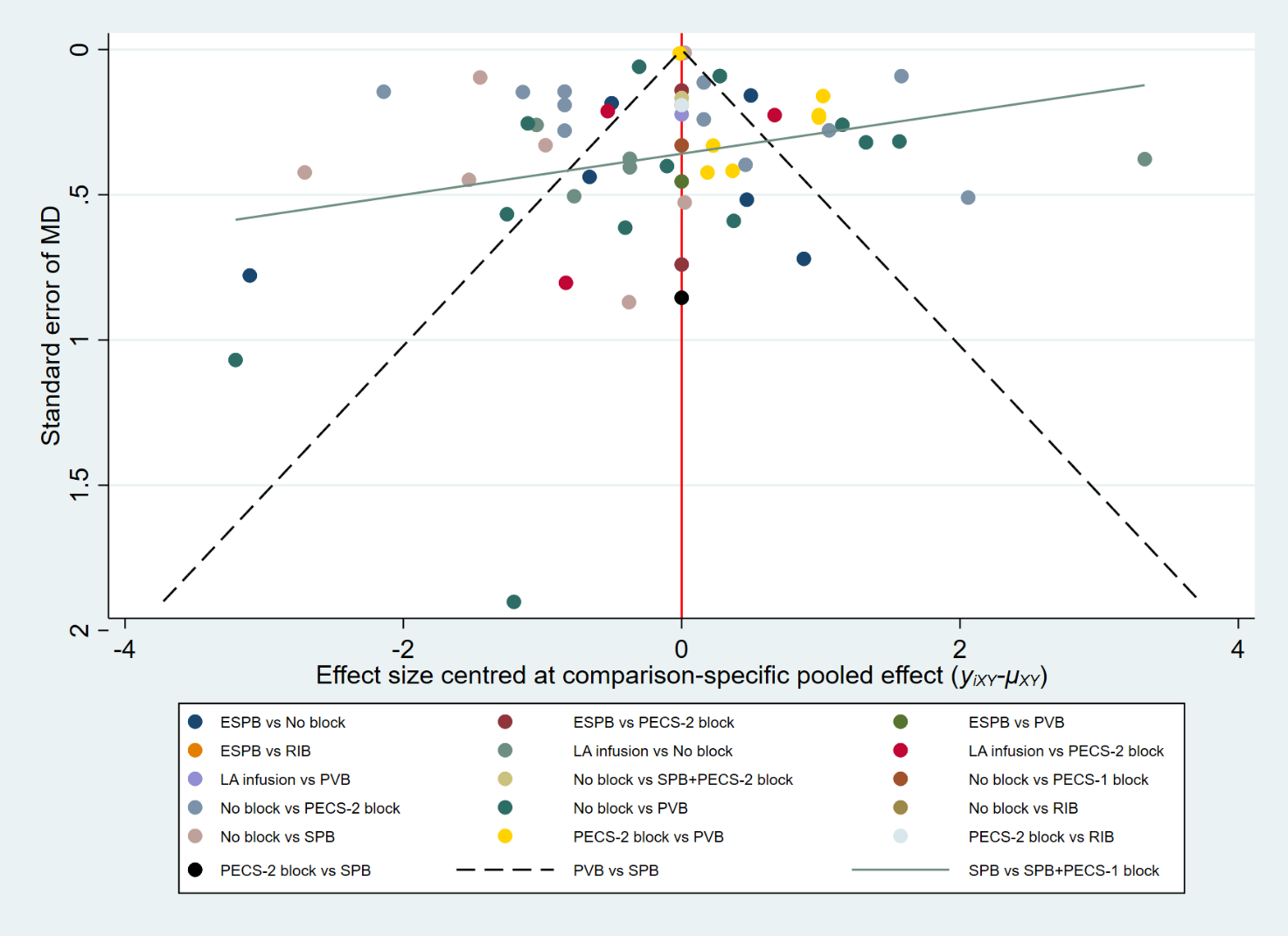


**Figure 1. PACU pain scores**

**
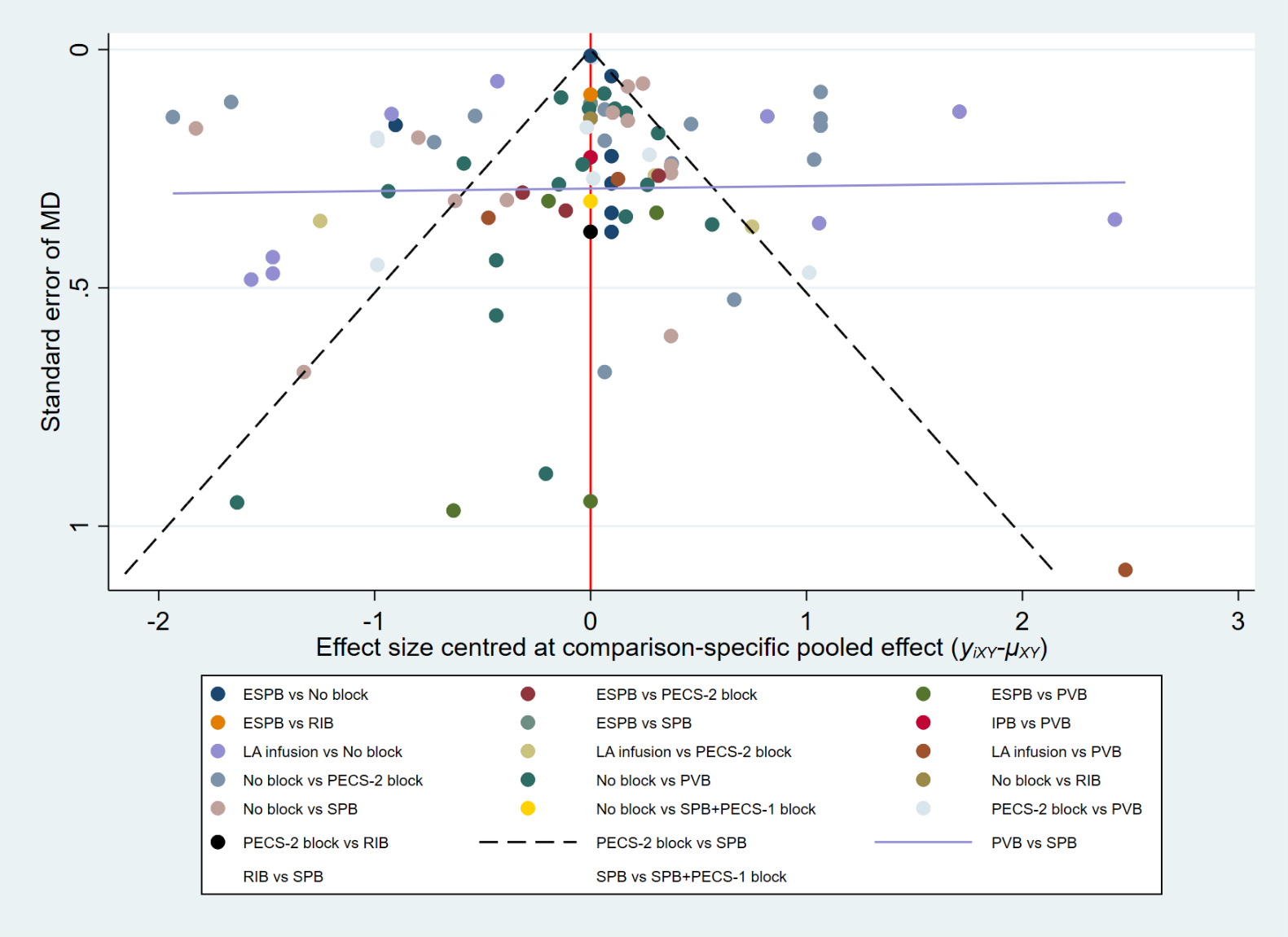
**

**Figure 2. Postoperative 24-hour pain scores**

**
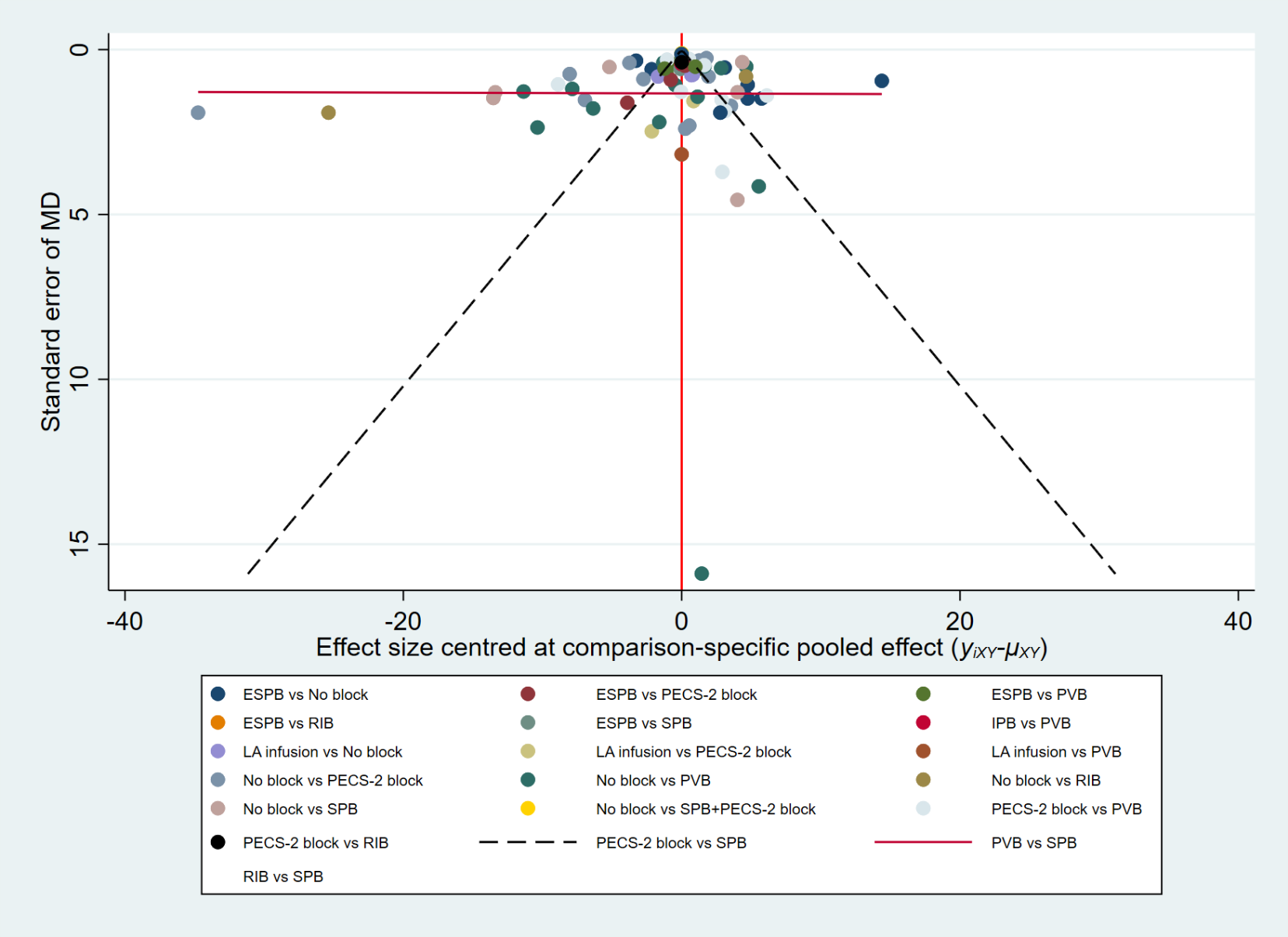
**

**Figure 3. Postoperative 24-hour morphine consumption**

**
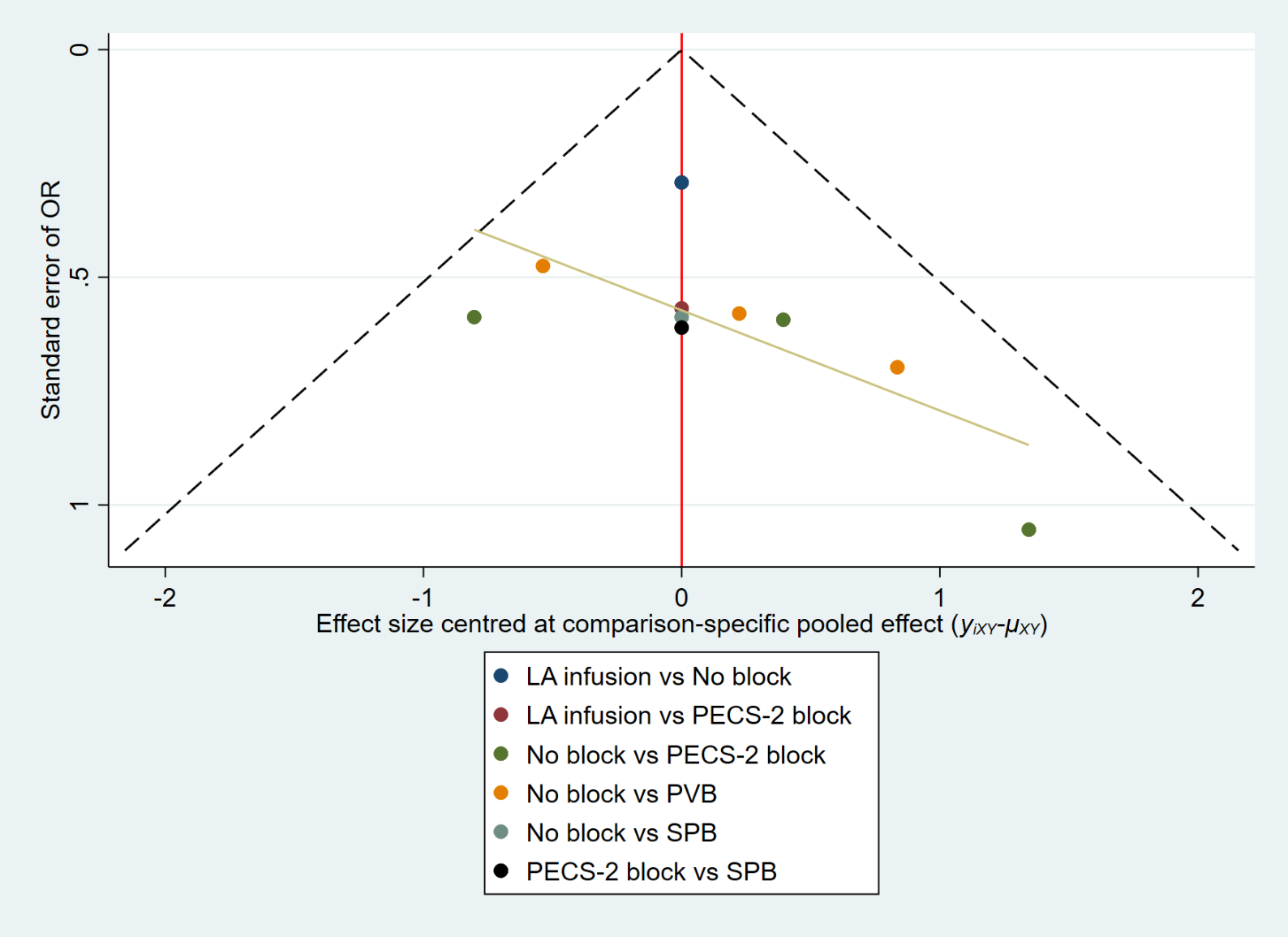
**

**Figure 4. Incidence of chronic pain**

**
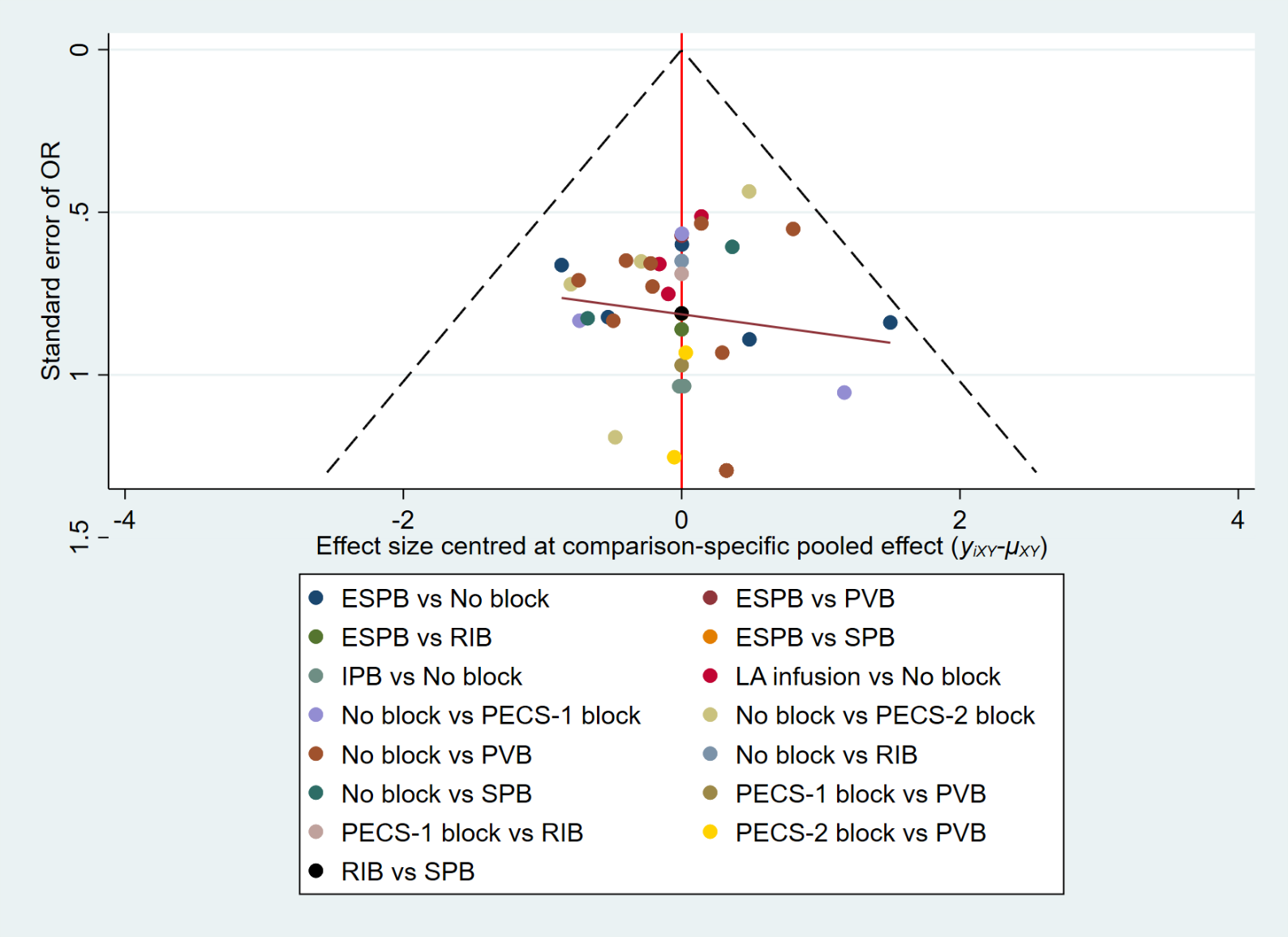
**

**Figure 5. Incidence of PONV (postoperative 24-hour)**

## Appendix 11

**Sensitivity analysis**

**
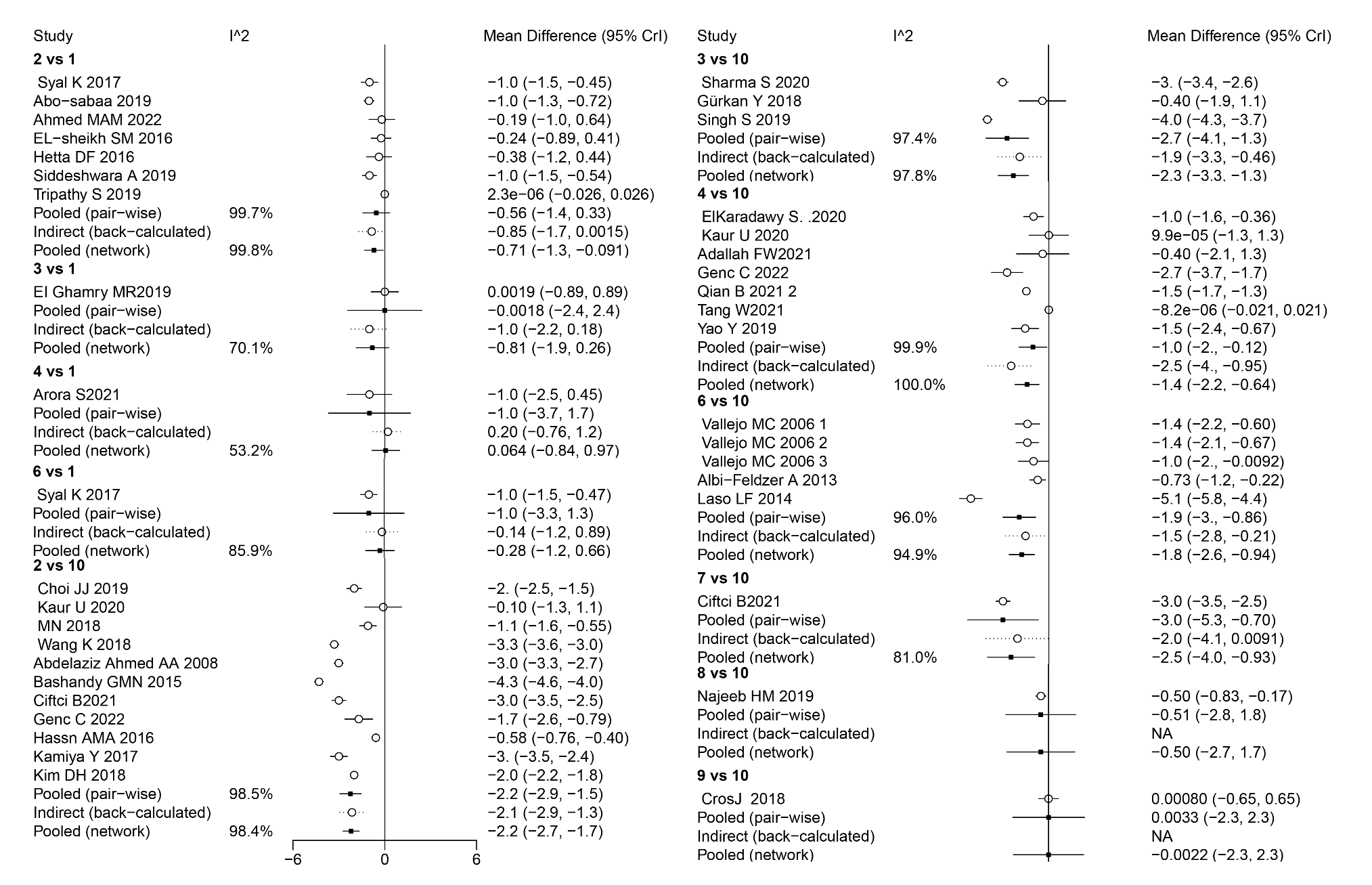
**

**Fiugre 1a. PACU pain scores**

**
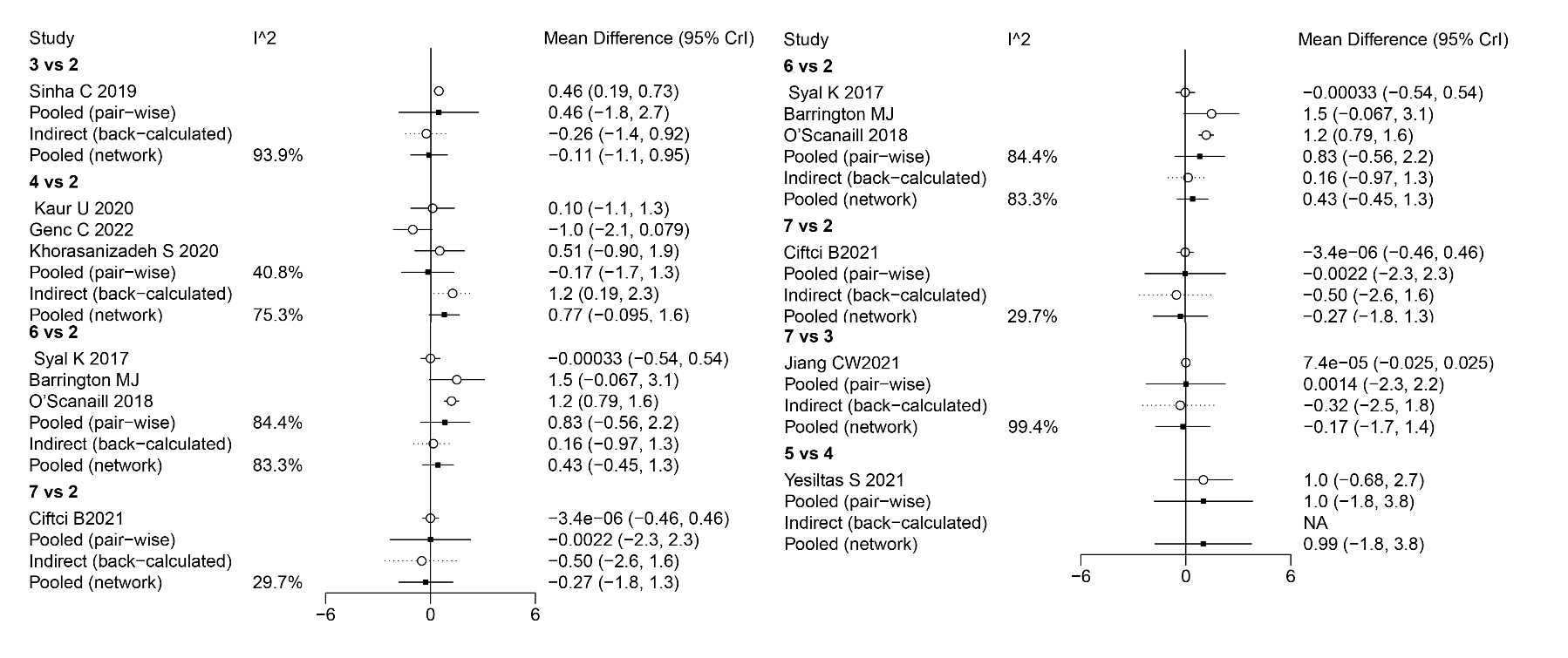
**

**Figure 1b. PACU pain scores**

**
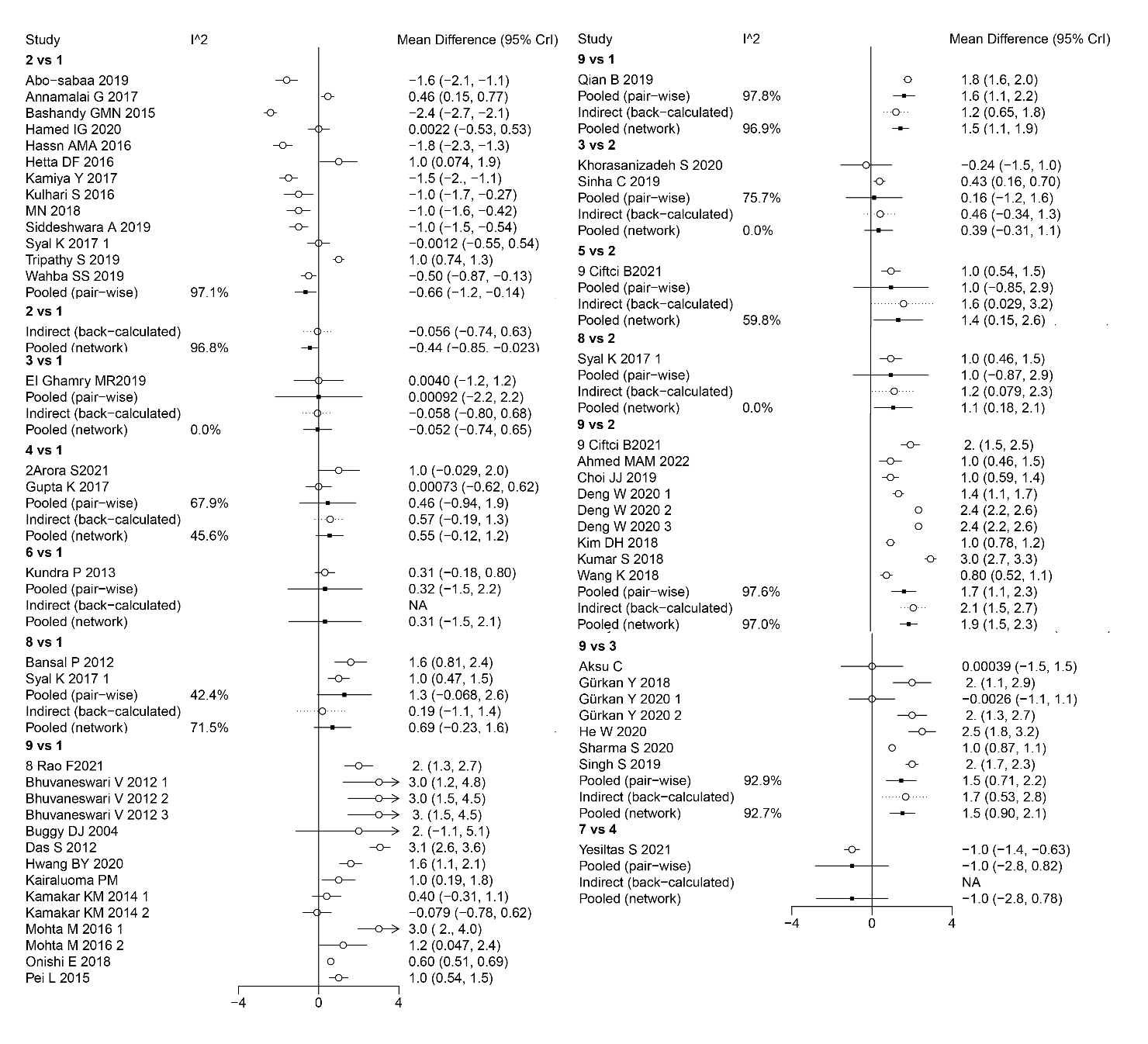
**

**Figure 2a. Postoperative 24h**

**
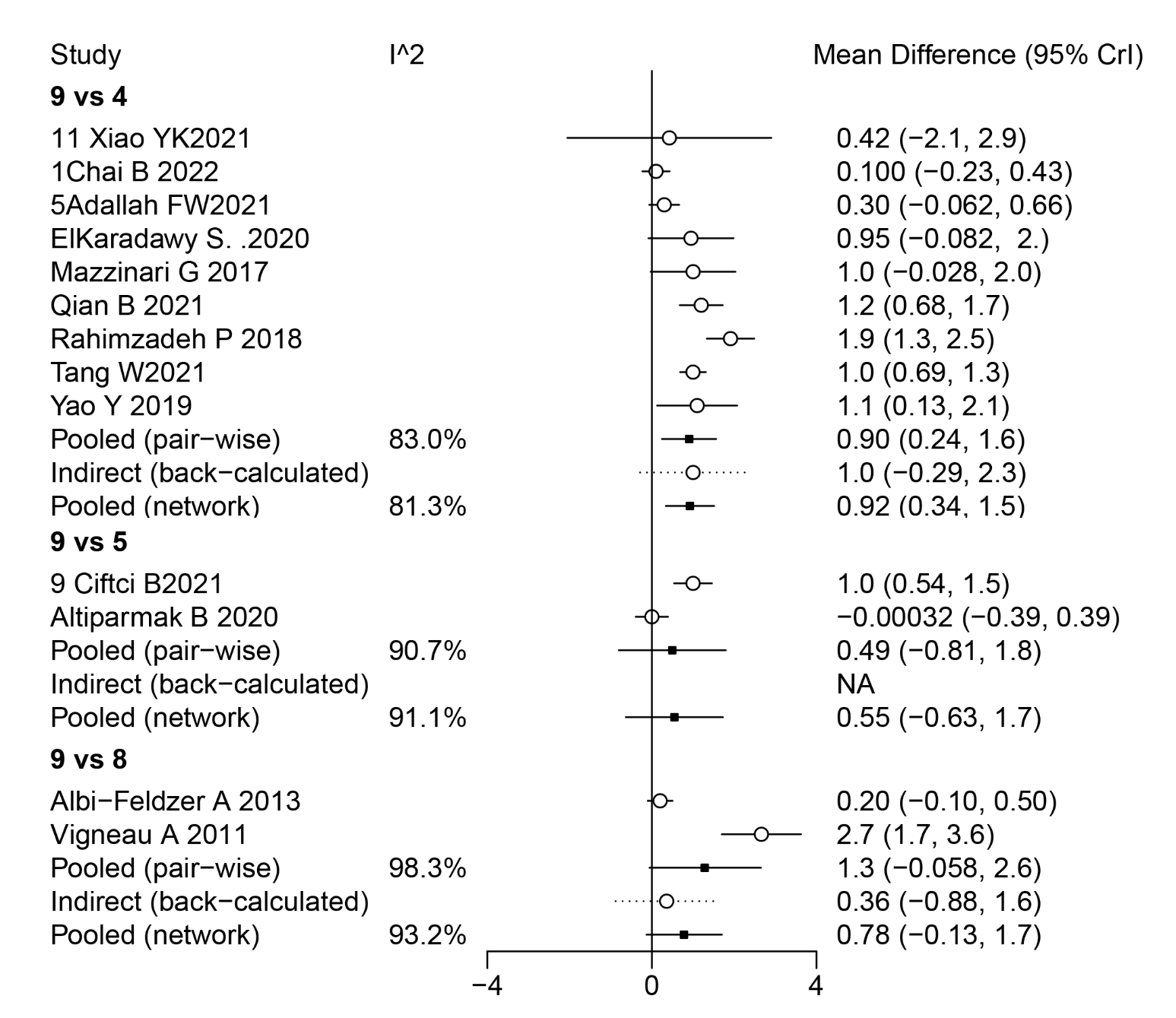
**

**Figure 2b. Postoperative 24h**

**
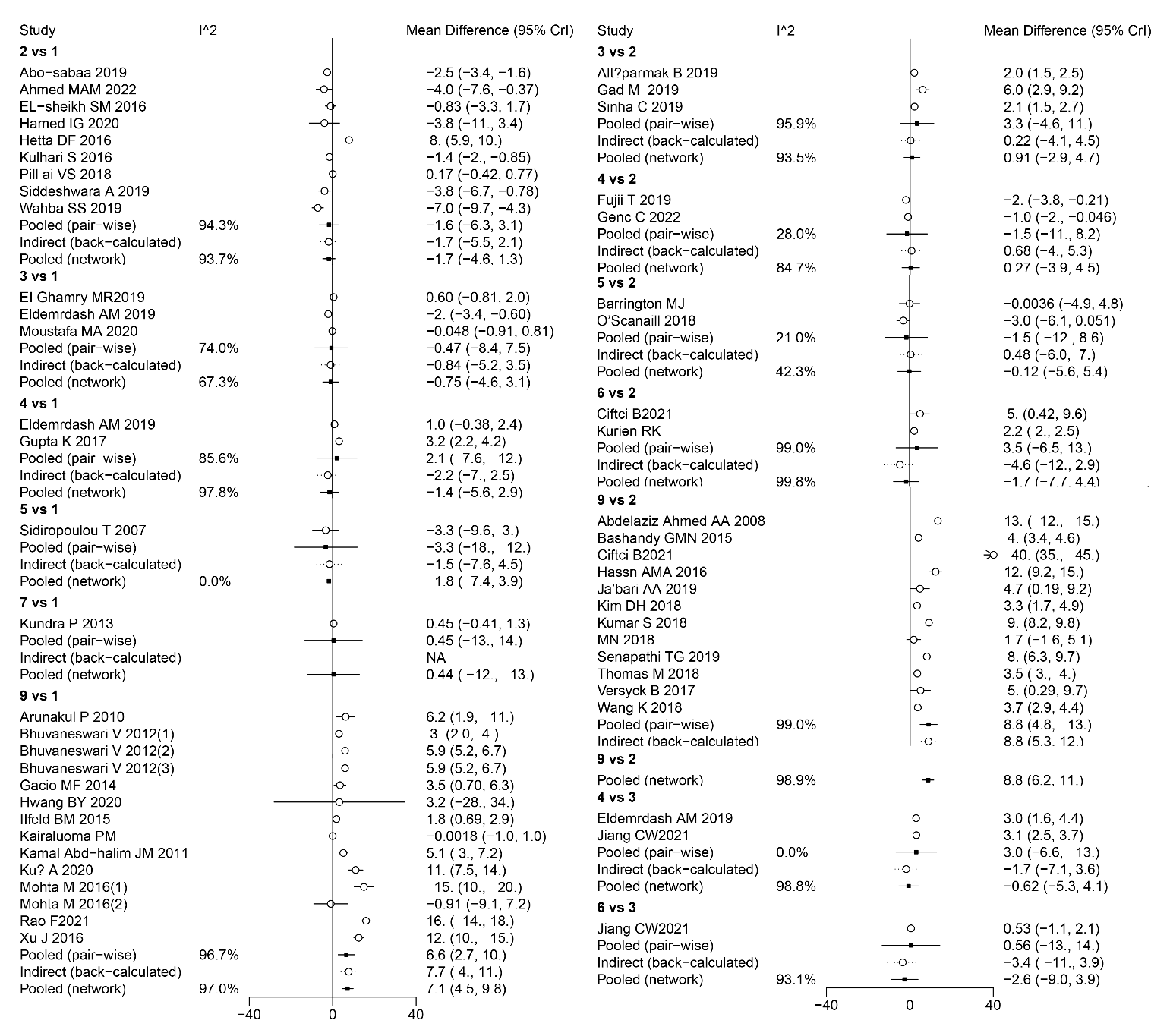
**

**Figure 3a. Postoperative 24h morphine consumption**

**
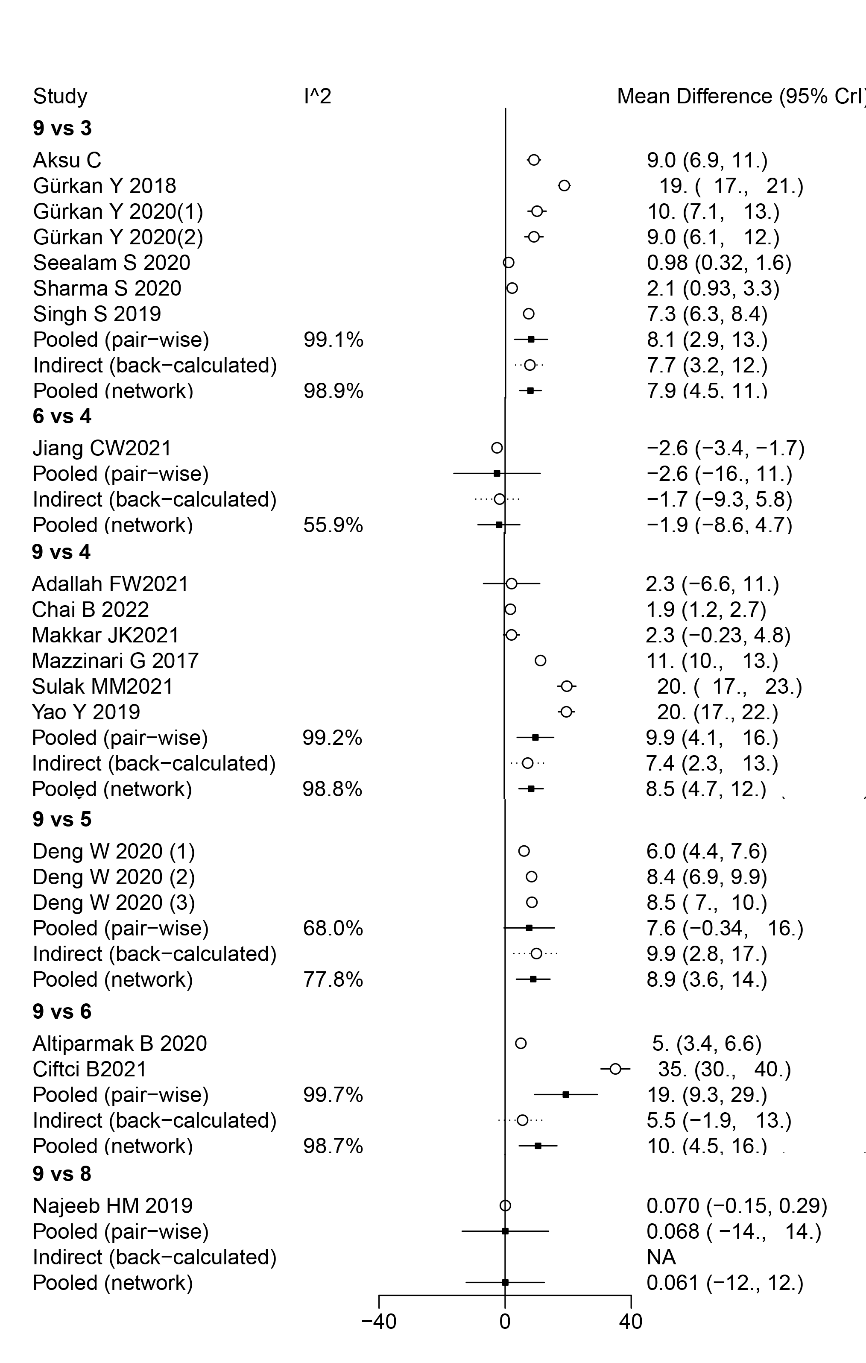
**

**Figure 3b. Postoperative 24h morphine consumption**

**
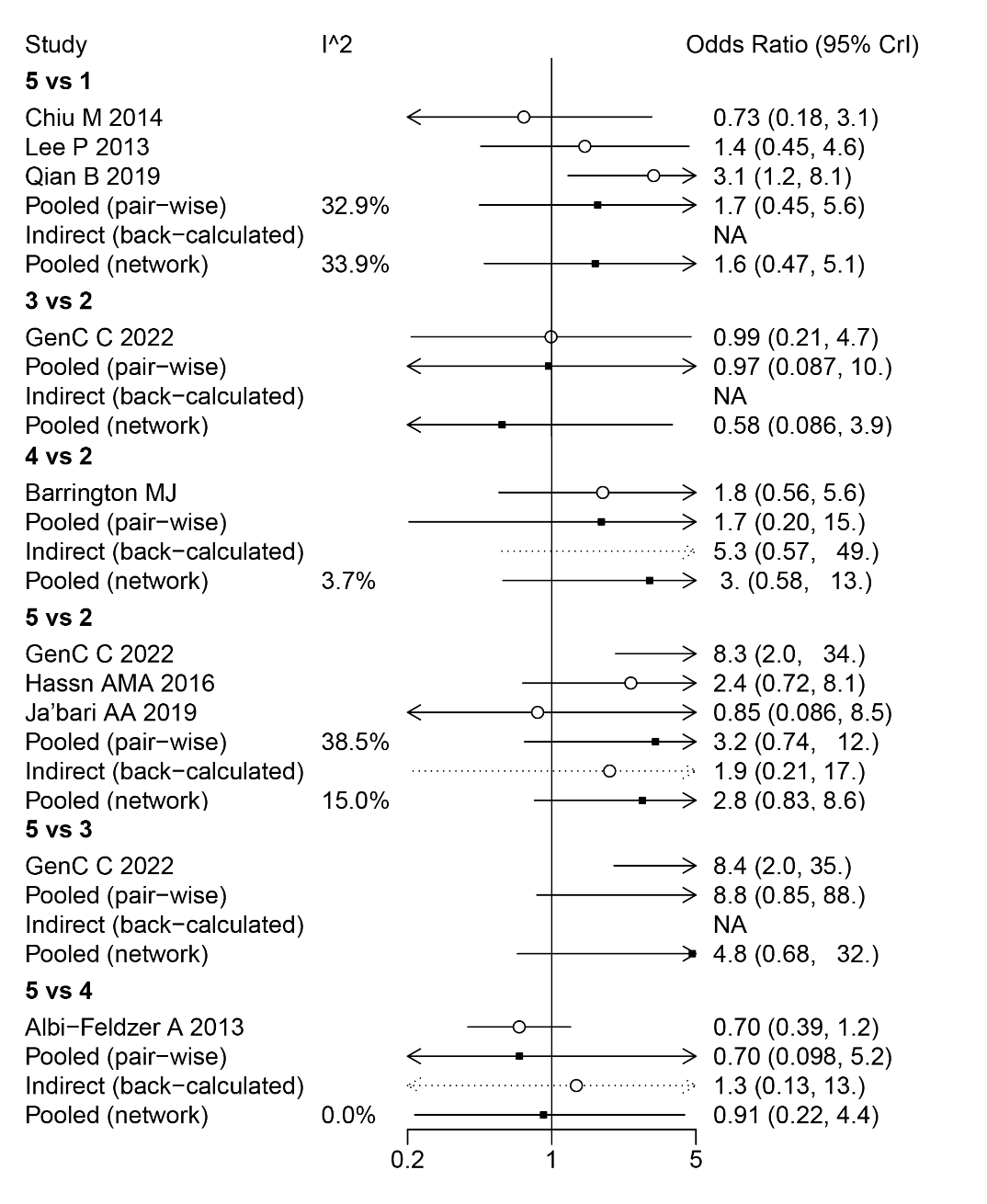
**

**Figure 4. Incidence of chronic pian**

**
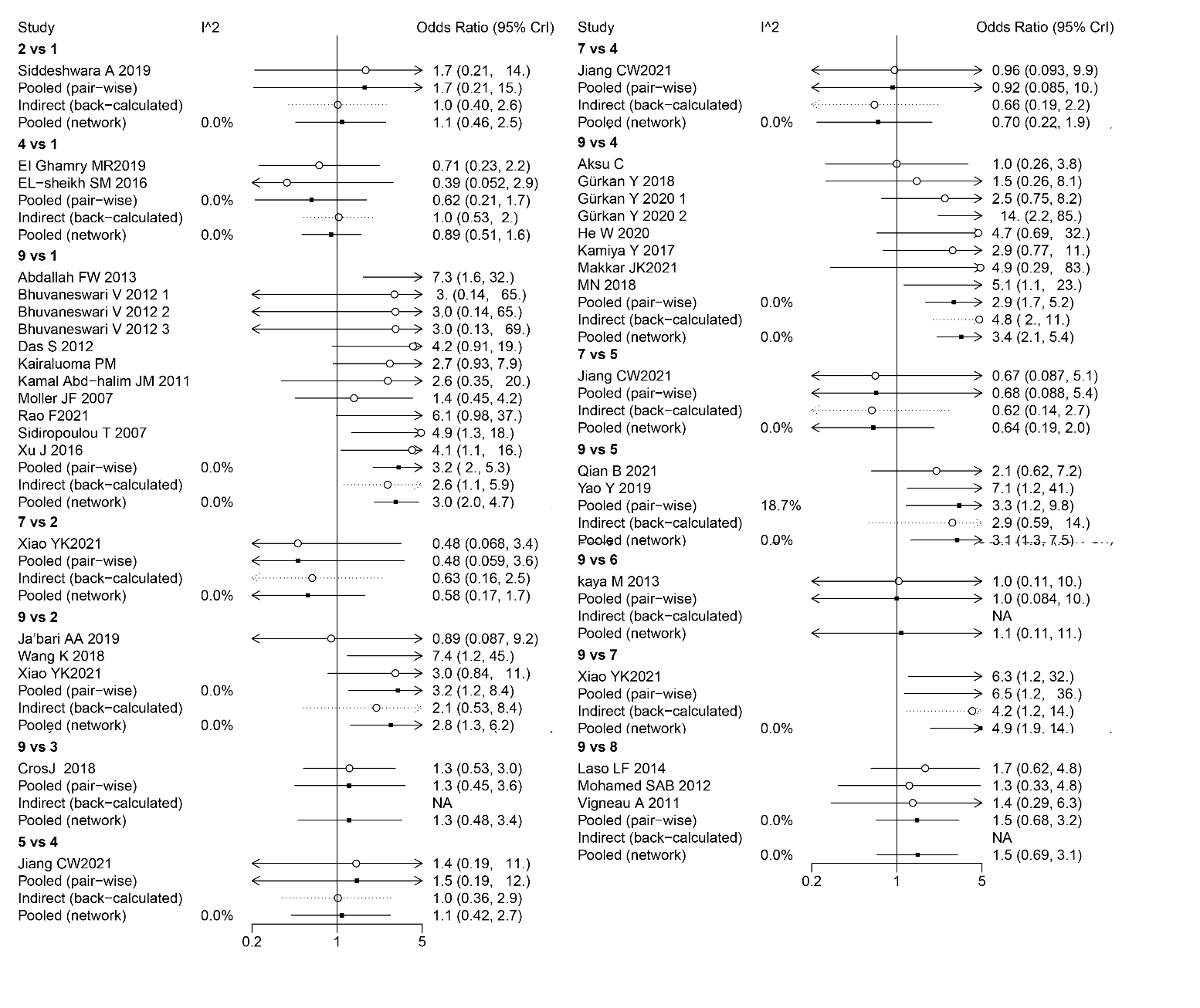
**

**Figure 5. Incidence of PONV**

**Appendix 12**

**Results of sensitivity analysis by only including trials with used of ultrasound**

**Treatment’s efficacy (league) tables**

Note: PECS-2 block: Pectoral nerve 2 block, PECS-1 block: Pectoral nerve 1 block, PVB: Paravertebral nerve block, ESPB: Erector spinae plane block, SPB: Serratus anterior plane block, RIB: Rhomboid intercostal block, IPB: Interpleural block, LA infusion: Local anesthetic infusion.

| **PVB** |  |  |  |  |  |  |  |  |  |
| --- | --- | --- | --- | --- | --- | --- | --- | --- | --- |
| 0.59 (-0.02, 1.19) | **PECS-2 block** |  |  |  |  |  |  |  |  |
| 0.78 (-0.26, 1.79) | 0.2 (-0.79, 1.15) | **ESPB** |  |  |  |  |  |  |  |
| 0.00 (-0.88, 0.88) | -0.59 (-1.4, 0.22) | -0.78 (-1.92, 0.39) | **SPB** |  |  |  |  |  |  |
| 0.75 (-1.00, 2.44) | 0.16 (-1.55, 1.80) | -0.03 (-1.93, 1.82) | 0.75 (-0.95, 2.38) | **SPB+PECS-1 block** |  |  |  |  |  |
| -0.14(-1.14, 0.83) | -0.73 (-1.64, 0.16) | -0.92 (-2.16, 0.33) | -0.14 (-1.26, 0.98) | -0.89 (-2.71, 0.98) | **LA infusion** |  |  |  |  |
| 0.92 (-0.56, 2.38) | 0.32(-1.09, 1.72) | 0.14 (-1.29, 1.59) | 0.92 (-0.66, 2.45) | 0.18 (-1.94, 2.33) | 1.05 (-0.57, 2.67) | **RIB** |  |  |  |
| -1.04 (-3.16, 1.08) | -1.62 (-3.73, 0.49) | -1.82 (-4.02, 0.47) | -1.03 (-3.20, 1.12) | -1.78 (-4.35, 0.82) | -0.9 (-3.11, 1.35) | -1.96 (-4.41, 0.52) | **SPB+PECS-2 block** |  |  |
| -1.53 (-3.72, 0.61) | -2.13 (-4.3, 0.01) | -2.31 (-4.63, -0.03) | -1.54 (-3.76, 0.66) | -2.29 (-4.94, 0.37) | -1.41 (-3.7, 0.87) | -2.46 (-5.03, 0.09) | -0.51 (-3.44, 2.37) | **PECS-1 block** |  |
| **-1.54 (-2.14, -0.93)** | **-2.13 (-2.65, -1.62)** | **-2.32 (-3.23, -1.39)** | **-1.54 (-2.25, -0.81)** | -2.28 (-3.88, 0.64) | **-1.4 (-2.3, -0.48)** | **-2.455 (-3.85, -1.05)** | -0.5 (-2.55, 1.52) | 0.00 (-2.08, 2.11) | **No block** |

**Table 1.** **MD with 95% CrI of network meta-analysis for PACU pain score**

**Table 2. MD with 95% CrI of network meta-analysis for postoperative 24 hours pain**

| **PVB** |  |  |  |  |  |  |  |
| --- | --- | --- | --- | --- | --- | --- | --- |
| -01 (-0.57, 0.39) | **PECS-2 block** |  |  |  |  |  |  |
| -0.09 (-0.82, 0.62) | -0.00 (-0.65, 0.66) | **ESPB** |  |  |  |  |  |
| -0.07 (-0.64, 0.51) | 0.03 (-0.49, 0.55) | 0.023 (-0.68, 0.74) | **SPB** |  |  |  |  |
| -0.48 (-1.84, 0.9) | -0.38 (-1.73, 0.96) | -0.38 (-1.82, 1.028) | -0.41 (-1.71, 0.9) | **LA infusion** |  |  |  |
| 0.11 (-0.66, 0.87) | 0.21 (-0.53, 0.93) | 0.21 (-0.71, 1.12) | 0.173 (-0.66, 1.00) | 0.59 (-0.90, 2.07) | **RIB** |  |  |
| -0.24 (-1.35, 0.89) | -0.14 (-1.21, 0.93) | -0.146 (-1.28, 0.99) | -0.17 (-1.25, 0.90) | 0.23 (-1.42, 1.87) | -0.36(-1.62, 0.91) | **IPB** |  |
| -0.94 (-1.4, -0.46) | -0.84 (-1.21, -0.48) | -0.84 (-1.44, -0.25) | -0.87 (-1.32, -0.43) | -0.46 (-1.76, 0.85) | -1.05(-1.78, -0.31) | -0.69 (-1.76, 0.34) | No block |

**Table 3. MD with 95% CrI of network meta-analysis for postoperative 24 morphine consumption**

| **PVB** |  |  |  |  |  |  |  |
| --- | --- | --- | --- | --- | --- | --- | --- |
| 1.50 (-1.72, 4.71) | **PECS-2 block** |  |  |  |  |  |  |
| 0.39 (-3.71, 4.47) | -1.11 (-5.02, 2.83) | **ESPB** |  |  |  |  |  |
| 0.93 (-3.53, 5.48) | -0.55 (-4.91, 3.76) | 0.54 (-4.22, 5.33) | **SPB** |  |  |  |  |
| 1.05 (-5.45, 7.62) | -0.42 (-6.47, 5.65) | 0.66 (-6.01, 7.51) | 0.15 (-6.82, 7.11) | **LA infusion** |  |  |  |
| 2.94 (-3.52, 9.50) | 1.46 (-4.62, 7.65) | 2.53 (-3.97, 9.23) | 2.012 (-4.69, 8.82) | 1.9 (-6.47, 9.88) | **RIB** |  |  |
| -7.72 (-20.77, 5.65) | -9.22 (-22.18, 4.21) | -8.17 (-21.24, 5.4) | -8.69 (-21.86, 4.92) | -8.78 (-22.73, 5.31) | -10.65 (-24.7, 3.68) | **SPB+PECS-2 block** |  |
| **-7.75 (-10.97, -4.58)** | **-9.25 (-12.06, -6.40)** | **-8.15 (-11.70, -4.57)** | **-8.69 (-12.72, -4.73)** | **-8.84 (-14.7, -3.02)** | **-10.70 (-16.80, -4.69)** | 0.00 (-12.85, 12.57) | **No block** |

**Table 4. OR with 95%CrI of network meta-analysis for incidence of chronic pain**

| **PVB** |  |  |  |  |
| --- | --- | --- | --- | --- |
| 0.48 (-1.2, 2.23) | **PECS-2 block** |  |  |  |
| 1.068 (-1.20, 3.44) | 0.57 (-1.34, 2.52) | **SPB** |  |  |
| -0.59 (-2.38, 1.54) | -1.07 (-2.54, 0.60) | -1.64 (-3.91, 0.77) | **LA infusion** |  |
| -0.51 (-1.67, 0.77) | -1.00 (-2.14, 0.25) | -1.58 (-3.47, 0.37) | 0.076 (-1.51, 1.53) | **No block** |

**Table 5. OR with 95%CI of network meta-analysis for PONV (postoperative 24-hour)**

| **PVB** |  |  |  |  |  |  |  |
| --- | --- | --- | --- | --- | --- | --- | --- |
| -0.17 (-1.13, 0.77) | **PECS-2 block** |  |  |  |  |  |  |
| -0.97 (-2.21, 0.26) | -0.80 (-2.13, 0.57) | **PECS-1 block** |  |  |  |  |  |
| 0.055 (-0.64, 0.72) | 0.22 (-0.73, 1.17) | 1.017 (-0.14, 2.20) | **ESPB** |  |  |  |  |
| -0.062 (-1.081, 0.99) | 0.125 (-1.08, 1.32) | 0.91 (-0.5, 2.35) | -0.11 (-1.07, 0.88) | **SPB** |  |  |  |
| -1.11 (-3.60, 1.31) | -0.93 (-3.48, 1.49) | -0.15 (-2.82, 2.41) | -1.15 (-3.65, 1.27) | -1.07 (-3.64, 1.44) | **IPB** |  |  |
| 0.44 (-0.72, 1.68) | 0.62 (-0.52, 1.87) | 1.43 (-0.06, 2.95) | 0.39 (-0.69, 1.59) | 0.50 (-0.72, 1.77) | 1.54 (-0.98, 4.29) | **RIB** |  |
| **-1.2 (-1.81, -0.62)** | **-1.03(-1.85, -0.20)** | -0.23 (-1.30, 0.83) | **-1.25 (-1.76, -0.76)** | **-1.14 (-2.06, -0.27)** | -0.09 (-2.43, 2.37) | **-1.65 (-2.748, -0.62)** | **No block** |

| **PACU pain scores** | | **Postoperative 24-hour pain scores** | | **Postoperative 24-hour morphine consumption** | | **PONV** | |
| --- | --- | --- | --- | --- | --- | --- | --- |
| **Treatment** | **SUCRA (%)** | **Treatment** | **SUCRA (%)** | **Treatment** | **SUCRA (%)** | **Treatment** | **SUCRA (%)** |
| RIB | 82.6 | RIB | 73.9 | RIB | 80.4 | RIB | 86.9 |
| ESPB | 81.5 | PVB | 66.9 | PECS-2 block | 70.4 | ESPB | 68.9 |
| PECS-2 block | 76.4 | SPB | 59.8 | LA infusion | 62.4 | PVB | 67.3 |
| SPB+PECS-1 block | 76 | ESPB | 56.9 | SPB | 62.3 | SPB | 61.9 |
| SPB | 48.1 | PECS-2 block | 55.9 | ESPB | 54.1 | PECS-2 block | 57 |
| PVB | 46.9 | IPB | 47.6 | PVB | 48.1 | IPB | 24.7 |
| LA infusion | 42.5 | LA infusion | 34.2 | SPB+PECS-2 block | 15.1 | PECS-1 block | 21.9 |
| SPB+PECS-2 block | 23.2 | No block | 4.8 | No block | 7.1 | No block | 11.5 |
| PECS-1 block | 13.7 | RIB | 73.9 | RIB | 80.4 | RIB | 86.9 |
| No block | 9 |  |  |  |  |  |  |

**Table 6. Cumulative ranking probability (SCURA)**

## Appendix 13

**Meta-regression for network meta-analyses on age and type of surgery** **compared with No block**

**Table 1. PACU pain**

| **Interventions** | **β(regression coefficient)** | **95%CI** |
| --- | --- | --- |
| No block vs PVB | a:0.49  b: -0.11 | a:(-3.96, 2.31)  b:( -1.54, 1.30) |
| No block vs PECS-2 block | a: -1.12  b: 0.49 | a:(-12.08, 4.07)  b:( -0.71, 4.62) |
| No block vs ESPB | a: 1.43  b: -6.15 | a: (-2.30, 4.85)  b:(-28.67, 18.99) |
| No block vs SPB | a:0.112  b:1.26 | a:(-6.27,2.65)  b:(-1.47, 6.31) |
| No block vs LA infusion | a: -1.55  b: 3.02135 | a:( -43.52, 28.92)  b:( -43.17, 91.30) |
| No block vs RIB | a:0.24  b: 2.13 | a:(-8.11, 3.91)  b:( -0.38, 5.11) |
| No block vs SPB+PECS-2 block | a: -2.74  b: 0.46 | a:(-16.62, 4.09)  b:(-6.35, 12.15) |
| No block vs PECS-1 block | a:2.20  b:0.36 | a:(-29.05, 43.46)  b:(26.77, 26.30) |
| SPB VS SPB+PECS-1 block | a:1.84  b:3.13 | a:(-17.21, 34.15)  a:(-64.39, 114.53) |

Notes: 1: PVB, 2: PECS-2 block, 3: ESPB, 4: SPB, 5: SPB+PECS-1 block 6: LA infusion, 7: RIB, 8: SPB+PECS-2 block, 9: PECS-1 block, 10: No block, a: Age is concomitant variable, b: type of breast cancer surgery is concomitant variable

**Table 2. Postoperative 24-hours pain scores**

| **Interventions** | **β(regression coefficient)** | **95%CI** |
| --- | --- | --- |
| PVB VS PECS-2 block | a:0.31  b:0.40 | a: (-0.623,1.2545)  b: (-0.5386 1.31) |
| PVB VS ESPB | a:0.12  b:0.45 | a:( -0.54, 0.85)  b: (-0.79, 1.68) |
| PVB VS SPB | a:0.29  b: -0.27 | a:(-1.35, 2.06)  b:( -2.98, 2.13) |
| PVB VS LA infusion | a: -0.30  b:0.12 | a:(-1.34, 0.75)  b:(-0.71, 0.93) |
| PVB VS IPB | a: 0.96  b: 1.32 | a:(-1.56, 3.54)  b:(-1.93, 4.80) |
| PVB VS No block | a: -0.96  b: -0.20 | a:(-2.06, 0.15)  b: (-1.13, 0.69) |
| SPB VS SPB+PECS-1 block | a: -0.42  b: -0.32 | a:(-2.69, 1.32)  b:(-12.84, 4.67) |
| SPB VS RIB | a:0.81  b: -1.61 | a:(-23.12, 29.91)  b:(-23.66, 11.97) |

Notes: 1: PVB, 2: PECS-2 block, 3: ESPB, 4: SPB, 5: SPB+PECS-1 block, 6: LA infusion, 7: RIB, 8: IPB, 9: No block, a: Age is concomitant variable, b: type of breast cancer surgery is concomitant variable

**Table 3. Postoperative 24-hours morphine consumption**

| **Interventions** | **β(regression coefficient)** | **95%CI** |
| --- | --- | --- |
| PVB VS PECS-2 block | a: 2.58  b: -0.18 | a:(-4.37, 9.65)  b:( -7.46, 23.20) |
| PVB VS ESPB | a:5.54  b: -4.50 | a:(-0.06, 11.10)  b:(-12.95, 9.01) |
| PVB VS SPB | a:2.22  b: -1.4716 | a:(-6.67, 11.33)  b: (-9.959, 18.123) |
| PVB VS SPB+PECS-1 block | a:5.29  b: -0.43 | a:(-2.94, 13.5)  b:( -6.44, 10.01) |
| PVB VS LA infusion | a:1.41  b: 2.18 | a:(-7.91, 10.8)  b:( -32.81, 176.52) |
| d.1.9 | a: 13.39  b:2.18 | a:(-0.45, 27.66)  b:(-32.81, 176.52) |
| d.9.6 | a: -3.00  b: -16.94 | a: (-353.50, 336.35)  b:(-255.46, 103.573) |
| d.9.8 | a:56.80  b: 36.52 | a:( -87.25, 369.39)  b:(-53.77, 129.75) |

Notes: 1: PVB, 2: PECS-2 block, 3: ESPB, 4: SPB, 5: SPB+PECS-1 block, 6: SPB+PECS-2 block, 7: LA infusion, 8: RIB, 9: IPB; 10: No block, a: Age is concomitant variable, b: type of breast cancer surgery is concomitant variable

**Table 4. Incidence of chronic pian**

| **Interventions** | **β(regression coefficient)** | **95%CI** |
| --- | --- | --- |
| No block VS PVB | a:0.90  b: 0.79 | a:(-1.63, 3.56)  b:(-1.33, 2.87) |
| No block VS PECS-2 block | a:0.42  b: 0.96 | a:(-1.53, 2.42)  b:(-6.84, 16.09) |
| No block VS SPB | a:0.06  b: -0.05 | a:(-16.97, 18.00)  b:(-19.09,18.21) |
| No block VS LA infusion | a: -0.70  b: 0.70 | a:(-9.11, 5.69)  b:(-1.69, 3.08) |

Notes: 1: PVB, 2: PECS-2 block, 3: SPB, 4 LA infusion, 5: No block, a: Age is concomitant variable, b: type of breast cancer surgery is concomitant variable

**Table 5. Incidence of PONV**

| **Interventions** | **β (regression coefficient)** | **95%CI** |
| --- | --- | --- |
| No block VS PVB | a:0.01  b:0.73 | a: (-0.78, 0.80)  b: (-0.38, 1.87) |
| No block VS PECS-2 block | a:0.52  b:0.00 | a:(-1.32, 2.48)  b:( -1.88, 1.85) |
| No block VS PECS-1 block | a:0.00  b:6.46 | a:(-10.18, 12.66)  b:(-19.82, 88.36) |
| No block VS ESPB | a:0.38  b: 0.59 | a (-0.95,1.74)  b:( -0.78, 1.96) |
| No block VS SPB | a:1.01  b: -0.67 | a:(-1.58, 4.08)  b: -29.04, 16.14) |
| No block VS IPB | a:0.66  b:1.02 | a:(-15.91, 21.96)  b:( -12.98, 25.64) |
| No block VS RIB | a: -1.32  b: -0.29 | a:(-19.58, 7.33)  b:(-2.68, 2.04) |
| No block VS LA infusion | a: -0.13  b: 0.04 | a:(-1.21, 0.94)  b:( -0.75, 0.83) |

Notes: 1: PVB, 2: PECS-2 block, 3: PECS-1 block, 4: ESPB, 5: SPB, 6: IPB, 7: RIB, 8: LA infusion, 9: No block. a: Age is concomitant variable, b: type of breast cancer surgery is concomitant variable

## Appendix 14

## Evaluation of the quality of evidence using Cochrane Collaboration's tool and Confidence in Network Meta-analysis (CINeMA 0.6.1 version)

**Table 1. PACU pain scores**

| **Comparison** | **Number of studies** | | **Within-study bias** | **Reporting bias** | **Indirectness** | **Imprecision** | **Heterogeneity** | **Incoherence** | **Confidence rating** | **Reason(s) for downgrading** |
| --- | --- | --- | --- | --- | --- | --- | --- | --- | --- | --- |
| ESPB:PECS-2 block | | 2 | Some concerns | Low risk | No concerns | Some concerns | Some concerns | No concerns | Moderate |  |
| ESPB:PVB | | 1 | Some concerns | Low risk | No concerns | Some concerns | Some concerns | No concerns | Moderate |  |
| ESPB:no block | | 3 | Some concerns | Low risk | No concerns | No concerns | Some concerns | No concerns | Moderate |  |
| LA infusion:PECS-2 block | | 3 | No concerns | Low risk | No concerns | Some concerns | Some concerns | No concerns | Moderate |  |
| LA infusion:PVB | | 2 | Some concerns | Low risk | No concerns | Some concerns | Some concerns | No concerns | Moderate |  |
| LA infusion:no block | | 2 | Some concerns | Low risk | No concerns | No concerns | Some concerns | No concerns | Moderate |  |
| no block: PECS-1 block | | 1 | No concerns | Low risk | No concerns | Major concerns | No concerns | No concerns | High |  |
| PECS-2 block:PVB | | 4 | Some concerns | Low risk | No concerns | No concerns | Major concerns | No concerns | High |  |
| PECS-2 block:RIB | | 1 | Major concerns | Low risk | No concerns | Major concerns | No concerns | No concerns | High |  |
| PECS-2 block:SPB | | 1 | Some concerns | Low risk | No concerns | Some concerns | Some concerns | No concerns | Moderate |  |
| no block:PECS-2 block | | 12 | Some concerns | Low risk | No concerns | No concerns | No concerns | No concerns | Moderate |  |
| PVB:SPB | | 1 | Some concerns | Low risk | No concerns | Some concerns | Some concerns | No concerns | Moderate |  |
| no block:PVB | | 11 | Some concerns | Low risk | No concerns | No concerns | Some concerns | No concerns | High |  |
| no block:RIB | | 1 | Major concerns | Low risk | No concerns | No concerns | Some concerns | No concerns | Moderate |  |
| SPB:SPB+PECS-1 block | | 1 | Some concerns | Low risk | No concerns | Major concerns | No concerns | No concerns | Moderate |  |
| no block:SPB | | 6 | Some concerns | Low risk | No concerns | No concerns | Some concerns | Some concerns | High |  |
| no block:SPB+PECS-2 block | | 1 | Major concerns | Low risk | No concerns | Major concerns | No concerns | No concerns | Very low | ["Within-study bias","Imprecision"] |
| ESPB:LA infusion | | 0 | No concerns | Low risk | No concerns | Some concerns | Some concerns | No concerns | High |  |
| ESPB:PECS-1 block | | 0 | No concerns | Low risk | No concerns | Some concerns | Some concerns | No concerns | High |  |
| ESPB:RIB | | 0 | Some concerns | Low risk | No concerns | Major concerns | No concerns | No concerns | Low | ["Imprecision"] |
| ESPB:SPB | | 0 | Some concerns | Low risk | No concerns | Some concerns | Some concerns | No concerns | High |  |
| ESPB:SPB+PECS-1 block | | 0 | Some concerns | Low risk | No concerns | Major concerns | No concerns | No concerns | Low | ["Imprecision"] |
| ESPB:SPB+PECS-2 block | | 0 | Some concerns | Low risk | No concerns | Some concerns | Some concerns | No concerns | High |  |
| LA infusion:PECS-1 block | | 0 | No concerns | Low risk | No concerns | Some concerns | Some concerns | No concerns | High |  |
| LA infusion:RIB | | 0 | Some concerns | Low risk | No concerns | Some concerns | Some concerns | No concerns | High |  |
| LA infusion:SPB | | 0 | Some concerns | Low risk | No concerns | Major concerns | No concerns | No concerns | Low | ["Imprecision"] |
| LA infusion:SPB+PECS-1 block | | 0 | Some concerns | Low risk | No concerns | Major concerns | No concerns | No concerns | Low | ["Imprecision"] |
| LA infusion:SPB+PECS-2 block | | 0 | Some concerns | Low risk | No concerns | Major concerns | No concerns | No concerns | Low | ["Imprecision"] |
| PECS-1 block:PECS-2 block | | 0 | Some concerns | Low risk | No concerns | No concerns | Some concerns | No concerns | High |  |
| PECS-1 block:PVB | | 0 | Some concerns | Low risk | No concerns | Some concerns | Some concerns | No concerns | High |  |
| PECS-1 block:RIB | | 0 | Some concerns | Low risk | No concerns | Some concerns | No concerns | No concerns | High |  |
| PECS-1 block:SPB | | 0 | Some concerns | Low risk | No concerns | Some concerns | Some concerns | No concerns | High |  |
| PECS-1 block:SPB+PECS-1 block | | 0 | Some concerns | Low risk | No concerns | Major concerns | No concerns | No concerns | Low | ["Imprecision"] |
| PECS-1 block:SPB+PECS-2 block | | 0 | Some concerns | Low risk | No concerns | Major concerns | No concerns | No concerns | Low | ["Imprecision"] |
| PECS-2 block:SPB+PECS-1 block | | 0 | Some concerns | Low risk | No concerns | Major concerns | No concerns | No concerns | Low | ["Imprecision"] |
| PECS-2 block:SPB+PECS-2 block | | 0 | Major concerns | Low risk | No concerns | Some concerns | Some concerns | No concerns | Low | ["Within-study bias"] |
| PVB:RIB | | 0 | Some concerns | Low risk | No concerns | Some concerns | Some concerns | No concerns | High |  |
| PVB:SPB+PECS-1 block | | 0 | Some concerns | Low risk | No concerns | Major concerns | No concerns | No concerns | Low | ["Imprecision"] |
| PVB:SPB+PECS-2 block | | 0 | Some concerns | Low risk | No concerns | Major concerns | No concerns | No concerns | Low | ["Imprecision"] |
| RIB:SPB | | 0 | Major concerns | Low risk | No concerns | Some concerns | Some concerns | No concerns | Low | ["Within-study bias"] |
| RIB:SPB+PECS-1 block | | 0 | Some concerns | Low risk | No concerns | Major concerns | No concerns | No concerns | Low | ["Imprecision"] |
| RIB:SPB+PECS-2 block | | 0 | Major concerns | Low risk | No concerns | Some concerns | Some concerns | No concerns | Low | ["Within-study bias"] |
| SPB:SPB+PECS-2 block | | 0 | Major concerns | Low risk | No concerns | Major concerns | No concerns | No concerns | Very low | ["Within-study bias","Imprecision"] |
| SPB+PECS-1 block:SPB+PECS-2 block | | 0 | Some concerns | Low risk | No concerns | Major concerns | No concerns | No concerns | Low | ["Imprecision"] |
| no block:SPB+PECS-1 block | | 0 | Some concerns | Low risk | No concerns | Major concerns | No concerns | No concerns | Low | ["Imprecision"] |

**Table 2. Postoperative 24-hour pain scores**

| **Comparison** | **Number of studies** | **Within-study bias** | **Reporting bias** | **Indirectness** | **Imprecision** | **Heterogeneity** | **Incoherence** | **Confidence rating** | **Reason(s) for downgrading** |
| --- | --- | --- | --- | --- | --- | --- | --- | --- | --- |
| ESPB:PECS-2 block | 1 | Some concerns | Low risk | No concerns | No concerns | Major concerns | No concerns | Low | ["Heterogeneity"] |
| ESPB:PVB | 1 | Some concerns | Low risk | No concerns | No concerns | Major concerns | No concerns | Low | ["Heterogeneity"] |
| ESPB:RIB | 1 | Some concerns | Low risk | No concerns | No concerns | Major concerns | No concerns | Low | ["Heterogeneity"] |
| ESPB:SPB | 1 | Some concerns | Low risk | No concerns | No concerns | Major concerns | No concerns | Low | ["Heterogeneity"] |
| ESPB:no block | 7 | Some concerns | Low risk | No concerns | No concerns | Some concerns | No concerns | Moderate |  |
| IPB:PVB | 1 | Major concerns | Low risk | No concerns | Major concerns | No concerns | No concerns | Very low | ["Within-study bias","Imprecision"] |
| IPB:no block | 1 | Major concerns | Low risk | No concerns | Some concerns | Some concerns | No concerns | Low | ["Within-study bias"] |
| LA infusion: PECS-2 block | 3 | Some concerns | Low risk | No concerns | No concerns | Major concerns | No concerns | Low | ["Heterogeneity"] |
| LA infusion:PVB | 3 | Some concerns | Low risk | No concerns | No concerns | Major concerns | Major concerns | Very low | ["Heterogeneity","Incoherence"] |
| LA infusion:no block | 4 | Some concerns | Low risk | No concerns | No concerns | Some concerns | No concerns | High |  |
| PECS-2 block:PVB | 6 | Some concerns | Low risk | No concerns | No concerns | Major concerns | No concerns | Low | ["Heterogeneity"] |
| PECS-2 block:RIB | 2 | Major concerns | Low risk | No concerns | No concerns | Major concerns | No concerns | Very low | ["Within-study bias","Heterogeneity"] |
| PECS-2 block:SPB | 2 | Some concerns | Low risk | No concerns | No concerns | Major concerns | No concerns | Low | ["Heterogeneity"] |
| no block:PECS-2 block | 17 | Some concerns | Low risk | No concerns | No concerns | Some concerns | No concerns | Moderate |  |
| PVB:SPB | 3 | Some concerns | Low risk | No concerns | No concerns | Major concerns | No concerns | Low | ["Heterogeneity"] |
| no block:PVB | 16 | Some concerns | Low risk | No concerns | No concerns | Some concerns | Some concerns | Moderate |  |
| RIB:SPB | 1 | Major concerns | Low risk | No concerns | No concerns | Major concerns | No concerns | Very low | ["Within-study bias","Heterogeneity"] |
| SPB:SPB+PECS-1 block | 1 | Some concerns | Low risk | No concerns | Some concerns | Some concerns | No concerns | Moderate |  |
| no block:SPB | 11 | Some concerns | Low risk | No concerns | No concerns | Some concerns | No concerns | Moderate |  |
| no block:SPB+PECS-1 block | 1 | Some concerns | Low risk | No concerns | Some concerns | Some concerns | No concerns | Moderate |  |
| ESPB:IPB | 0 | Some concerns | Low risk | No concerns | Some concerns | Some concerns | No concerns | Moderate |  |
| ESPB:LA infusion | 0 | Some concerns | Low risk | No concerns | No concerns | Major concerns | No concerns | Low | ["Heterogeneity"] |
| ESPB:SPB+PECS-1 block | 0 | Some concerns | Low risk | No concerns | Some concerns | Some concerns | No concerns | Moderate |  |
| IPB:LA infusion | 0 | Some concerns | Low risk | No concerns | Major concerns | No concerns | No concerns | Low | ["Imprecision"] |
| IPB:PECS-2 block | 0 | Major concerns | Low risk | No concerns | Some concerns | Some concerns | No concerns | High | ["Within-study bias"] |
| IPB:RIB | 0 | Major concerns | Low risk | No concerns | Major concerns | No concerns | No concerns | Very low | ["Within-study bias","Imprecision"] |
| IPB:SPB | 0 | Some concerns | Low risk | No concerns | Some concerns | Some concerns | No concerns | Moderate |  |
| IPB:SPB+PECS-1 block | 0 | Some concerns | Low risk | No concerns | Major concerns | No concerns | No concerns | Low | ["Imprecision"] |
| LA infusion:RIB | 0 | Some concerns | Low risk | No concerns | Some concerns | Some concerns | No concerns | Moderate |  |
| LA infusion:SPB | 0 | Some concerns | Low risk | No concerns | No concerns | Major concerns | No concerns | Low | ["Heterogeneity"] |
| LA infusion:SPB+PECS-1 block | 0 | Some concerns | Low risk | No concerns | Some concerns | Some concerns | No concerns | Moderate |  |
| PECS-2 block:SPB+PECS-1 block | 0 | Some concerns | Low risk | No concerns | Some concerns | Some concerns | No concerns | Moderate |  |
| PVB:RIB | 0 | Some concerns | Low risk | No concerns | Some concerns | Some concerns | No concerns | Moderate |  |
| PVB:SPB+PECS-1 block | 0 | Some concerns | Low risk | No concerns | Some concerns | Some concerns | No concerns | Moderate |  |
| RIB:SPB+PECS-1 block | 0 | Some concerns | Low risk | No concerns | Some concerns | Some concerns | No concerns | Moderate |  |
| no block:RIB | 0 | Some concerns | Low risk | No concerns | No concerns | Some concerns | No concerns | Moderate |  |

**Table 3. Postoperative 24-hour morphine consumption**

| **Comparison** | **Number of studies** | **Within-study bias** | **Reporting bias** | **Indirectness** | **Imprecision** | **Heterogeneity** | **Incoherence** | **Confidence rating** | **Reason(s) for downgrading** |
| --- | --- | --- | --- | --- | --- | --- | --- | --- | --- |
| ESPB:PECS-2 block | 3 | Some concerns | Low risk | No concerns | Major concerns | No concerns | No concerns | Low | ["Imprecision"] |
| ESPB:PVB | 3 | Some concerns | Low risk | No concerns | Major concerns | No concerns | No concerns | Low | ["Imprecision"] |
| ESPB:RIB | 1 | Some concerns | Low risk | No concerns | No concerns | Major concerns | No concerns | Low | ["Heterogeneity"] |
| ESPB:SPB | 2 | Some concerns | Low risk | No concerns | Major concerns | No concerns | No concerns | Low | ["Imprecision"] |
| ESPB:no block | 7 | Some concerns | Low risk | No concerns | No concerns | No concerns | No concerns | High |  |
| IPB:PVB | 1 | Major concerns | Low risk | No concerns | Major concerns | No concerns | Major concerns | Very low | ["Within-study bias","Imprecision","Incoherence"] |
| LA infusion:PECS-2 block | 2 | No concerns | Low risk | No concerns | Major concerns | No concerns | No concerns | Low | ["Imprecision"] |
| LA infusion:PVB | 1 | Some concerns | Low risk | No concerns | Major concerns | No concerns | No concerns | Very low | ["Imprecision"] |
| PECS-2 block:PVB | 8 | Some concerns | Low risk | No concerns | Major concerns | No concerns | No concerns | Very low | ["Imprecision"] |
| PECS-2 block:RIB | 1 | Some concerns | Low risk | No concerns | Major concerns | No concerns | Major concerns | Very low | ["Imprecision","Incoherence"] |
| PECS-2 block:SPB | 2 | Some concerns | Low risk | No concerns | Major concerns | No concerns | No concerns | Low | ["Imprecision"] |
| no block:PECS-2 block | 18 | Some concerns | Low risk | No concerns | No concerns | No concerns | No concerns | High |  |
| PVB:SPB | 2 | Some concerns | Low risk | No concerns | Major concerns | No concerns | No concerns | Low | ["Imprecision"] |
| no block:PVB | 14 | Some concerns | Low risk | No concerns | No concerns | Major concerns | No concerns | Very low | ["Heterogeneity"] |
| RIB:SPB | 1 | Some concerns | Low risk | No concerns | Major concerns | No concerns | No concerns | Low | ["Imprecision"] |
| no block:RIB | 2 | Some concerns | Low risk | No concerns | No concerns | No concerns | No concerns | High |  |
| no block:SPB | 6 | Some concerns | Low risk | No concerns | No concerns | No concerns | No concerns | High |  |
| no block:SPB+PECS-2 block | 1 | Major concerns | Low risk | No concerns | Major concerns | No concerns | Major concerns | Very low | ["Within-study bias","Imprecision","Incoherence"] |
| ESPB:IPB | 0 | Some concerns | Low risk | No concerns | Major concerns | No concerns | Major concerns | Very low | ["Imprecision","Incoherence"] |
| ESPB:LA infusion | 0 | Some concerns | Low risk | No concerns | Major concerns | No concerns | Major concerns | Very low | ["Imprecision","Incoherence"] |
| ESPB:SPB+PECS-2 block | 0 | Some concerns | Low risk | No concerns | No concerns | Major concerns | Major concerns | Very low | ["Heterogeneity","Incoherence"] |
| IPB:LA infusion | 0 | Some concerns | Low risk | No concerns | Major concerns | No concerns | Major concerns | Very low | ["Imprecision","Incoherence"] |
| IPB:PECS-2 block | 0 | Some concerns | Low risk | No concerns | Major concerns | No concerns | Major concerns | Very low | ["Imprecision","Incoherence"] |
| IPB:RIB | 0 | Some concerns | Low risk | No concerns | Major concerns | No concerns | Major concerns | Very low | ["Imprecision","Incoherence"] |
| IPB:SPB | 0 | Some concerns | Low risk | No concerns | Major concerns | No concerns | Major concerns | Very low | ["Imprecision","Incoherence"] |
| IPB:SPB+PECS-2 block | 0 | Major concerns | Low risk | No concerns | Major concerns | No concerns | Major concerns | Very low | ["Within-study bias","Imprecision","Incoherence"] |
| IPB:no block | 0 | Some concerns | Low risk | No concerns | Major concerns | No concerns | Major concerns | Very low | ["Imprecision","Incoherence"] |
| LA infusion:RIB | 0 | Some concerns | Low risk | No concerns | Major concerns | No concerns | Major concerns | Very low | ["Imprecision","Incoherence"] |
| LA infusion:SPB | 0 | Some concerns | Low risk | No concerns | Major concerns | No concerns | Major concerns | Very low | ["Imprecision","Incoherence"] |
| LA infusion:SPB+PECS-2 block | 0 | Some concerns | Low risk | No concerns | No concerns | Major concerns | Major concerns | Very low | ["Heterogeneity","Incoherence"] |
| LA infusion:no block | 0 | Some concerns | Low risk | No concerns | No concerns | No concerns | Major concerns | Low | ["Incoherence"] |
| PECS-2 block:SPB+PECS-2 block | 0 | Major concerns | Low risk | No concerns | No concerns | Major concerns | Major concerns | Very low | ["Within-study bias","Heterogeneity","Incoherence"] |
| PVB:RIB | 0 | Some concerns | Low risk | No concerns | No concerns | Major concerns | Major concerns | Low | ["Heterogeneity","Incoherence"] |
| PVB:SPB+PECS-2 block | 0 | Some concerns | Low risk | No concerns | Major concerns | No concerns | Major concerns | Very low | ["Imprecision","Incoherence"] |
| RIB:SPB+PECS-2 block | 0 | Major concerns | Low risk | No concerns | No concerns | No concerns | Major concerns | Very low | ["Within-study bias","Incoherence"] |
| SPB:SPB+PECS-2 block | 0 | Some concerns | Low risk | No concerns | No concerns | Major concerns | Major concerns | Very low | ["Heterogeneity","Incoherence"] |

**Table 4. Incidence of chronic pain**

| **Comparison** | **Number of studies** | **Within-study bias** | **Reporting bias** | | **Indirectness** | **Imprecision** | **Heterogeneity** | **Incoherence** | **Confidence rating** | **Reason(s) for downgrading** |
| --- | --- | --- | --- | --- | --- | --- | --- | --- | --- | --- |
| LA infusion:No block | 1 | No concerns | | Low risk | No concerns | Major concerns | No concerns | No concerns | Low | ["Imprecision"] |
| LA infusion:PECS-2 block | 1 | No concerns | | Low risk | No concerns | No concerns | Major concerns | No concerns | Low | ["Heterogeneity"] |
| No block:PECS-2 block | 3 | Some concerns | | Low risk | No concerns | No concerns | Major concerns | No concerns | Low | ["Heterogeneity"] |
| No block:PVB | 2 | Some concerns | | Low risk | No concerns | Major concerns | No concerns | No concerns | Low | ["Imprecision"] |
| No block:SPB | 2 | Some concerns | | Low risk | No concerns | No concerns | Major concerns | No concerns | Low | ["Heterogeneity"] |
| PECS-2 block:SPB | 1 | Some concerns | | Low risk | No concerns | Major concerns | No concerns | No concerns | Low | ["Imprecision"] |
| LA infusion:PVB | 0 | Some concerns | | Low risk | No concerns | Major concerns | No concerns | No concerns | Low | ["Imprecision"] |
| LA infusion:SPB | 0 | No concerns | | Low risk | No concerns | No concerns | Major concerns | No concerns | Low | ["Heterogeneity"] |
| PECS-2 block:PVB | 0 | Some concerns | | Low risk | No concerns | Major concerns | No concerns | No concerns | Low | ["Imprecision"] |
| PVB:SPB | 0 | Some concerns | | Low risk | No concerns | No concerns | Major concerns | No concerns | Low | ["Heterogeneity"] |

**Table 5. Incidence of PONV (postoperative 24-hour)**

| **Comparison** | **Number of studies** | **Within-study bias** | **Reporting bias** | **Indirectness** | **Imprecision** | **Heterogeneity** | **Incoherence** | **Confidence rating** | **Reason(s) for downgrading** |
| --- | --- | --- | --- | --- | --- | --- | --- | --- | --- |
| ESPB:PVB | 1 | No concerns | Low risk | No concerns | Major concerns | No concerns | No concerns | Low | ["Imprecision"] |
| ESPB:RIB | 1 | Some concerns | Low risk | No concerns | Major concerns | No concerns | No concerns | Low | ["Imprecision"] |
| ESPB:SPB | 1 | Some concerns | Low risk | No concerns | Major concerns | No concerns | No concerns | Low | ["Imprecision"] |
| ESPB:no block | 5 | No concerns | Low risk | No concerns | No concerns | No concerns | No concerns | High |  |
| IPB:no block | 1 | Major concerns | Low risk | No concerns | Major concerns | No concerns | No concerns | Very low | ["Within-study bias","Imprecision"] |
| LA infusion:PVB | 1 | Some concerns | Low risk | No concerns | No concerns | No concerns | No concerns | High |  |
| LA infusion:no block | 3 | No concerns | Low risk | No concerns | Major concerns | No concerns | No concerns | Low | ["Imprecision"] |
| no block:PECS-1 block | 1 | No concerns | Low risk | No concerns | Major concerns | No concerns | No concerns | Low | ["Imprecision"] |
| PECS-2 block:PVB | 2 | Some concerns | Low risk | No concerns | Major concerns | No concerns | No concerns | Low | ["Imprecision"] |
| PECS-2 block:RIB | 1 | Major concerns | Low risk | No concerns | Major concerns | No concerns | No concerns | Very low | ["Within-study bias","Imprecision"] |
| no block:PECS-2 block | 6 | Some concerns | Low risk | No concerns | No concerns | No concerns | No concerns | High |  |
| no block:PVB | 9 | Some concerns | Low risk | No concerns | No concerns | No concerns | No concerns | High |  |
| RIB:SPB | 1 | Major concerns | Low risk | No concerns | Major concerns | No concerns | No concerns | Very low | ["Within-study bias","Imprecision"] |
| no block:RIB | 1 | Major concerns | Low risk | No concerns | No concerns | No concerns | No concerns | Low | ["Within-study bias"] |
| no block:SPB | 3 | Some concerns | Low risk | No concerns | No concerns | No concerns | No concerns | High |  |
| ESPB:IPB | 0 | Some concerns | Low risk | No concerns | Major concerns | No concerns | No concerns | Low | ["Imprecision"] |
| ESPB:LA infusion | 0 | No concerns | Low risk | No concerns | No concerns | No concerns | No concerns | High |  |
| ESPB:PECS-1 block | 0 | No concerns | Low risk | No concerns | Major concerns | No concerns | No concerns | Low | ["Imprecision"] |
| ESPB:PECS-2 block | 0 | Some concerns | Low risk | No concerns | Major concerns | No concerns | No concerns | Low | ["Imprecision"] |
| IPB:LA infusion | 0 | Some concerns | Low risk | No concerns | Major concerns | No concerns | No concerns | Low | ["Imprecision"] |
| IPB:PECS-1 block | 0 | Some concerns | Low risk | No concerns | Major concerns | No concerns | No concerns | Low | ["Imprecision"] |
| IPB:PECS-2 block | 0 | Major concerns | Low risk | No concerns | Major concerns | No concerns | No concerns | Very low | ["Imprecision"] |
| IPB:PVB | 0 | Some concerns | Low risk | No concerns | Major concerns | No concerns | No concerns | Low | ["Imprecision"] |
| IPB:RIB | 0 | Major concerns | Low risk | No concerns | Major concerns | No concerns | No concerns | Very low | ["Within-study bias","Imprecision"] |
| IPB:SPB | 0 | Major concerns | Low risk | No concerns | Major concerns | No concerns | No concerns | Very low | ["Within-study bias","Imprecision"] |
| LA infusion:PECS-1 block | 0 | No concerns | Low risk | No concerns | Major concerns | No concerns | No concerns | Low | ["Imprecision"] |
| LA infusion:PECS-2 block | 0 | Some concerns | Low risk | No concerns | Major concerns | No concerns | No concerns | Low | ["Imprecision"] |
| LA infusion:RIB | 0 | Some concerns | Low risk | No concerns | Major concerns | No concerns | No concerns | Low | ["Imprecision"] |
| LA infusion:SPB | 0 | Some concerns | Low risk | No concerns | No concerns | No concerns | No concerns | High |  |
| PECS-1 block:PECS-2 block | 0 | Some concerns | Low risk | No concerns | Major concerns | No concerns | No concerns | Low | ["Imprecision"] |
| PECS-1 block:PVB | 0 | No concerns | Low risk | No concerns | Major concerns | No concerns | No concerns | Low | ["Imprecision"] |
| PECS-1 block:RIB | 0 | Some concerns | Low risk | No concerns | Major concerns | No concerns | No concerns | Low | ["Imprecision"] |
| PECS-1 block:SPB | 0 | Some concerns | Low risk | No concerns | Major concerns | No concerns | No concerns | Low | ["Imprecision"] |
| PECS-2 block:SPB | 0 | Some concerns | Low risk | No concerns | Major concerns | No concerns | No concerns | Low | ["Imprecision"] |
| PVB:RIB | 0 | Some concerns | Low risk | No concerns | Major concerns | No concerns | No concerns | Low | ["Imprecision"] |
| PVB:SPB | 0 | Some concerns | Low risk | No concerns | Major concerns | No concerns | No concerns | Low | ["Imprecision"] |
